# A Novel Size-Based Centrifugal Microfluidic Design to Enrich and Magnetically Isolate Circulating Tumor Cells from Blood Cells through Biocompatible Magnetite–Arginine Nanoparticles

**DOI:** 10.3390/s24186031

**Published:** 2024-09-18

**Authors:** Alireza Farahinia, Milad Khani, Tyler A. Morhart, Garth Wells, Ildiko Badea, Lee D. Wilson, Wenjun Zhang

**Affiliations:** 1 Department of Mechanical Engineering, University of Saskatchewan, 57 Campus Drive, Saskatoon, SK S7N 5A9, Canada; 2 Department of Chemistry, University of Saskatchewan, 110 Science Place, Saskatoon, SK S7N 5C9, Canada; 3 Synchrotron Laboratory for Micro and Nano Devices (SyLMAND), Canadian Light Source Inc., 44 Innovation Boulevard, Saskatoon, SK S7N 2V3, Canada; 4 Drug Design and Discovery Group, College of Pharmacy and Nutrition, University of Saskatchewan, 107 Wiggins Rd, Saskatoon, SK S7N 5E5, Canada

**Keywords:** magnetic circulating tumor cell separation, biocompatible magnetite–arginine nanoparticles, inertial microfluidics, centrifugal microfluidics, Dean drag force, microfabrication

## Abstract

This paper presents a novel centrifugal microfluidic approach (so-called lab-on-a-CD) for magnetic circulating tumor cell (CTC) separation from the other healthy cells according to their physical and acquired chemical properties. This study enhances the efficiency of CTC isolation, crucial for cancer diagnosis, prognosis, and therapy. CTCs are cells that break away from primary tumors and travel through the bloodstream; however, isolating CTCs from blood cells is difficult due to their low numbers and diverse characteristics. The proposed microfluidic device consists of two sections: a passive section that uses inertial force and bifurcation law to sort CTCs into different streamlines based on size and shape and an active section that uses magnetic forces along with Dean drag, inertial, and centrifugal forces to capture magnetized CTCs at the downstream of the microchannel. The authors designed, simulated, fabricated, and tested the device with cultured cancer cells and human cells. We also proposed a cost-effective method to mitigate the surface roughness and smooth surfaces created by micromachines and a unique pulsatile technique for flow control to improve separation efficiency. The possibility of a device with fewer layers to improve the leaks and alignment concerns was also demonstrated. The fabricated device could quickly handle a large volume of samples and achieve a high separation efficiency (93%) of CTCs at an optimal angular velocity. The paper shows the feasibility and potential of the proposed centrifugal microfluidic approach to satisfy the pumping, cell sorting, and separating functions for CTC separation.

## 1. Introduction

Cancer metastasis happens when circulating tumor cells (CTCs) travel through the body, invade healthy tissues, and form new tumors [1]. However, the processes of detecting and separating CTCs for subsequent downstream analysis encounter tremendous challenges due to their extremely low abundance in blood samples [2,3,4]. Moreover, the isolated CTCs might be damaged, ruptured, and undergo morphological changes or even degeneration during the separation process, which could potentially impact the accuracy of the information obtained about the primary tumor [5,6,7]. In addition, a majority of the existing methods for CTC isolation from healthy blood cells are based on different physical features, such as size [8,9], density [10], deformability [11], or biological properties; their reaction in acoustic, electric, or magnetic fields; and their resistance to chemical degradation [12]. Some of these techniques present challenges such as low capture efficiency, potential harm to CTCs, reliance on costly and intricate equipment, time-intensive procedures, and the necessity for multiple complex operational protocols [4].

Over the past two decades, microfluidic devices have been recognized as precise and effective instruments for the separation of CTCs from other blood cells [13,14,15]. These devices offer several advantageous features, including low cost, high throughput, minimal sample consumption, portability, high accuracy, and short experiment times [16,17,18,19,20]. Inertial microfluidic techniques, particularly spiral and multi-orifice designs, are notable for their effective size-based separation capabilities. Spiral microfluidic systems are adaptable across different applications by adjusting parameters like cross-sectional area, dimensions, length, and flow rate. Multi-orifice designs, including single- and multiple-chamber configurations, cater to specific needs. The inertial contraction–expansion structure enhances particle manipulation efficiency, aided by secondary flows like Dean flow, which reduces channel length and processing time significantly. Challenges include maintaining optimal flow conditions, controlling cell movement, preventing clogging, ensuring precise operational control, and overcoming the limited flexibility of syringe pumps when integrating preprocessing steps into complex procedures.

Centrifugation methods utilize physical rotational processes on microfluidic platforms, such as centrifugal microfluidics (also known as lab-on-a-disk or lab-on-a-CD) or techniques leveraging liquid centrifugation effects like the Dean effect in systems such as spiral and multi-orifice microfluidics. Various centrifugal microfluidic devices have been created to isolate CTCs, employing principles such as size, density, immunoaffinity, and magnetism [21]. Centrifugal microfluidics utilizes the centrifugal force generated by the rotation of a disk to drive the fluid flow and perform different operations, such as mixing, separation, valving, and detection [21]. When separating CTCs, relying solely on a rotational inertial separator is insufficient because centrifugal force acts uniformly on all cells without distinguishing CTCs from others. This means that while centrifugal force moves cells forward, it does not isolate CTCs specifically. Therefore, additional forces or techniques are necessary to effectively identify and separate CTCs while complementing centrifugation methods. Among the available techniques to integrate with this method, antibody binding to isolate CTCs attached to magnetic nanoparticles (MNPs) is considered particularly effective due to its anticipated accuracy. This approach targets specific cellular characteristics with greater precision compared to size or density-based methods. Antibodies must be initially conjugated with MNPs for this purpose, followed by their mixing with CTCs to induce magnetic field-induced flow diversion. A key benefit of using the magnetic field-based separation method is its non-invasive nature, as it typically allows body cells and tissues exposed to the magnetic field to remain undamaged due to their inherent permeability [22]. Another advantage of employing a magnetic field is its capability to effectively separate large quantities of cancerous cells in a single step. Additionally, a magnetic field can be uniquely utilized on a rotational platform like the LOCD. Unlike the other active separation methods, employing a magnetic field simply involves placing a magnet on the rotational substrate, requiring only that target cells (CTCs) possess magnetic properties to react to the magnetic source. Hence, the binding of antibodies to MNPs and their interaction with CTC antigens play a crucial role in effectively isolating CTCs. As antibodies increasingly attach to MNPs and subsequently to CTC antigens, the separation efficiency under the applied magnetic field improves, as more particles acquire magnetic characteristics. 

The initial successful application of magnetic separation in blood cells involved isolating naturally paramagnetic red blood cells (RBCs) from various cell populations circulating in the bloodstream [23]. Molday et al. utilized polymeric microspheres, chemically coupled to antibodies, to detect and localize antigens and employed the MNPs tagged with fluorescent dyes to isolate lymphocytes and RBCs based on cell surface markers [24]. These early works on separating large cell populations developed multiple magnetite particles and introduced novel magnetic separation methods for various cell types, such as rare CTCs [25]. 

Zhao et al. developed a label-free magnetic isolation technique to separate ferrohydrodynamic CTC based on the size difference cost-efficiently with high throughput [26]. After mixing cancer and blood cells with ferrofluids, the magnetic buoyancy force, induced by the considered magnet close to the channel, deflected the cells from their laminar flow patterns. Indeed, the gravitational force exerted on cells suspended in ferrofluids was proportional to cell volume, resulting in the spatial separation of larger and smaller cells through distinct exit pathways in the microchannel. Recently, they introduced a new label-free microfluidic device for isolating low-concentration CTCs from cell culture lines using ferrofluids, achieving an average separation efficiency of 83% across five different types of cancer cells [27]. Their separation strategy highly relied on the size variation of CTCs with white blood cells (WBCs) in a biocompatible ferrofluid.

The two typical forms of labeled magnetic separation are positive and negative selection. In negative CTC selection, the other blood cells, such as WBCs, are actively depleted from the patient’s blood sample using their particular surface antigens. In the positive selection of CTCs, the low magnetic permeability of cells allows magneto-static forces to selectively separate cells labeled with functionalized MNPs without interference from the surrounding electrolyte solution or other cells [28]. Indeed, antibody-coated MNPs bind to specific antigens on the cell surface. The mixture of cells and MNPs enters the separator, where particles containing the target cells are collected using a magnet [29]. Kang et al. recently designed a disposable chip featuring a microchannel on a versatile substrate fixed with ferromagnetic wires, capable of employing both positive and negative methods for CTC separation by magnetic nanobead-functionalized EpCAM (Epithelial Cell Adhesion Molecule) and CD45/CD66b antibodies under an applied lateral magnetic field [30]. It is worth noting that EpCAM serves as a specific biomarker for isolating CTCs through positive selection. Conversely, biomarkers such as CD133, CD44, and CD45 are specific to blood cells and can be used in negative selection methods.

Chen et al. introduced a size-based microfluidic device with high capture efficiency to separate CTCs [31]. They considered and fixed a few permanent solid magnets under the glass substrate to seize three different magnetized CTCs. Although their device benefited from a high processing rate and a 96% cell viability, the capture efficiency was not enough and decreased over time due to cell accumulation. Before that, Chang et al. introduced an integrated device featuring a Polydimethylsiloxane (PDMS) microfiltration membrane and a parallel flow micro-aperture chip system designed to separate CTCs under controlled flow conditions [32]. The chip successfully captured approximately 89% of CTCs that were coated with antibody-conjugated magnetic beads. It also has advantages such as user-friendliness, reliability, adaptability, and versatility. Later, a study was conducted to assess a spiral-shaped channel device’s selectivity and separation efficiency for two types of CTCs based on EpCAM antigen expression level [33]. Despite achieving over 80% capture efficiency, the device struggled to identify and quantify numerous heterogeneous CTCs with low EpCAM expression. Subsequently, an integrated microfluidic device with a two-step CTC separation process was developed [34]. In the initial step, CTCs were filtered, followed by a second step where they were magnetically separated from a blood sample using anti-CD45 antibody-functionalized magnetic beads on a microfluidic chip. Despite featuring a two-step isolation process designed to minimize cell damage, the device struggled with achieving optimal capture efficiency.

Recently, a microfluidic device equipped with dual antibody functionalization was developed for isolating CTCs. The device, featuring Ni (nickel) micropillars, functionalized with antibodies and Fe_3_O_4_@microbeads, operating under external magnetic conditions on a chaotic herringbone platform. This setup effectively enhanced the detection and isolation of CTCs from blood samples [35]. Yan et al. designed and manufactured a microchip with a micropillar array for electrochemical isolation and analysis of CTCs [36]. They coated the chip surface with a gold layer before a gold-thiol oligonucleotide modification. Meanwhile, they functionalized avidin and EpCAM antibodies. They also enhanced the device by incorporating two gold slices as working electrodes for cell lysis. Cell lysis involves disrupting a cell’s membrane to access and analyze its contents, commonly used in laboratories for purifying or further studying cellular components [37]. The isolated CTCs were electrochemically lysed in this device after applying voltage. 

This paper presents a comprehensive examination of a novel approach that integrates microfluidics with MNPs using antibody binding principles. By combining rotational inertial microfluidic technology with magnetic field influence, the method offers the potential to create a microfluidic separator that improves separation efficiency and addresses the limitations of both active and passive cell separation techniques. The feasibility of implementing this collaborative technique is also explored, requiring a deep understanding of various separation methods, their fabrication processes, and limitations. In this paper, we combine effective methods and introduce a novel size-based centrifugal microfluidic device that can enrich and magnetically isolate CTCs from blood cells through biocompatible magnetite–arginine nanoparticles. The device consists of two main units: a size-based separation unit and a magnetic isolation unit. The size-based separation unit utilizes contraction–expansion microchannel arrays to separate CTCs from blood cells based on their different hydrodynamic behaviors under inertial forces. The magnetic isolation unit employs a permanent magnet to capture CTCs that are labeled with magnetite–arginine nanoparticles, which can enhance the magnetic susceptibility of CTCs without affecting their viability and functionality. The device proposed in this study offers a straightforward, rapid, efficient, and cost-effective means for isolating and analyzing CTCs, potentially advancing the clinical applications of CTCs in cancer research and personalized medicine.

## 2. Materials and Methods

The system, integrating magnetic separation with size-based separation to isolate CTCs from blood cells using Dean drag forces, inertial and centrifugal forces, consists of three main components: (1) the magnetite–arginine nanoparticles, (2) the microfluidic device, and (3) the centrifugal platform.

Magnetite–arginine nanoparticles are the key component that enables the magnetic separation of CTCs from blood cells. The particle-separating function of the system requires several steps and preprocessing because the MNPs–antibody composite should bond with the CTC’s antigen to deliver the magnetic property to CTCs. However, a direct binding between MNPs and a cell’s antigens is not possible due to the lack of functional groups. Therefore, the first step would be modifying MNPs to synthesize the MNPs–antibody composite. Altering MNPs is achieved by forming a shell-core binding between arginine (Arg) and MNPs under a physical bond. Then, the arginine-coated MNPs and antibodies can be chemically bonded through an acid–base reaction, and this biocomposite can be connected to the CTC’s antigen. This is how a cancer cell can possess magnetic properties. The process of producing MNPs and their attachment to arginine and antibodies specific to CTCs are fully explained in Supplementary Materials S1: Material Preparation (S1).

The microfluidic device is the component of the system that is designed in such a way as to use Dean drag forces and perform size-based separation of CTCs from blood cells using inertial and centrifugal forces. The device is designed using fluid mechanics principles and fabricated using photolithography and micromachining techniques. The microfluidic device is divided into two different separation sections: The first half of the design exploits a novel geometry of contraction–expansion arrays (CEAs) for the microchannel connected to a bifurcation region. The second section of the system is designed to separate CTCs from the background cells under inertial and centrifugal effects and bifurcation law and sort them into different streamlines. In addition to a CEA microchannel in the second half of the design, a stack of two on-disk magnets improves the final separation efficiency by capturing the magnetized target cells at the microchannel downstream. In other words, label-free magnetic isolation under the effect of inserted magnets will be performed to separate the target cells from blood cells before entering the final chamber. It should be noted that the device is mounted on the centrifugal platform.

The centrifugal platform is the component of the system that provides the rotational velocities and centrifugal forces for CTC separation. The platform is equipped with a switch motor with double-sided angular velocity control to spin the compact disk (CD) platform in two different rotational directions (see Supplementary Materials S2: Rotating Device (S2)). Due to the device’s rotation in two opposite directions and different properties of cells, the generated vortices and driving forces can move the sample within the channel and separate cells from each other. As one can notice, balancing these forces, choosing suitable magnets, and inserting them at an appropriate distance from the main channel to maximize the separation efficiency are essential and must be carried out carefully; otherwise, CTCs will remain in the main channel while the rest of the cells move through.

## 3. Modeling and Simulation

**Figure 1:**
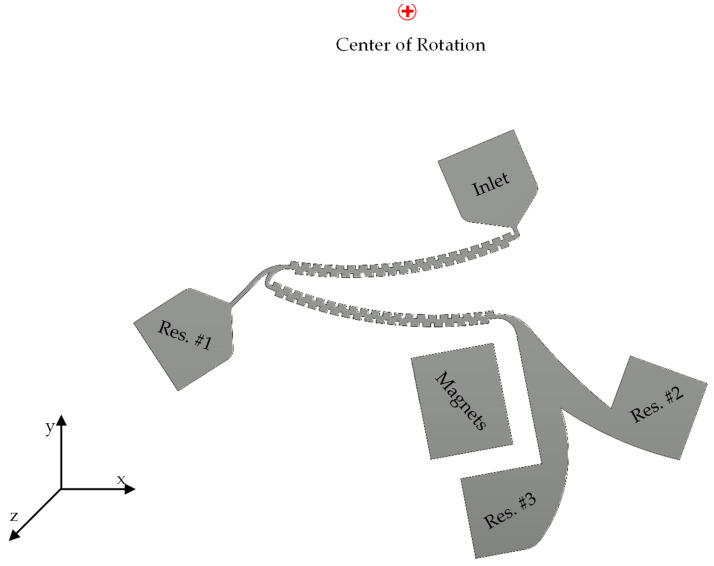
An overview of the designed geometry for the hybrid centrifugal separation system.

The novel centrifugal microfluidic device designed, as shown in Figure 1, consists of two angled channels with multiple contraction–expansion chambers, three outlets connected to three reservoirs, and an inlet. The channel inlet connects to an inclined main channel on a centrifugal platform with multiple contraction–expansion chambers, where the blood sample containing two types of cells will be injected. The main microchannel is connected to a bifurcation region at the end that is extended to two outlets. For a comprehensive understanding of the final design, refer to Supplementary Materials S3: Detailed Design (S3).

### 3.1. Fluid Flow Equations

#### 3.1.1. Continuity Equation

The conservation of mass equation in a fluid flow is defined based on the principle that the mass of a specific group of fluid particles is always constant. The final form of this equation is as follows [38]:

(1) $$\frac{D\rho }{Dt}=\frac{\partial \rho }{\partial t}+u\cdot \nabla \rho =0$$

where ρ is fluid density and t is time. When the fluid is incompressible, this equation becomes as follows:

(2) ∇·u=0

#### 3.1.2. Momentum Equation

The conservation of the momentum equation for a Newtonian fluid with the assumption of continuity, incompressibility, and constant viscosity is as follows [38]:

(3) $$\rho \frac{Du}{Dt}=-\nabla p+\rho g+\mu \nabla^{2}u$$

where g is volumetric force per unit mass, p is pressure, and μ is fluid dynamic viscosity.

#### 3.1.3. Fluid Dynamics on a Rotational Platform

A fluid with a density (ρ) that is placed on a flat surface rotating with an angular velocity (ω) at a distance (*r*) from the rotation axis will experience the following forces [39]:

(4) $$F_{Cen}=\rho rw^{2}\;\mathrm{Centrifugal}\;\mathrm{force}$$

(5) $$F_{Eu}=\rho r\frac{dw}{dt}\;\mathrm{Euler}\;\mathrm{force}$$

(6) $$F_{Co}=2\rho wv\;\mathrm{Coriolis}\;\mathrm{force}$$

The material derivative in Equation (3) will be converted to the following expression (with the assumption of a constant ω) due to the rotation of CD [40]:

(7) $$\frac{Du}{Dt}\approx \frac{Du}{Dt}+\vec{w}\times \left(\vec{w}\times r\right)+2\vec{w}\times u$$

By inserting Equation (7) into Equation (3) and after sorting, the following is obtained:

(8) $$\rho \frac{Du}{Dt}=-\nabla p+\rho g+\mu \nabla^{2}u-\rho \vec{w}\times \left(\vec{w}\times r\right)-2\vec{\rho w}\times u$$

### 3.2. Particle Motion Equations

From the perspective of Lagrangian mechanics, Newton’s second law for particles is expressed as follows:

(9) $$m_{p}\frac{du_{p}}{dt}=\sum \vec{F_{p}}{=F}_{Drag}+F_{Lift}+F_{Pressure\;gradient}{+F}_{Gravity}+F_{Buoyant}+\;F_{Brownian}+F_{Added\;mass}+F_{Basset}+F_{External\;field}+F_{Centrifugal}+F_{Euler}+\;F_{Coriolis\;}$$

where $\sum \vec{F_{p}}$ is the resultant vector of the forces acting on the particle, each of which was fully described in another paper [21]. $u_{p}$ and $m_{p}$ are the particle velocity vector and particle mass, respectively.

It is noteworthy to emphasize that the forces exerted on particles by fluid flow are influenced by the particle’s dimensionless Reynolds number, also referred to as the relative Reynolds number. This parameter is defined as follows:

(10) $${Re}_{p}=\frac{\rho d_{p}\left|u-u_{p}\right|}{\mu }=\frac{{d_{p}}^{2}}{{D_{h}}^{2}}Re=\frac{\rho U{d_{p}}^{2}}{\mu D_{h}}$$

where $d_{p}$, and $D_{h}$ are particle diameter and channel hydraulic diameter, respectively. ρ and μ represent density and viscosity of the fluid, respectively. u and $u_{p}$ are velocity associated with fluid and particle, respectively. Re is also a flow Reynolds number. Based on this number, the aforementioned forces are examined and applied in two distinct scenarios:

- If ${Re}_{p}<1$, the fluid flow surrounding the particle behaves akin to creeping flow.
- If ${1<Re}_{p}<100$, the forces exerted on a particle are notably influenced by fluid inertia.

After simulating the device at an angular velocity of 3000 revolutions per minute (RPM), the average particle Reynolds number within the channel route was equal to ~0.18, while its maximum value was ~0.94.

#### Microfluidic Forces on Particles

The involved forces in centrifugal and inertial microfluidic systems for CTC separation have been recently reviewed and published [21]. All of the forces on the considered particles in this simulation are similar, except for the magnetic force, which only applies to the magnetized CTCs. The following outlines the forces that affect particles in a microfluidic system.

Drag force is as follows: 

The magnitude of drag force varies with the particle Reynolds number [41]:

(11) $$F_{D}=\frac{1}{\tau_{p}}m_{p}(u-u_{p})$$

where $C_{D}$ is the drag coefficient and $\tau_{p}$ is as follows:

(12) $$\tau_{p}=\frac{{4\rho }_{p}d_{p}^{2}}{3\mu C_{D}{Re}_{p}}$$

Here, $F_{D}$ represents the drag force, $m_{p}$ is the particle mass, $u_{p}$ is the velocity vector of the particle, u is the fluid velocity vector, and ${Re}_{p}$ denotes the particle Reynolds number. According to Schiller and Naumann’s proposal, the drag force coefficient is given by [42]:

(13) $$C_{D}=\frac{24}{{Re}_{p}}(1+0.15{{Re}_{p}}^{0.678})$$

Consequently, the total force on the particle is $F_{D}=3\pi \mu d_{p}u(1+0.15{{Re}_{p}}^{0.678})$. 

Lift force is as follows: 

In the case of a neutrally buoyant rigid sphere moving through straight wall-bounded Poiseuille flow, apart from the viscous drag force along the axis, the particle experiences four lateral forces. Among these, Saffman and Magnus forces are often minimal and can be neglected. Thus, the shear gradient lift force and the wall-induced lift force are typically recognized as the primary factors influencing lateral particle migration [43]. The balance between these two effective forces results in multiple equilibrium positions located midway. Under current conditions, Asmolov proposed a relationship suitable for a small rigid sphere (where $d_{p}/H<1$) in a Poiseuille flow [44]:

(14) $$F_{L\;}=\rho G^{3}C_{L}{d_{p}}^{4}$$

His research established that the coefficient $C_{L}$ depends on the lateral position of a particle relative to the microchannel center and the Reynolds number [45].

Pressure gradient force is as follows: 

Pressure gradient force is represented by the following equation:

(15) $${\vec{F}}_{P\;}=m_{f}(\frac{D\vec{u}}{Dt}-v\nabla^{2}\vec{u})$$

where ${\vec{F}}_{P}$ denotes the force resulting from the pressure gradient on the particle, $m_{f}$ represents the fluid mass displaced by the particle (calculated as the product of fluid density and particle volume), and v stands for the fluid kinematic viscosity.

Gravity force is as follows: 

This force for a spherical particle is as follows:

(16) $$F_{G}=\frac{4}{3}\pi R^{3}\rho_{p}g$$

where $\rho_{p}$ and g are particle density and gravitational acceleration, respectively.

Buoyant force is as follows: 

The buoyant force acting on a sphere immersed in a fluid is given by the following:

(17) $$F_{Bu}=\rho_{f}V_{f}g=m_{f}g=W_{f}=\frac{4}{3}\pi R^{3}\rho g$$

where $V_{f}$ stands for the volume of the displaced fluid, $\rho_{f}$ denotes the density of the fluid, $m_{f}$ represents the mass of the displaced fluid, and $W_{f}$ indicates the weight of the displaced fluid. R signifies the radius of the sphere. The combined effect of buoyant and gravitational forces acting on all particles is as follows:

(18) $$F_{Bu,\;G}=(\rho_{f}-\rho_{p})V_{p}g$$

where $\rho_{f}$ represents the density of the fluid, $\rho_{p}$ denotes the density of the particle, and $V_{p}$ signifies the volume of the particle.

Brownian force is as follows: 

Given that the study focuses on particles with diameters larger than 1 µm, the influence of Brownian motion can be neglected.

Added mass force is as follows: 

The calculation of the added mass force for a spherical object submerged in a non-viscous and incompressible fluid is determined as follows [46]:

(19) $$F_{Am}=\frac{\rho_{f}V_{p}}{2}(\frac{Du}{Dt}-\frac{Du_{p}}{Dt})$$

where $\rho_{f}$ and $V_{p}$ denote the density of the fluid surrounding the particle and the volume of the particle, respectively. This volume is equivalent to the volume of fluid displaced as the particle accelerates through the fluid.

The added mass force affects all particles accelerating in a fluid. However, its impact is minimal for dense particles in a low-density fluid due to its dependence on fluid density. This force becomes significant when the fluid density approaches or exceeds the particle density. Given the similar densities of particles and the fluid used in this study, the effects of this force must be considered when examining cell behavior.

Basset force is as follows: 

The impact of this force becomes significant under conditions where temporal changes are notable and diverse. For instance, calculations include the Basset force when analyzing a 10 µm diameter particle in a flow oscillating at a frequency of 700 Hz [47]. Conversely, this effect can be disregarded in the current simulation because there are no significant oscillations in the fluid flow within the microchannel.

External force due to the magnetic field is as follows: 

When a magnetic field is present, it exerts an additional force on particles possessing magnetic properties within a channel. The following equation specifies the magnitude and direction of this new force:

(20) $$F_{Ext}=2\pi {r_{p}}^{3}\mu_{0}\mu_{r,f}K\nabla B^{2}$$

where $K=\frac{\mu_{r,p}-\mu_{r,f}}{\mu_{r,p}+2\mu_{r,f}}$, $r_{p}$ represents the particle radius, $\mu_{0}$ denotes the vacuum permeability, $\mu_{r,p}$ stands for the relative magnetic permeability coefficient of the particle, $\mu_{r,f}$ represents the relative magnetic permeability coefficient of the fluid, and B indicates the magnetic field intensity.

Forces on particles in a rotational platform are as follows:

In centrifugal microfluidic systems, the fluid itself experiences centrifugal, Coriolis, and Euler forces, which also affect the particles within the fluid. Thus, the centrifugal, Euler, and Coriolis forces are defined as follows:

(21) $$F_{Cen,p}=(\rho_{f}-\rho_{p})V_{p}\vec{w}\times (\vec{w}\times {\vec{r}}_{p})$$

(22) $$F_{Eu,p}=m_{p}{\vec{r}}_{p}\times \frac{dw}{dt}$$

(23) $$F_{Co,p}={2m}_{p}\vec{w}\times {\vec{u}}_{p}$$

Each particle’s mass $m_{p}$ is represented here. At a constant angular velocity and a specific radius r, the centrifugal force’s magnitude and intensity surpass those of the other forces, making it the predominant driver of fluid flow within the microchannel. Consequently, microfluidic systems are typically designed radially to harness this propulsive force. Notably, adjusting the angular velocity allows control over all of these forces.

### 3.3. Magnetic Field Equations 

To simulate the magnetic field in a situation like the presented design where there is no free electric current, the following equation must be solved:

(24) $$-\nabla \cdot \left(\mu_{0}\nabla V_{m}-\mu_{0}M\right)=0$$

where $\mu_{0}$, $V_{m}$, and M are the magnetic permeability coefficient of vacuum, magnetic potential, and magnetization, respectively. The magnetic potential distribution is achieved by solving this equation on the simulation domain and considering the magnetization of the used magnet. Based on the following relationship, the magnetic field intensity can be obtained:

(25) $$B=\mu_{0}(M-\nabla V_{m})$$

### 3.4. Boundary Conditions

#### 3.4.1. Conditions Governing the Physics of Particle Motion

The boundary condition at both the inlet and outlet of the microchannel is as follows:

(26) $$p_{inlet}=p_{outlet}=0$$

Furthermore, when the particles collide with the microchannel walls along the channel, it is assumed that these particles return from the wall like a mirror reflection (so-called specular reflection or regular reflection) [48]. This assumption is accurate if the particles are considered entirely rigid, while no energy is lost during the collision with the wall; thus, the collision will be completely elastic [49]. Since the diameter of the particles studied in this study is very small, and they are supposed to be spherical and completely solid, this assumption can be used with a good approximation.

#### 3.4.2. Conditions Governing the Physics of the Magnetic Field

A relatively large domain around the solution domain is supposed to solve the magnetic field, and all of the boundaries of this larger domain become magnetic insulation (i.e., the magnetic field normal to those boundaries is zero):

(27) B·n=0

### 3.5. Physical Properties (Fluid Flow, Magnetic Field, and Particle Tracing)

Since the cells are floating in the plasma section of the blood sample, the desired fluid should have similar properties to plasma for both simulation and experimental purposes [50]. The density and viscosity of blood plasma are ~1025 $Kg/m^{3}$ and 3.5 × 10^−3^ $N\cdot S/m^{2}$ [51]. As the closest option with similar properties to blood plasma, water has a density of 998.2 $Kg/m^{3}$ and viscosity of 1.003 × 10^−3^ $N\cdot S/m^{2}$ [52]. The working fluid for simulation enters the microchannel from the inlet, travels along the microchannel’s curved path while being affected by centrifugal force and a magnetic field, and moves toward the outlet. The ambient pressure (P) and fluid temperature (T) are 1 atm and 300 K, respectively.

The magnetic field is supplied by two permanent magnets embedded in the microfluidic device in the vicinity of the microchannel (close to the target reservoir). The intensity of the required magnetic field was calculated by repeating the simulation. Accordingly, neodymium magnets (NdFeB) with a power grade of N35 and a magnetization intensity (M) of 868 kA/m were considered. Notably, two NdFeB magnets were utilized side by side to improve the separation capacity of the magnet; thus, the intensity of magnetization was doubled in simulating the active section of the device. As a result, the magnetic field generated by permanent magnets applies a force on magnetized particles (CTCs), directing their movement toward the desired outlets. The relative magnetic permeability coefficient of the fluid ($\mu_{r,f}$) and the magnet ($\mu_{r,m}$) and the magnetic permeability coefficient of fluid ($X_{0}$), and nanoparticles (X) are 1.00000037, 3.6, 0, and 0.15 [53], respectively. The magnetic permeability coefficient of vacuum ($\mu_{0}$) is also $4\pi \times 10^{-7}$ H/m [54].

In addition, the cells are modeled as spherical particles immersed in the working fluid. In this simulation, twenty particles have been considered, which are representative of CTCs (target cell) and WBCs (non-target cell). Target cell density ${(\rho }_{p1})$ and its diameter $\left(d_{p1}\right)$ are 1700 $Kg/m^{3}$ [53] and 20 μm [55], respectively, while the non-target cell has a density ${(\rho }_{p2})$ of ~1080 $Kg/m^{3}$ with a diameter $\left(d_{p2}\right)$ of 12 μm [56]. In this case, the formerly introduced forces are imposed on the particles by the fluid, magnetic field, and rotating CD and determine the particle’s movement direction. In this study, COMSOL Multiphysics^®^ software version 5.6 was used.

### 3.6. Results and Discussions

In this simulation, the fluid flow (velocity and pressure distribution) and magnetic field equations have been solved independently of time and in a steady-state situation. After obtaining the velocity and pressure distribution, the movement of the particles is projected by injecting them and applying forces from the fluid or external fields. However, the particle tracing equations are time-dependently solved to calculate the particle movement and location at any time. A sufficient time range has been chosen by repeating the simulation so as to solve the particle-tracing equations. Since the required time that the released particles reach the microchannel outlet from its inlet will differ at various angular velocities, the duration of each simulation is estimated by trial and error. In addition to solving the fluid flow equations in the active section of the microfluidic device, the magnetic field equations must also be solved to obtain the external field (produced by the embedded permanent magnets) to be used in the effective forces on the target particles. Furthermore, since the performance of the proposed design is based on Dean or secondary flow, which exhibits itself in the third dimension, all simulations must be performed in 3D. Moreover, the accuracy of the numerical solution for the Navier–Stokes equations using the finite element method (FEM) was increased by choosing P1-P2 in the discretization section of the equations in COMSOL^®^ to model the secondary flows inside the microchannel accurately.

It should be mentioned that due to the large amount of mesh required in active and passive sections of the device, a high-processing-power computer with a massive amount of random access memory (RAM) is necessary. Therefore, the device is supposed to be divided and examined in two completely separate sections to carry out the simulation based on the available facilities. First, the fluid flow along with the suspended particles is simulated in the first passive section, which includes a microchannel rotating clockwise on a platform. Then, the fluid flow and suspended particles, partially separated in the first stage, are simulated in the second section. This active section includes the microchannel rotating counter-clockwise on the platform to investigate the magnetic field effect on cell separation. The inlet boundary condition for the microchannel in the second section for solving the fluid flow equations is a constant inlet flow rate, whose value is equal to the flow rate measured from the passive section in the interface cross-section. The initial position and speed of the particles in the second section are also approximately assumed to be equal to the position and speed of the particles when passing this interface cross-section. Despite the fact that these simplifications and changes to the boundary conditions may lead to a deviation from the actual case, it should be noted that the purpose of the current simulations is to observe the magnetic field effect on cell separation.

**Figure 2:**
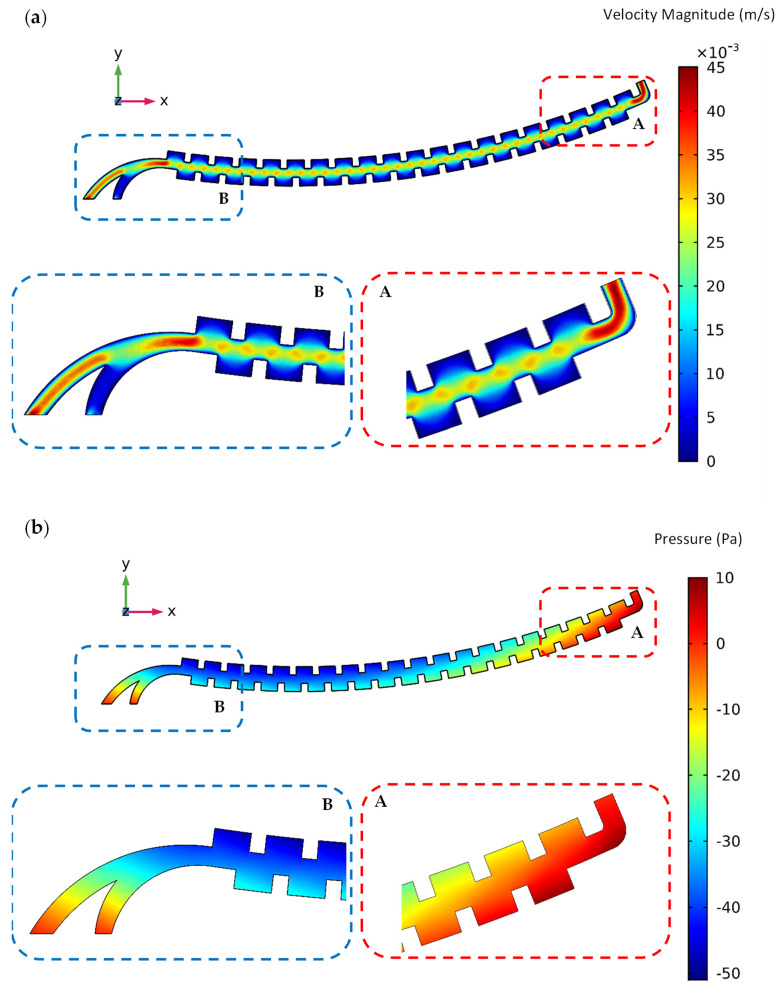

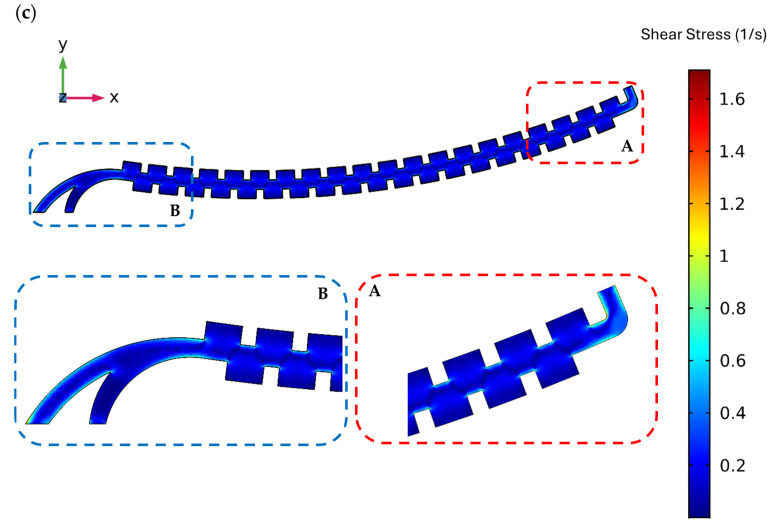
(**a**) Velocity, (**b**) relative pressure, and (**c**) shear stress distribution contours of the fluid flow in the passive section of the centrifugal microfluidic system at an angular velocity of 2500 RPM.

The order of simulation and the numerical solution is that the governing equations of the fluid are first solved, and the velocity distribution, shear stress, and fluid pressure are then calculated. Indeed, the flow field should be modeled in the first step because the flow field and the velocity profile must be fed into the software to calculate the drag force in the particle tracing module and acquire the initial velocity vector of the particles at the beginning of tracing. For example, these results for the first passive section at the angular velocity of 2500 RPM are shown in Figure 2a–c. As shown in Figure 2a, it is clear from the velocity distribution that the fluid velocity increases in the narrow areas of the channel (where the channel width decreases), which causes the particles to accelerate in these regions.

**Figure 3:**
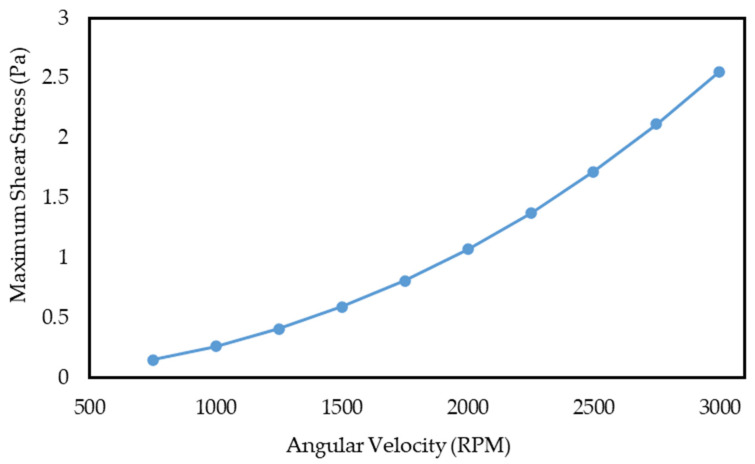
The diagram of the maximum shear stress calculated in the fluid flow based on the angular velocity of the first section of the centrifugal microfluidic system.

It should be noted that fluid shear stress plays a considerable role in the process of cell separation because cells are highly susceptible to shear stress. Indeed, if the shear stress on cells exceeds an acceptable limit during their movement inside the fluid, their membranes are damaged and ripped (so-called “cell apoptosis”). The critical shear stress value to remain CTCs healthy is reported to be about 4.5 Pa [57,58,59]; thus, it is advised not to exceed this limit. As shown in the diagrams in Figure 3, the maximum shear stress resulting from the fluid flow in the solution domain was extracted at different angular velocities. According to this figure, the shear stress in the first section of the centrifugal microfluidic system peaks at roughly 2.55 Pa at an angular velocity of 3000 RPM. Based on the critical limit, this value is safe, and the cells can be investigated after separation due to not being damaged during experiments.

**Figure 4:**
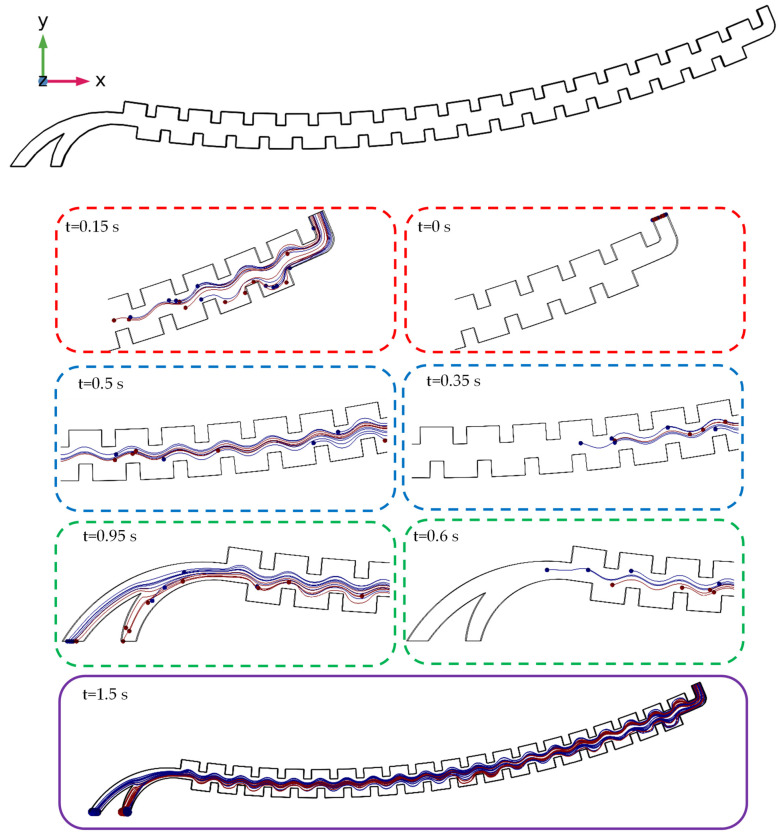
The results of the simulation of the first section of the centrifugal microfluidic device and the movement of the cells at different times at an angular velocity of 2500 RPM.

After solving the governing equations of the fluid flow in the first section and using their results, the position of the cells is estimated and simulated at different times, and their movement path is determined until reaching the considered outlets. The results of this solution for the angular velocity of 2500 at different times can be seen in Figure 4. In addition, as the dimensions of the cells are microscopic compared to those of the microchannel, their shown size in the results is a multiple of their actual size so that the location of the particles becomes clearly visible at each cross-section of the microchannel. It should be noted that these two types of cells were injected with a random distribution at the microchannel entrance. The blue spheres represent non-target cells, while the red ones are interpreted as target cells. As shown, during the movement of the particles inside the channel, different amounts of centrifugal forces were exerted on the particles due to their different distances from the center, sizes, and densities, which increased the distance between the two particles.

The separation capability of the centrifugal microfluidic system is eventually evaluated and presented by calculating the separation percentage of target cells (CTCs) from non-target cells (blood cells) and analyzing the simulation results. The separation percentage of target cells from non-target cells is calculated in the reservoir/outlet assigned for the target cells. The separation efficiency (η) is calculated by dividing the number of separated cells by the total number of cells. As such, the optimal state for the separation is achieved if the separation percentage of target cells and non-target cells in the target reservoir/outlet is maximum and minimum, respectively. Based on this evaluation method, the separation percentage of target CTCs and the separation percentage of non-target blood cells in Reservoir #1 were counted and are shown in Table 1.

The changes observed in the graphs in this figure can be analyzed after investigating the amounts of applied forces. Since the flow direction is approximately tangential to the angular direction, and since the target reservoir gate is positioned closer to the radial direction of CD than the non-target reservoir gate, the average flow rate in the main microchannel is low at slow angular velocities. Thus, the centrifugal force has a stronger effect than the Coriolis force. Therefore, the target and non-target cells are more launched in the radial direction outwards from the center, which, in turn, causes their concentration in the target outlet (to the active section) to be higher than in the non-target reservoir (Reservoir #1). In the following, when the average flow rate in the main microchannel is increased due to the increase in the angular velocity, the value of the Coriolis force becomes significant. As a result, the tendency of cells from the target outlet is diminished and shifted to the non-target reservoir. According to Table 1 and the principles mentioned to determine the optimal conditions, the best separation performance of the first passive section of the centrifugal microfluidic system is achieved at a clockwise angular velocity of 2500 RPM. Based on these results, if the desired angular speed is attained in the specified time range under ideal conditions, the optimal separation percentage of target CTCs is 20%, mixed with 80% of non-target cells. This means that most CTCs have entered the active section, and a few were stuck in the non-target reservoir. Moreover, although the separation percentage of target CTCs is less than 20% (i.e., more CTCs entered the active section) at an angular velocity of more than 2500 RPM, that of non-target cells is also decreased in the non-target reservoir, which, in turn, implies that more non-target cells enter the active section, and it is not favorable. Indeed, the passive unit is supposed to let the maximum number of CTCs and the minimum number of blood cells enter the active part or let the minimum number of CTCs and the maximum number of blood cells enter Reservoir #1.

**Figure 5:**
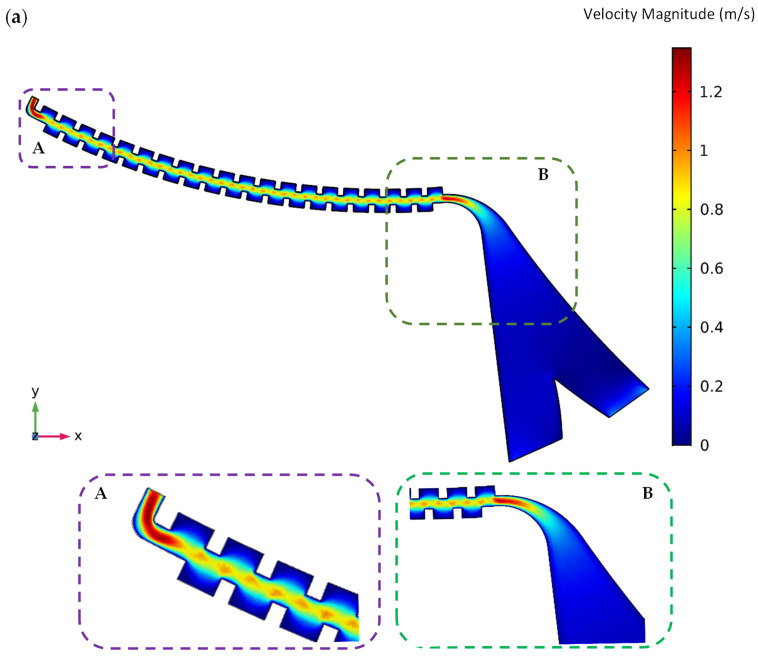

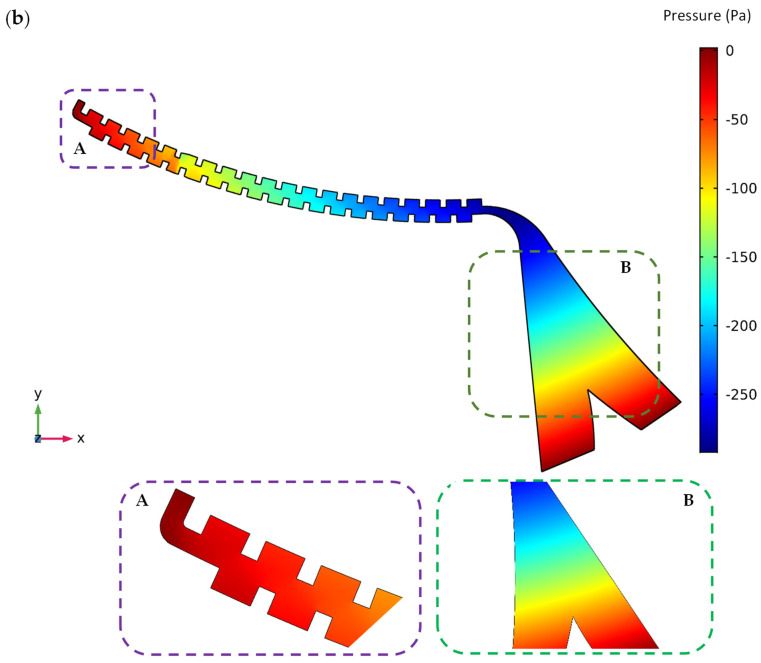

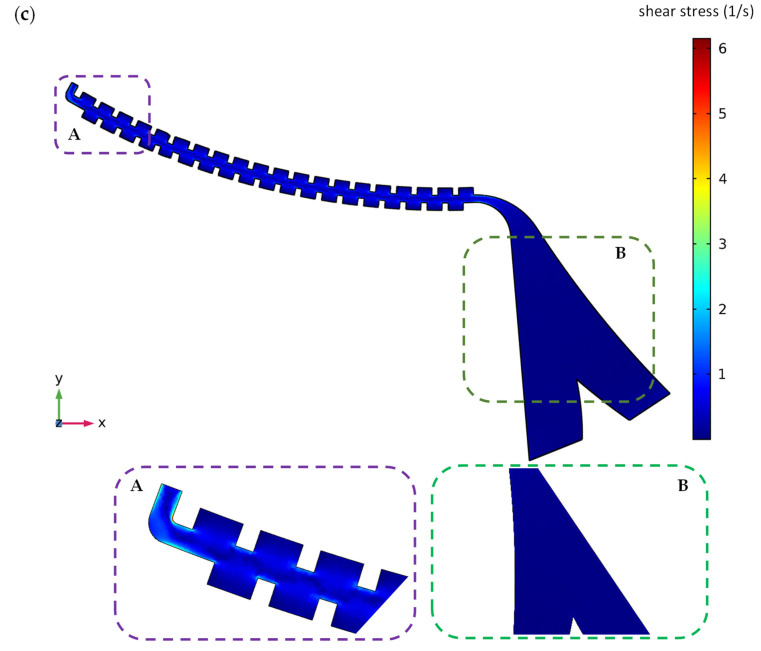
(**a**) Velocity, (**b**) relative pressure, and (**c**) shear stress distribution contours of the fluid flow in the active section of the centrifugal microfluidic system at an angular velocity of 2500 RPM.

Similarly, a simulation of the active section of the device was also performed at different angular velocities. Moreover, the duration of each simulation was determined by trial and error. The order of simulation and the numerical solution is the same as the previous one. The velocity distribution, shear stress, and fluid pressure for this active section at an angular velocity of 2500 RPM are shown in Figure 5a, Figure 5b, and Figure 5c, respectively.

**Figure 6:**
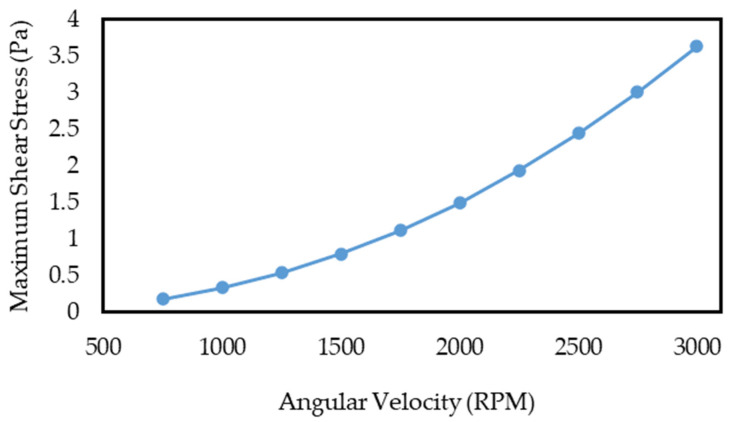
The diagram of the maximum shear stress calculated in the fluid flow based on the angular velocity of the active section of the centrifugal microfluidic system.

Similarly, the maximum shear stress resulting from the fluid flow in the solution domain at different angular speeds is extracted and shown in Figure 6. Based on this figure, the shear stress in this section of the device at a rotational speed of 3000 RPM rises to about 3.63 Pa, much higher than in the passive separator system; however, it does not exceed the allowable critical limit.

**Figure 7:**
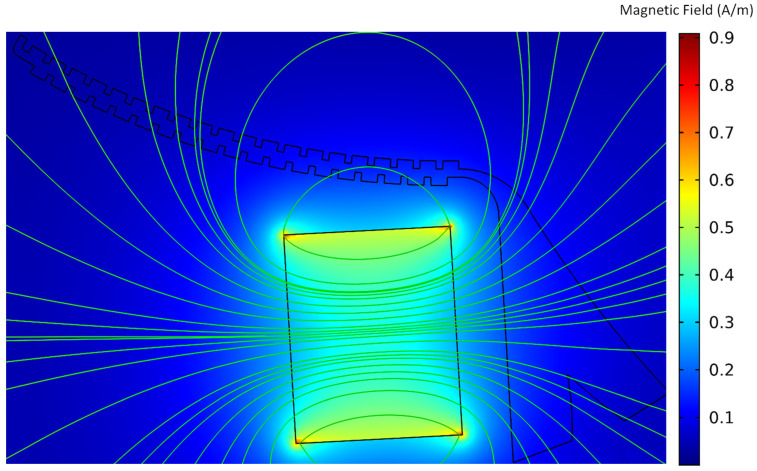
The contour of the magnetic field intensity distribution resulting from magnets and equipotential lines in the centrifugal microfluidic system.

Next, the magnetic field generated by the permanent magnets in the area where the microchannel is located near their center was simulated, and the magnetic field intensity distribution in this area is shown in Figure 7. In magnetic field simulation, commercially available 35-grade neodymium magnets were considered the magnetic field generation source. The magnet was placed at a 1.5 mm distance from the microchannel because it was found that the maximum magnetic flux density forms in the vicinity of the magnets, near the microchannel bifurcation region. In this case, the magnetic force applied to the magnetized particles reaches its maximum value. Indeed, as will be evident in the next figures, the magnetic field intensity at the bifurcation area of the microchannel causes the particles to separate from each other under the magnetic force.

**Figure 8:**
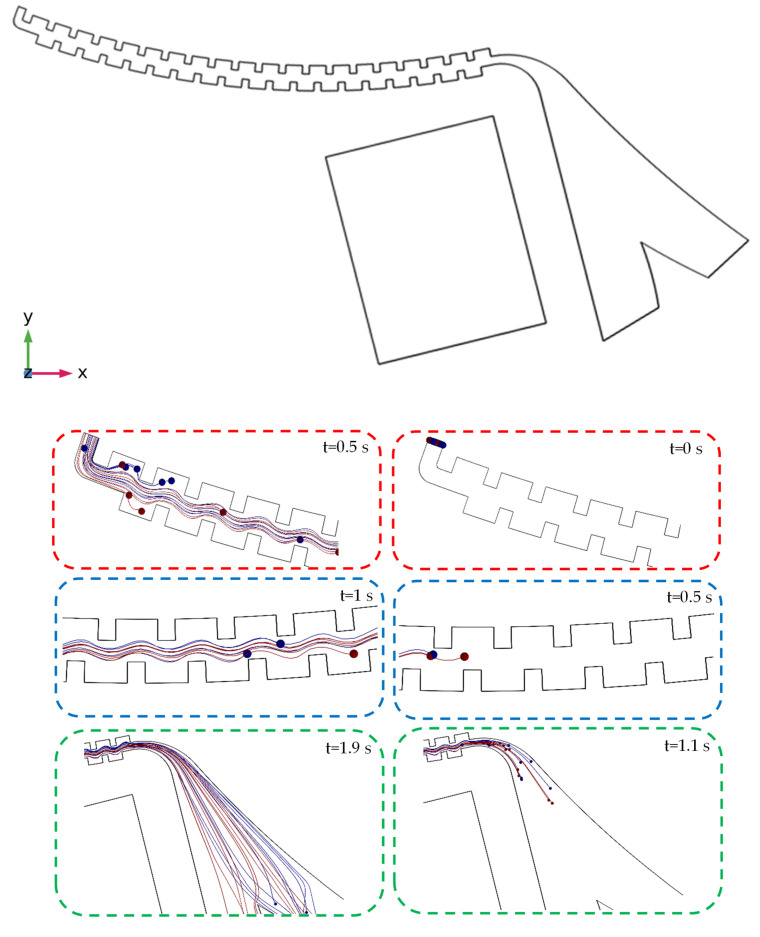

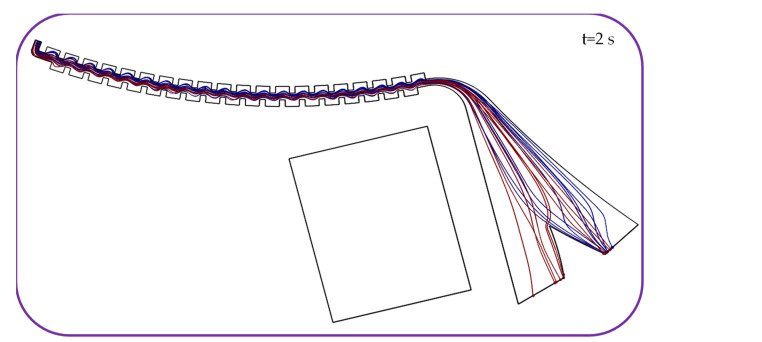
The results of the simulation of the second section of the centrifugal microfluidic device and the movement of the cells at different times at an angular velocity of 2500 RPM.

After solving these two physics phenomena and using their results, the position of the cells was estimated and simulated at different times, and their movement path was determined until reaching the considered outlets. The results of this solution for an angular velocity of 2500 RPM at different times can be seen in Figure 8. It should be mentioned that the results of the flow field distribution (velocity and pressure values in the geometric domain) and the magnetic field (the magnetic field intensity) were used to calculate the forces (by the fluid and magnets) acting on the particles.

Similar to the evaluation method mentioned in the simulation of the passive section, the separation percentage of target cells from non-target cells was counted in the target reservoir (Reservoir #3) in the active section and is shown in Table 2. According to this figure and the principles mentioned to determine the optimal conditions, the best separation performance of the second active section of the centrifugal microfluidic system was achieved at a counter-clockwise angular velocity of 1750 RPM. In addition, based on these results, if the desired angular speed is attained in the specified time range under ideal conditions, the optimal separation percentage of target CTCs is 100%, mixed with 0% of non-target cells as impurities. The separation percentage at this stage can be regarded as the final separation efficiency of the microfluidic device.

## 4. Fabrication of the Microfluidic Separator

In this study, a variety of microfabrication techniques from soft lithography to 3D printing were studied; however, according to the type of design, the microchannel aspect ratio, and some other critical parameters, only two of them could be successfully applied. These two techniques are presented in Supplementary Materials S4: Fabrication (S4).

**Figure 9:**
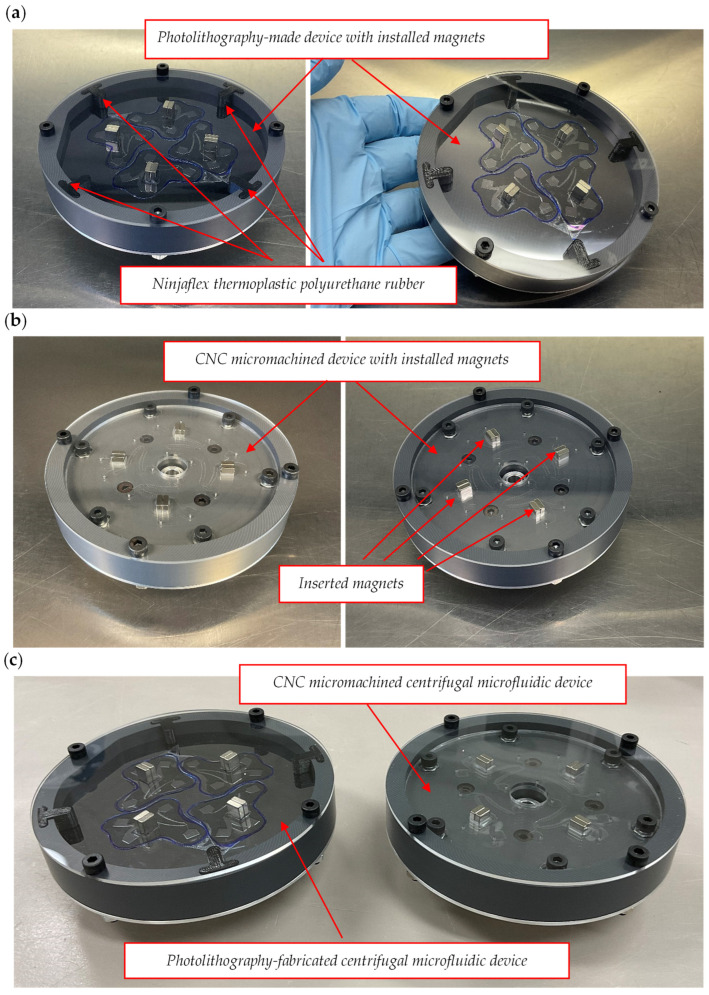
Microfluidic devices fabricated through two different microfabrication techniques; (**a**) the centrifugal microfluidic separation device designed by Fusion 360^®^ and fabricated by a photolithography process; (**b**) the centrifugal microfluidic separation device designed by Fusion 360^®^ and fabricated by CNC micromachine; (**c**) photolithography-fabricated and CNC-micromachined centrifugal microfluidic devices.

The final images of these centrifugal systems are shown along with their holders in Figure 9a–c. Figure 9a,b show the photolithography- and computer numerical control (CNC) micromachine-fabricated centrifugal microfluidic devices, which were successfully assembled and were entirely kept inside the holders. Figure 9c also indicates a glance view of these two devices side by side.

### Quantitative Measurement of the Results

To quantify the results and distinguish target cells from non-target cells, one of the injected cells should be painted. Accordingly, a type of cell nucleus-binding blue-fluorescent dye called DAPI (4′,6-diamidino-2-phenylindole) was used. With a maximum excitation wavelength of 359 nm and a maximum emission wavelength of 461 nm, DAPI emits blue light when exposed to UV light [60]. Indeed, it is a kind of stain mainly utilized in fluorescence microscopy, flow cytometry, and chromosome staining.

In this study, as the ultimate objective is the genetic study of CTCs, and this fluorescent dye binds to the DNA of the nucleus, the non-target cells should be stained. Thus, a group of non-target cells (L929 in this study) were dyed with fluorescent dye to make a difference in distinguishing target from non-target cells during the examination.

After the experiments and separation tests, the samples (including the target cells and stained non-target cells) were separately extracted from each target and non-target reservoirs and placed under fluorescence microscopy for further investigation. A number of images were obtained by taking one optical image and one fluorescence image of each area and repeating this process for several areas of each sample. As expected, all of the cells were observable in the optical images, while only non-target DAPI-colored cells could be seen in the fluorescence images. As such, the approximate number of the target and non-target cells in each reservoir was determined by individually counting the target and non-target cells from the images related to different regions for each reservoir. As a result, it was possible to estimate the separation efficiency/percentage of the target cells, similar to the method used for calculating the simulation results.

## 5. Experiments

This section presents the experiment on the separation of target CTCs (MCF-7 in this study) from non-target cells (L929 in this study). The cell culture process is presented, where, first, the reliability of the attachment of CTCs to antibodies is checked and then the accuracy of the connection between Arg-capped IONPs (Iron Oxide Nanoparticles) and CTCs is examined. Details of Arg-capped IONPs are mentioned in Section S1.2 in S1. The result of the experiment of the separation of MCF-7 and L929 is presented along with discussions in the next section. This result also gives an evaluation of the proposed devices in terms of their separation efficiency. The study of the effect of different ratios of IONPs to Arg on the separation performance is also presented. The process of cell culturing and positive and negative tests to inspect whether the antibodies attach to MCF-7 and whether the antibodies do not connect to non-target cells, as well as the test for checking device functionality, are introduced in Supplementary Materials S5: Experiment/Test (S5).

### Experiment of the Separation of MCF-7 from L929 Populations

**Figure 10:**
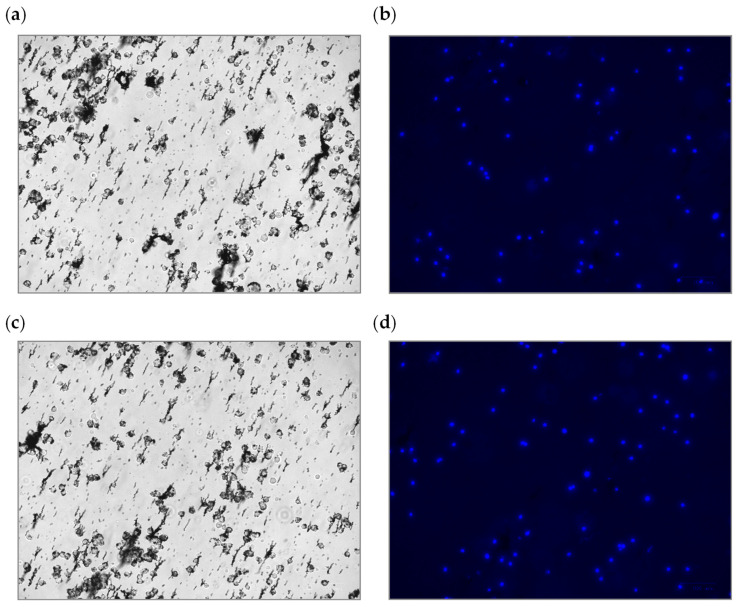
Photomicrographs of a region of the extracted sample from the target reservoir of the photolithography separation system with (**a**) an optical microscope and (**b**) a fluorescent microscope; photomicrographs of a region of the extracted sample from the target reservoir of the micromachined separation system with (**c**) an optical microscope and (**d**) a fluorescent microscope. Note that L292 cells were dyed with a fluorescent dye.

**Figure 11:**
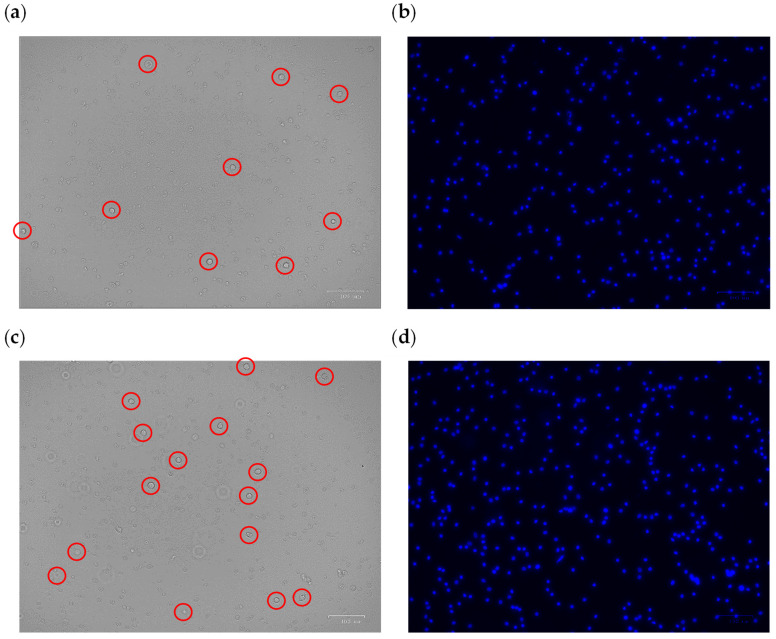
The image captured from a region of the extracted sample from the non-target reservoir of the photolithography separation system with (**a**) an optical microscope and (**b**) a fluorescent microscope; the image captured from a region of the extracted sample from the non-target reservoir of the micromachined separation system with (**c**) an optical microscope and (**d**) a fluorescent microscope. Note that the marked red circles demonstrate CTCs in this reservoir.

The experiment was primarily conducted by injecting a specific ratio of target cells and colored non-target cells into the inlet reservoir. To make the experiment closer to reality, the number of non-target cells was considered five times that of target cells. This means that 1 million MCF-7 cells (i.e., CTCs in the blood) along with 5 million L929 cells (the representation of WBCs in the blood) were injected into the inlet reservoir. The experiment was conducted with the two developed separators in this study: (i) centrifugal photolithography-based and (ii) CNC-micromachined separators. The experiment started with a clockwise rotation of 2500 RPM and ended at a counter-clockwise rotational speed of 1750 RPM. In addition, the experiment was repeated five times at each velocity to come up with the repeatability of the result. After each run, the content of Reservoirs #2 and #3 was emptied, observed under the microscope, and investigated by comparing the taken optical and fluorescent images (examples are shown in Figure 10 and Figure 11).

The experimental results were then quantified according to the method explained in Section “Quantitative Measurement of the Results”. Quantifying the results and determining the device efficiency can be possible through the initial counting of cells (before injection) and counting them after extracting the sample from each outlet reservoir on the counting chamber. After each run of the experiment, it is worth mentioning that the microchannels should be rinsed with Phosphate-Buffered Saline (PBS) and trypsin (an enzyme used to detach cells from the bottom of the cell culture container) to extract the residual cells in the system and prepare the device for the next test.

## 6. Results and Discussion

The experimental results are shown in Table 3 and Table 4. It can be found that the separation efficiency of the target cells for the photolithography-based microfluidic device was 89.1 ± 3.7% along with 8.4 ± 3.6% impurity resulting from the presence of non-target cells (Table 3). In comparison, the separation efficiency of the target cell for the micromachine-based microfluidic device was 84.6 ± 5.2% with 11.5 ± 4.9% impurity (Table 4). Further from these tables, it is evident that the percentage of the non-target cells detected in the target reservoir is, to some extent, consistent with the simulation result. However, the number of the separated target cells in the target reservoir with the photolithography-made device does not precisely match the simulation result and has an approximately 14.5% margin of error. This discrepancy in the micromachine-based separation system is approximately 6.0% more.

There might be several reasons that lead to this error. First, choosing the limited number of simulated particles (due to high and unfeasible computational costs and the available RAM on the computer) could be one of them. Indeed, in the simulation, not all of the possible points from which the cells were released at the entry were considered, which led to the discrete assessment of the separation efficiency in the simulation result. Second, in the simulation, the particle diameter to hydraulic channel diameter ratio was very close to the critical value reported for effective inertial separation. This was because of the lack of a thinner cutting tool and, consequently, no possibility of creating microchannels with a smaller hydraulic channel diameter. Third, the rotational velocity of the platform was assumed to be constant in the simulation. This assumption was not entirely accurate because the centrifugal platform was accelerated from a stationary condition to the desired speed with the driving motor. Indeed, the constant velocity assumption is more dependent on the inherent capabilities of the driving motor for creating a steady-state rotational movement. Due to the non-stationary condition in this study, not only was the Eulerian volumetric force applied to the fluid flow, but also some oscillations were applied to the disk at the beginning of the rotation. In consequence, the actual results partially diverged from the simulation predictions. Fourth, the microchannel walls also suffered surface roughness due to the chosen microfabrication technique (especially for CNC micromachining). Although the direct spraying of vaporized acetone could significantly smooth the bottom of the microchannels, it had little effect on their wall. Moreover, the vaporized acetone polishing method could be only effective to a certain extent. Therefore, the remaining surface roughness affected the fluid flow and, consequently, the movement of cells. Fifth, the Lagrangian method was used to simulate the movement of cells. Under this approach, the cells/particles were viewed as zero-dimensional points, which, in turn, led to the effect of cells/particles on the fluid flow and each other being overlooked. Sixth, all of the considered assumptions for the simulation, such as the assumption of the rigidity of the cells and their lack of influence on each other during the movement within the microchannel, caused errors that might deviate the simulation results from reality.

Figure 10a–d and Figure 11a–d depict the images to quantify the experimental results from the microfluidic devices. Figure 10 shows a target reservoir region containing CTCs (non-colored cells). The comparison between the images obtained from the optical and fluorescence microscope (which reveals only non-target dyed cells) indicates that most target cells have entered the target reservoir, and a small number of non-target cells are seen in this area. This resulting phenomenon is true for both devices, with the difference that the photolithography-based device performed better than the other in separating the target cells and pushing them toward the corresponding target reservoir. Figure 11 also shows the non-target reservoir to which the non-target cells should be directed. Similarly, comparing the optical and fluorescent images demonstrates that a few CTCs (marked by the red circles in the optical image) entered this reservoir. This resulting phenomenon is also true for both fabricated devices; however, the photolithography-based device had a better performance than the other device in dragging non-target cells to the respective non-target reservoir. Overall, the photolithography-fabricated microfluidic device forced more target cells into the target reservoir and more non-target cells into the non-target reservoir.

The effect of different combination ratios of IONPs to Arg (1:1, 1:3, 3:1, 1:20, and 1:50, synthesized in Section S1.2 in S1) on final cell separation under the magnetic field is examined in Table 5. As shown in this table, the sample with a ratio of 1:50 indicates the best performance compared to the other samples. As such, the high IONPs/Arg ratios (e.g., 1:50 compared to 1:1) provided more opportunities for IONPs to bond with antibodies; as a result, more CTCs could be bonded to IONPs-attached antibodies. However, this should not be taken to mean that the higher the ratio of Arg, the better the result. Indeed, at the very early stages of combining these two components (IONPs and Arg), it was found that a ratio higher than 1:50 greatly affected the magnetic performance of IONPs in the presence of a magnetic field, which, in turn, would reduce the number of deviated CTCs towards the target outlet.

Furthermore, the examination of different ratios of IONPs/Arg in Table 5 reveals that increasing the number of IONPs, for example, by thrice the number of Arg (i.e., 3:1), does not enhance the separation efficiency by itself and even decreases it below the separation efficiency with 1:1 ratio. According to the experience of cell magnetization so far, this is true because increasing the number of IONPs does not lead to an increase in the number of magnetized CTCs. Indeed, the presence of sufficient Arg can ensure the attachment of IONPs to antibodies and then the cell’s antigen. Excess IONPs that could not make bonds to CTCs would be finally absorbed by the on-disk magnets and accumulated in the target reservoir as a black mass. Consequently, the presence of excess IONPs should not be thought of as having a chance to benefit from more magnetized CTCs and a better separation rate. Increasing or decreasing IONPs without considering enough Arg cannot improve the separation efficiency; indeed, there is an optimal ratio of IONPs to Arg that can maximize the final efficiency.

In addition, from Table 5, the sample with a ratio of 1:3 does not follow a logical trend like the other samples. There could be numerous reasons behind this inconsistency. First, Arg might not be well combined with IONPs in the early stages (refer to Section S1.2 in S1), which can be checked through Raman spectroscopy and TGA analysis. Second, the carboxylic acid groups in Arg, responsible for making the bond with the antibody, were not correctly activated (refer to Section S1.3 in S1). Finally, the bonds between the cell’s antigen and IONPs-attached antibodies were not strongly formed (refer to Section S1.4 in S1). As one can notice, any mistakes in any preceding steps can, directly and indirectly, affect the final separation efficiency, showing the high sensitivity of cellular work compared to other laboratory work.

## 7. Conclusions

This study aimed to develop a novel hybrid centrifugal microfluidic technology along with specific devices to efficiently and selectively capture and separate CTCs from blood cells under various physical and biological procedures with the help of a rotating platform and magnetic nanoparticles (functionalized with arginine that can bind to CTCs). The study had a high level of complexity and challenge, such as synthesizing and functionalizing the nanoparticles; designing, simulating, fabricating, and testing the microfluidic devices; designing and assembling the switch motor with double-sided angular velocity control to spin the CD platform; selecting the material of the device that contacts human cells; considering the maximal stress in the cell; investigating the centrifugal parameters; and validating the performance of the device with cultured cells.

Taking everything into account, applying the contraction–expansion arrays with integrating magnetophoretic techniques in the centrifugal microfluidic device enhanced the recovery rate of target MCF-7 cells from L929 cells compared to passive technologies. The enhancement in the photolithography-fabricated microfluidic device was related to its unique fabrication process. This unique feature provided a straightforward method with low surface roughness, which, in turn, affected cells and their movements less. Therefore, making the final microchannels with a photolithography method was much easier than the micromachining approach, which needed several postprocessing steps (such as bonding layers, smoothing the channel surface, and so on).

The possibility of a device with fewer layers to improve the leaks and alignment concerns was demonstrated. Unlike the other centrifugal microfluidic systems, the design solutions could reduce the number of layers of the device to two, which, in turn, diminished the possibility of leakage. In addition, different ratios of IONPs to Arg were examined, which revealed that although the magnetic strength of IONPs would be limited due to being surrounded by many Arg particles, the presence of more Arg provided more chances for CTC attachments. Therefore, it can be concluded that the ratio of 1:50 (IONPs to Arg) was associated with the highest separation effectiveness. This finding was contrary to popular belief, which was focused on the negative aspect of raising such an IONPs: Arg ratio. Meanwhile, it is worth noting that the placement and distance of the magnets near the outlet chambers play an essential role in this separation.

The research in this paper has concluded that such a technology is feasible and promising. The novel centrifugal microfluidic device can be employed in cancer diagnostics to create an integrated microfluidic platform with the other operational units, such as those for mixing and lysis of the cell membrane.

## Figures and Tables

**Table 1:** Separation percentage of target cells from non-target cells in the non-target reservoir (Reservoir #1) at different angular velocities of the first section of the microfluidic system.

	Recovery in Reservoir #1 (%)	
Angular Velocity (RPM)	Target Cells	Non-Target Cells
750	50	100
1000	50	100
1250	50	100
1500	50	100
1750	40	100
2000	40	100
2250	30	90
2500	20	80
2750	20	70
3000	10	70

**Table 2:** Separation percentage of target cells from non-target cells in the desired reservoir (Reservoir #3) at different angular velocities of the second section of the microfluidic system.

	Recovery in Reservoir #3 (%)	
Angular Velocity (RPM)	Target Cells	Non-Target Cells
750	100	20
1000	100	10
1250	100	10
1500	100	10
1750	100	0
2000	80	0
2250	70	0
2500	60	0
2750	60	0
3000	50	0

**Table 3:** The experimental results of the photolithography-based separation device and its comparison with the simulation results.

	Recovery in Reservoir #3 (%)			
Angular Velocity (RPM)	Target Cells (Simulation Result)	Non-Target Cells (Simulation Result)	Target Cells (Experimental Result)	Non-Target Cells (Experimental Result)
750	100	20	-	-
1000	100	10	-	-
1250	100	10	-	-
1500	100	10	-	-
1750	100	0	89.1 ± 3.7	8.4 ± 3.6
2000	80	0	-	-
2250	70	0	-	-
2500	60	0	-	-
2750	60	0	-	-
3000	50	0	-	-

**Table 4:** The experimental results of the micromachine-based separation device and its comparison with the simulation results.

	Recovery in Reservoir #3 (%)			
Angular Velocity (RPM)	Target Cells (Simulation Result)	Non-Target Cells (Simulation Result)	Target Cells (Experimental Result)	Non-Target Cells (Experimental Result)
750	100	20	-	-
1000	100	10	-	-
1250	100	10	-	-
1500	100	10	-	-
1750	100	0	84.6 ± 5.2	11.5 ± 4.9
2000	80	0	-	-
2250	70	0	-	-
2500	60	0	-	-
2750	60	0	-	-
3000	50	0	-	-

**Table 5:** The experimental results of different ratios of IONPs/Arg using the photolithography-fabricated separation device rotating clockwise and counter-clockwise (at 2500 and 1750 RPMs, respectively).

Ratios of IONPs/Arg	Separation Efficiency of Target Cells (%)
1:1	69.1 ± 7.0
1:3	58.8 ± 8.8
3:1	40.2 ± 5.7
1:20	82.5± 5.4

## Data Availability

All data analyzed or generated during the study have been included in this published article.

## Supplementary Materials

The following supporting information can be downloaded at: https://www.mdpi.com/article/10.3390/s24186031/s1. The details are organized as follows: Supplementary Materials S1: Material preparation (S1): This document elaborates on the synthesis of magnetite–arginine nanoparticles (MNPs), their modification to create MNPs–antibody composites, and the bonding process with CTCs. The MNPs are essential for the magnetic separation of CTCs from blood cells, and their preparation involves several steps to ensure proper binding with CTC antigens [61,62,63,64,65,66,67,68,69,70,71,72,73,74,75,76,77,78,79,80,81,82,83,84,85,86,87,88,89,90,91,92,93,94,95,96,97,98,99,100,101,102,103,104,105,106]. Supplementary Materials S2: Rotating device (S2): This document describes the centrifugal platform, including its design and functionality. The platform’s rotational velocities and centrifugal forces are critical for the effective separation of CTCs. The dual-directional rotation mechanism and its impact on cell separation efficiency are detailed. Supplementary Materials S3: Detailed design (S3): This document provides a comprehensive understanding of the novel centrifugal microfluidic device designed for cell separation. The device includes multiple channels, contraction–expansion chambers, and reservoirs, all contributing to the effective separation of CTCs from other cells [4,21,107,108,109,110,111]. Supplementary Materials S4: Fabrication (S4): Various microfabrication techniques used in the study are presented in this document, with a focus on those successfully applied to the device. The techniques are selected based on the design requirements and critical parameters, such as the microchannel aspect ratio [112,113,114,115,116,117,118,119,120,121,122,123,124,125,126,127,128,129]. Supplementary Materials S5: Experiment/Test (S5): This document details the experiments conducted to separate target CTCs (MCF-7) from non-target cells (L929). It includes the cell culture process, the verification of antibody attachment to CTCs, and the evaluation of the device’s separation efficiency. The impact of different ratios of Iron Oxide Nanoparticles (IONPs) to arginine on the separation performance is also examined [53,55,56,130,131,132,133,134,135].
