# Supplementary material for: A Novel Size-Based Centrifugal Microfluidic Design to Enrich and Magnetically Isolate Circulating Tumor Cells from Blood Cells through Biocompatible Magnetite–Arginine Nanoparticles
Source: Sensors (Basel). 2024 Sep 18;24(18):6031. doi: 10.3390/s24186031 (PMC11436177; doi:10.3390/s24186031)
Supplement: Supplementary file 1 [file sensors-24-06031-s001.zip › sensors-3159403-supplementary.pdf]

Supplementary Material S1: Material Preparation (S1)

S1 The Process of CTC Magnetization

Magnetite nanoparticles, so-called Iron Oxide Nanoparticles (IONPs), have enjoyed clinical benefits for almost a hundred years. IONPs have established safety and significant clinical utility and versatility in cancer diagnosis, cancer hyperthermia therapy, iron deficiency anemia (IDA) [1], and cancer cell separation [2]. IONPs with diameters ranging from 1 nm to 100 nm were produced in various methods [3]. Several nonaqueous techniques, such as the reverse micelle [4], the solvothermal reaction [5], the high-temperature hydrolysis reaction [6], the interfacial coprecipitation [7], the organic colloid [8], and the thermal decomposition [9] methods, were invented to produce IONPs with uniform crystal size distribution successfully. In the current study, as a straightforward and effective aqueous chemical pathway to prepare IONPs [10], the coprecipitation method will be used to produce identical IONPs with homogeneous particle sizes ranging from 2 to 20 nm [11].

One technique to deliver the magnetic property to Circulating Tumor Cells (CTCs) is bonding the cell's antigen to the IONPs-antibody composite; however, direct binding between IONPs and antibodies is not possible due to the lack of surface functionalities (e.g., amine (NH_2) and carboxylic acid (COOH) functional groups that can participate in the acid-base reactions) in IONPs. Therefore, modifying IONPs is a crucial step in synthesizing IONPs-antibody composite. For this purpose, Arginine (Arg), as an α-amino acid used in the biosynthesis of proteins, was used [12]. Arg is an amphoteric compound due to the presence of both carboxyl and amine functional groups in its chemical structure. [13]. One of the major challenges of widely using IONPs in many applications is making IONPs stabilize against demolition during/after the synthesis process. As a result, several types of organic or inorganic coating agents, such as surfactants, amino acid, polymers, silica, or carbon, can be used to coat and protect IONPs from decomposition. Amino acid-coated IONPs, such as Arg-capped IONPs, can be an ideal candidate for many IONP–based applications [14-16].

Moreover, it was proved that Arg influenced the IONPs by controlling the particle size [17]. Also, saturation magnetization and electrostatic interactions of Arg-capped IONPs directly affect biological molecules (*e.g.,* proteins and nucleic acids), actively participating in several bio-application purposes. These characteristics can be accurately controlled by optimizing Arg concentration in the final composition [17, 18]. For example, regulating the amount of Arg on the IONPs surface makes it possible to accurately load the drug [19], control the amount of the molecules connected to antibodies or other biomolecules [20], control catalytic behavior [21],

and modify IONPs stability [18].

Combining Arg and IONPs leads to the formation of a shell-core binding between them, in which IONPs, as the core, are trapped within the Arg shell by a physical bond [22], and the carboxyl groups are placed outwards to be grifted by the amine group of the antibodies. Indeed, since Arg is featured by three aliphatic methylene groups capped by a positively charged guanidinium group [23, 24], the guanidino group ($HNC(NH_2)_2$) of Arg attaches onto the IONPs surface [20]. In many cases, an Arg-created protective shell assists in stabilizing IONPs, improves their biocompatibility, prevents them from aggregation, and offers an opportunity to engineer the surface properties for a variety of specific applications [25-27]. Overall, the purpose of such a binding is to attach Arg-capped IONPs to the antibody because this biocomposite will be capable of binding to the cell's antigen. In the following, since the antibody is also an amphoteric compound, there is a possibility of chemical binding between Arg and antibody through an acid-base reaction; however, their polar groups are generally not active enough to participate [28].

1-Ethyl-3-(3-dimethylaminopropyl) carbodiimide [EDC] is commonly used in conjunction with the N-hydroxysuccinimide [NHS] to stabilize large biomolecules and activate the carboxylic acid groups of Arg for acid-base reactions [29]. Activated carboxylic acids are esters with empty orbitals that can bind to the available amine groups in reaction media to form amides. The reason for using NHS is not only to modify the carboxylic acid group of Arg but also to strengthen and enhance the EDC characteristics in establishing the aforementioned acid-base chemical reaction [30]; in truth, NHS plays a booster role in synthesizing the biocomposite.

After employing EDC and NHS to modify Arg, the modified Arg-coated IONPs will chemically bond to the specific antibody. The biocomposite obtained at the end of this step is called MAENAb. The available antibody in the MAENAb biocomposite can bond to the antigen of CTCs through a lock and key model so that each lock can only be unlocked with one unique key [31]. Indeed, the body produces a specific antibody for each antigen; thus, the connection between antibody and antigen can be assumed to be analogous to a lock and key. The immune system uses the antigen-antibody interaction to identify foreign agents (foreign molecules or chemical toxins) in the body. In this research, the specificity of antibodies and antigens (or, better say, receptors on the surface of CTCs) are used to differentiate between target and non-target cells during magnetic separation. Moreover, the carboxyl and amine agents of antibodies are used to attach to cells and IONPs.

After such a connection, the magnetophoretic force can drive CTCs toward a magnetic source and separate them from other healthy blood cells. Increasing the binding energy between the precursors (IONPs, antibodies, and cancerous cell's antigen) will increase the yield of the reaction

(Figure S1.1), which, in turn, results in the efficient separation of magnetized CTCs under the applied magnetic field. Consequently, choosing the suitable type of antibody based on the type of CTCs and following the proper procedure for the combination of precursors plays an important role in the final result. The magnetization process of the cancerous cell's antigen is shown in Figure S1.1.

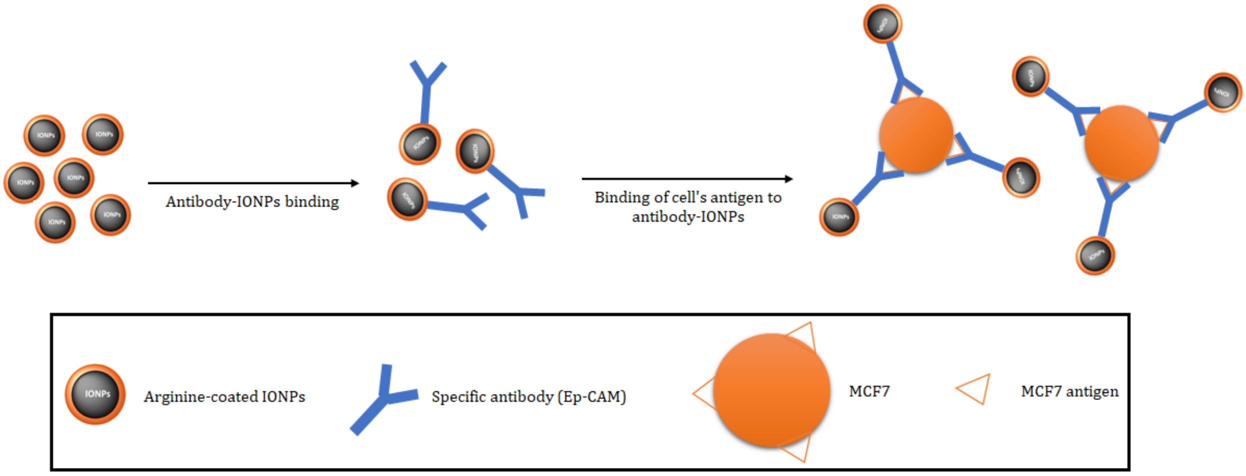

Figure S1.1: *Schematic of the magnetization process of MCF-7 (a kind of CTCs) using antibodies and magnetic nanoparticles.*

In this study, the EpCAM antibody, capable of covalent binding to breast cancer cell antigens (MCF-7), will be used because MCF-7 has been selected as the target cells for isolation [32]. Inspired by the literature and taking into account the specific circumstances of our experiment, the following synthesis strategy is proposed.

S1.1 Synthesis of IONPs

In brief, a 250-mL three-neck round-bottomed glass flask was equipped with a gas inlet/outlet, a condenser, and a mechanical stirrer. Two flasks were charged with ferrous chloride ($25\ mL;\ 0.2\ mol.L^{-1}$) and ferric chloride ($50\ mL;\ 0.2\ mol.L^{-1}$), separately. The weight of each content was achieved as $the\ required\ volume\ (mL) \times molarity\ (mole\ per\ liter\ or\ \mathrm{mol}/L) \times molecular\ weight\ (g/mol)$. The molecular weight (MW) of $FeCl_2$ and $FeCl_3$ are $126.8\ g/mol$ and $162.20\ g/mol$, respectively. As a result, 0.634 g of $FeCl_2$ and 1.622 g of $FeCl_3$ were weighed and poured into flasks (Figure S1.2a). Later, they were dissolved in enough millipore water, then placed the flasks on stirrers (Figure S1.2b).

After a while, the content of each flask was dissolved with more millipore water in 25- and 50-mL single neck round bottom flasks to reach the desired volume (Figure S1.2c). The content of both flasks was then poured into a single flask. The content of the flask was de-aerated by

bubbling highly pure argon gas (or nitrogen gas) for 20 min to make sure that no oxygen molecules were dissolved in the solution (because oxygen can cause oxide and demolish the product) (Figure S1.2d). Afterward, the flask was placed in an oil bath at 80 ± 3°C, and then NH_4OH (22 mL; 28-30 % (V/V) of ammonia) was added dropwise ($\frac{1}{3}\frac{ml}{min}$) into the flask under vigorous stirring (Figure S1.2e). The resultant black suspension was stirred at 80 ± 3°C for another hour and then allowed to cool to room temperature under stirring (Figure S1.2f). The formed black IONPs were subjected to magnetic separation and pulled to one side with a giant magnet, washed five times with water followed by ethanol until their pH became completely neutral (∼7) (pH was measured by Mettler Toledo's pH Meter[1]) (Figure S1.2g). The resulting solid material was separated from the solution and then dried via a vacuum oven (Figure S1.2h). At last, the dried IONPs were crushed and sealed to avoid aerial oxidation (Figure S1.2i) because oxidation in the air is one of the ways that transform Fe_3O_4 into γ-Fe_2O_3 [33].

[1] https://www.mt.com/ca/en/home.html

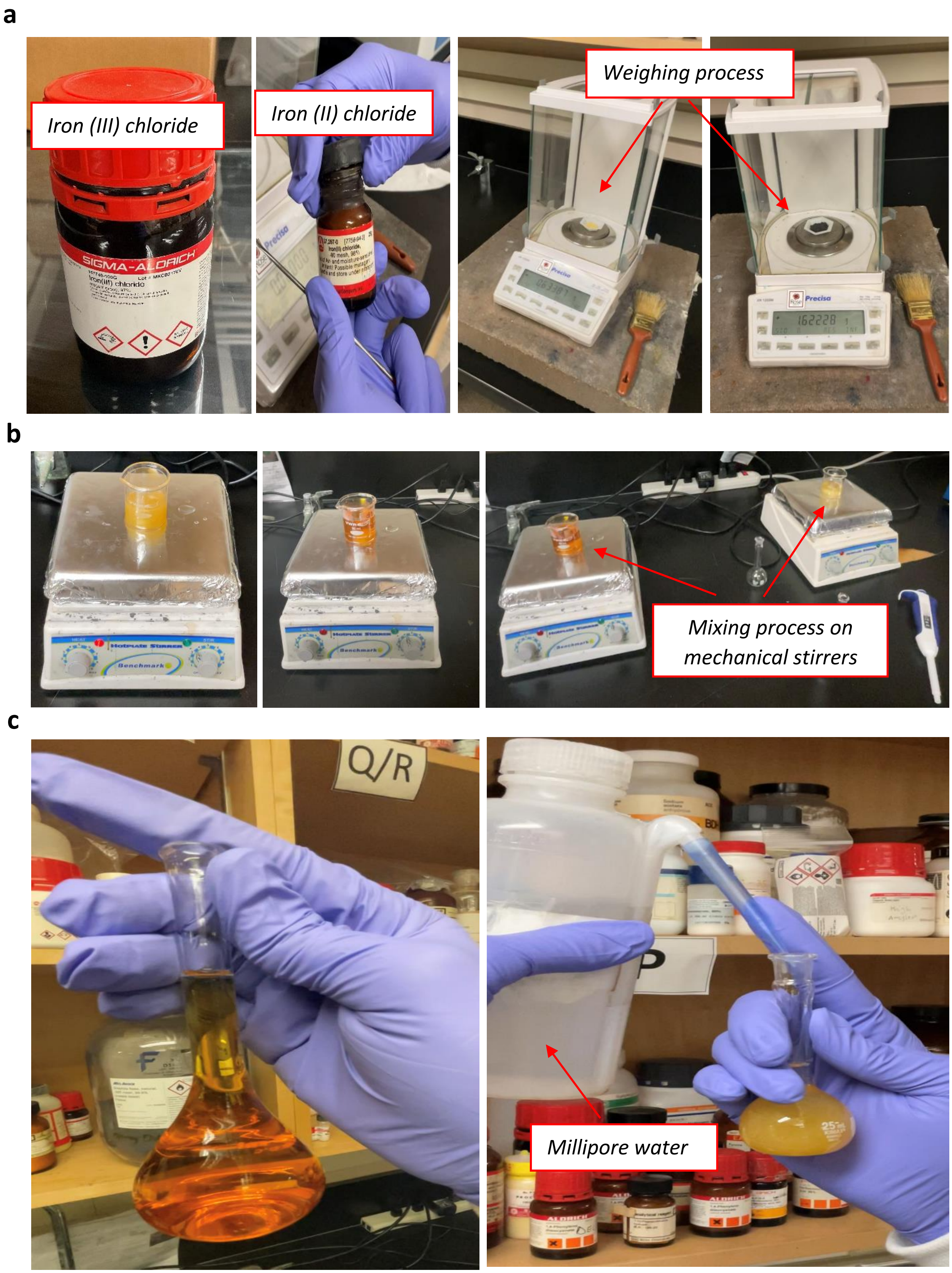
a
Iron (III) chloride
Iron (II) chloride
Weighing process
b
Mixing process on mechanical stirrers
c
Q/R
Millipore water

d

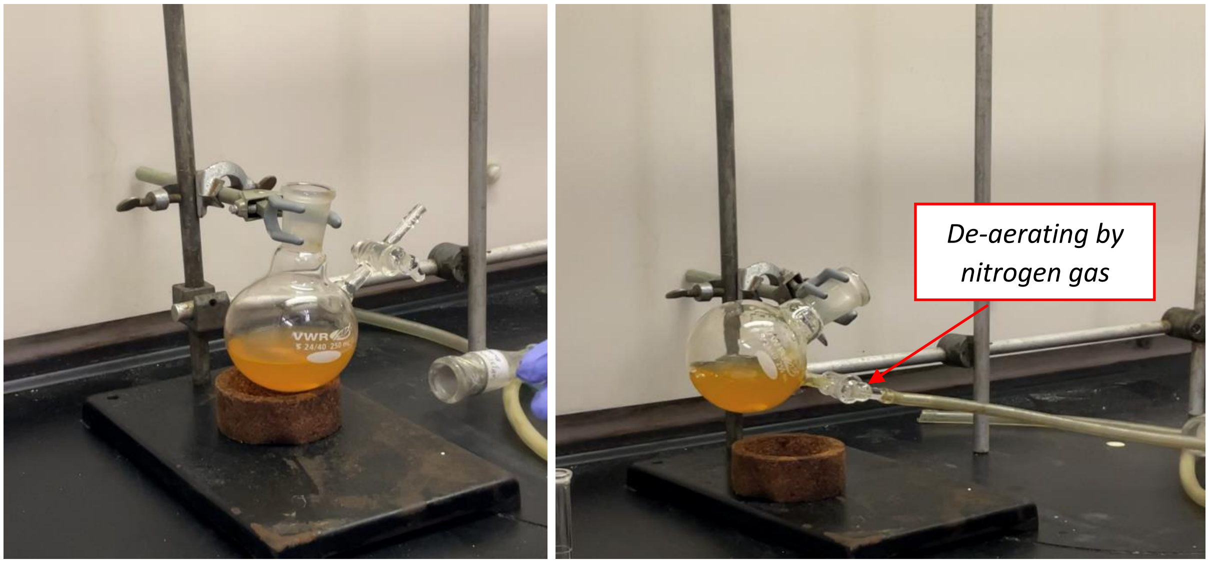
De-aerating by nitrogen gas

e

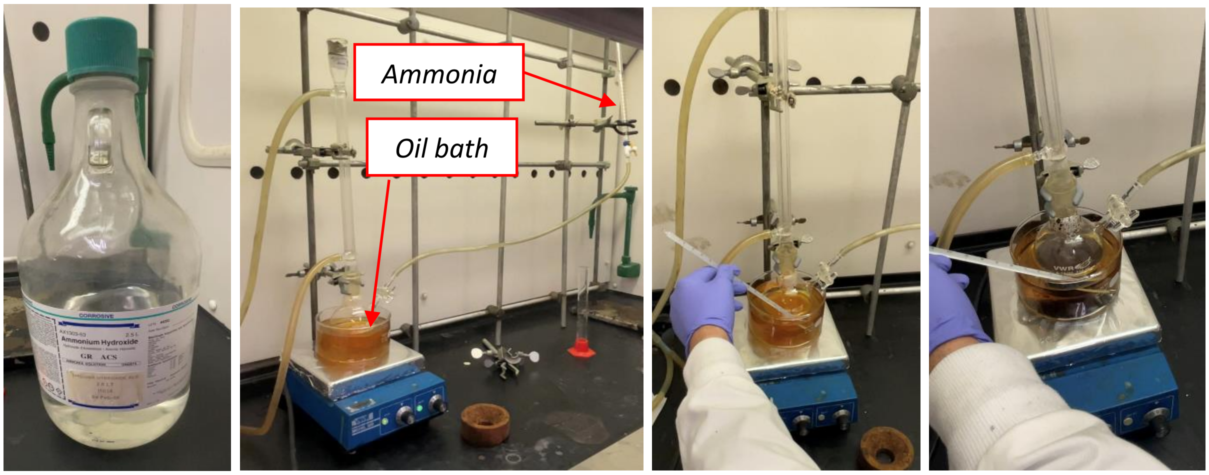
Ammonia
Oil bath

f

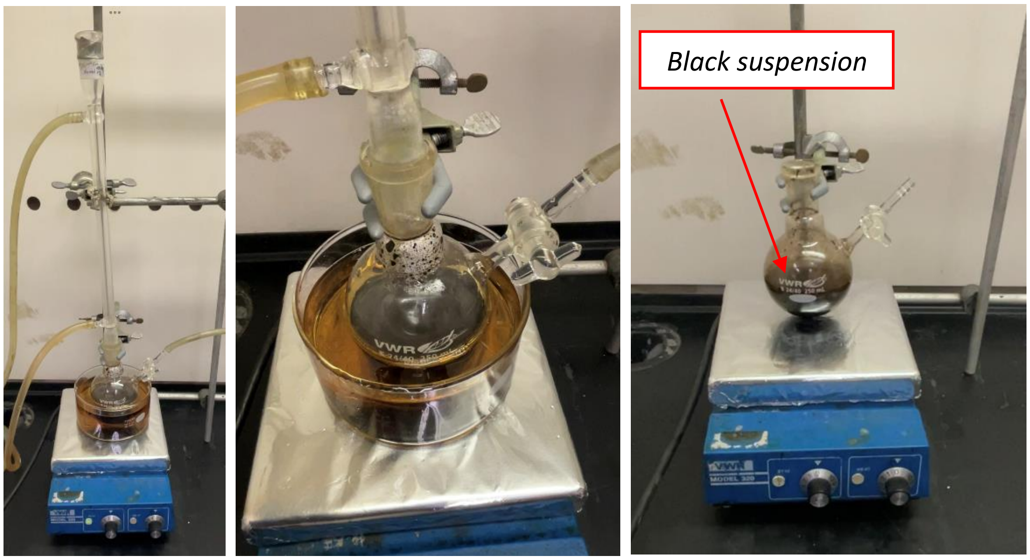
Black suspension

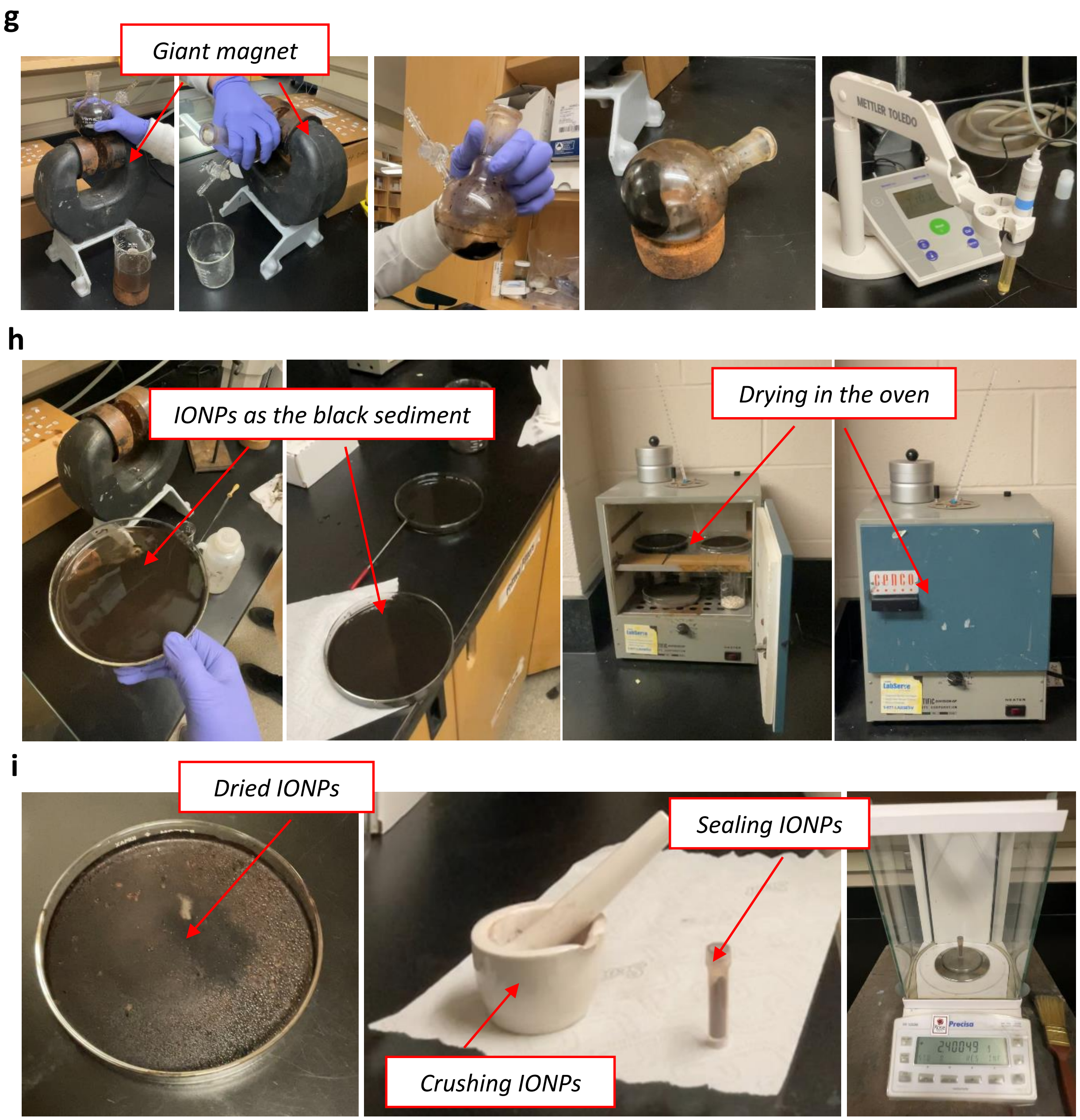

Figure S1.2: *The chemical process of synthesizing Iron Oxide Nanoparticles (IONPs);* ***a:*** *Iron (II) chloride (ferrous chloride), Iron (III) chloride (ferric chloride) and the weighing process;* ***b:*** *The mixture process of ferrous and ferric chlorides with water on mechanical stirrer;* ***c:*** *Reaching the desired volume by mixing with millipore water;* ***d:*** *De-aerating by bubbling highly pure argon gas (or nitrogen gas) into the flask;* ***e:*** *Adding ammonia dropwise into the flask in the oil bath under vigorous stirring;* ***f:*** *After stirring at 80 ± 3°C for one hour, the resultant black suspension was cooled to room temperature under stirring;* ***g:*** *Pulling the black sediment to one side with a giant magnet and are washed five times with water;* ***h:*** *The IONPs obtained from the solution was left in the oven to be dried;* ***i:*** *The dried IONPs was crushed and sealed.*

X-ray diffraction (XRD) is conducted to characterize these two samples and examine the results at the first step of the experiments (synthesis of IONPs)[1]. As a non-destructive technique, XRD provides detailed information about the crystalline structure and phase purity of the material [34]. In this study, the crystal structure of IONPs was determined and confirmed using XRD. In this experimental method, XRD patterns for the powder XRD studies were recorded using a Rigaku Ultima IV X-Ray diffractometer equipped with a graphite monochromatized Cu source ($K\alpha 1$: 1.54060 Å and $K\alpha 2$: 1.54443 Å), Cross-Beam Optics (CBO), and a Scintillation Counter detector (Figure S1.3). The measurements were taken with the Multipurpose Attachment using the para focus method. The $K\beta$ diffraction components were removed by using a Ni foils filter. 2θ was checked with the Si powder sample (2 2 0) plane at 47.28° and scanned in the range from 10° to 90° with a step size of 0.02° and a scan rate of 2° per minute for all samples (an exposure time of 0.6 seconds.step^{-1}).

[1] All XRD data were collected at the Saskatchewan Structural Sciences Center (SSSC) at the University of Saskatchewan.

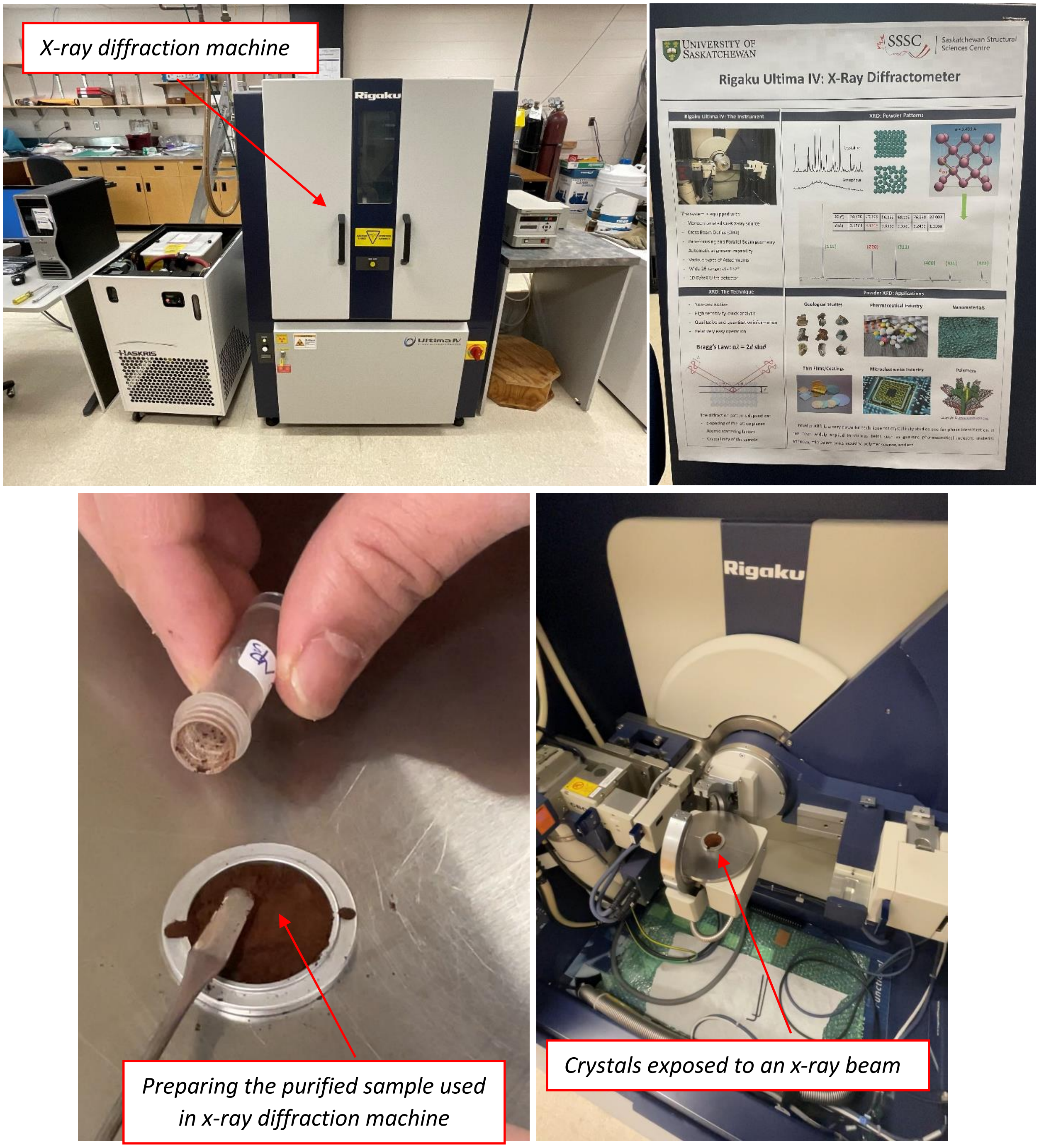

Figure S1.3: *X-Ray diffractometer and the process to make the sample ready for XRD.*

Figure S1.4 depicts the XRD pattern of IONPs synthesized based on the coprecipitation method [35]. IONPs displayed a pattern that includes diffraction peaks corresponding to planes of a face-centered cubic (fcc) crystal structure of Fe_3O_4 [36]. The sharp and intense peaks indicated the sample's good crystallinity. The peak intensity ratio could also be assessed to calculate the molar ratio of ferrous to ferric in iron oxides [37]. The obtained XRD pattern in Figure S1.4 matches well

with standard magnetite reflections (JCPDS file No. 19-0629) and is in good agreement with literature results [18, 38], which means that there is no evidence of impurities seen.

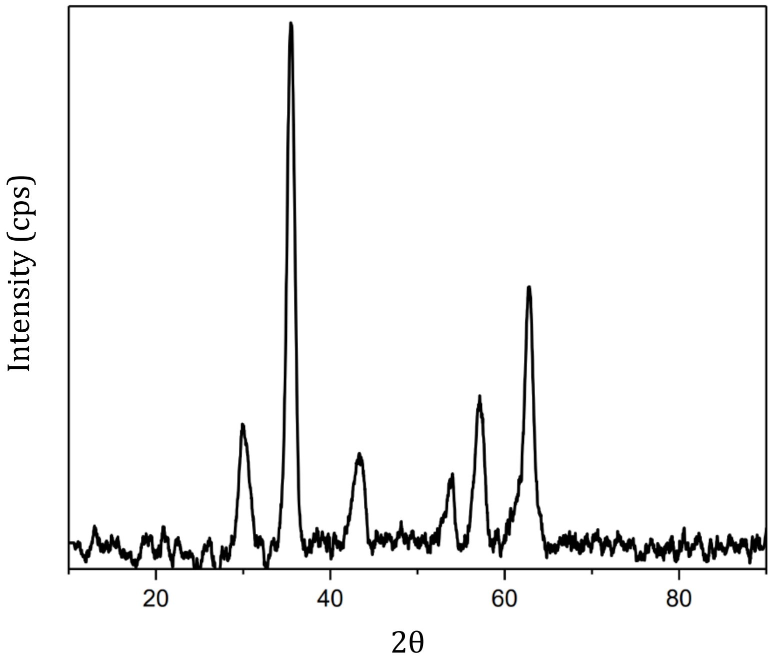

Figure S1.4: *XRD pattern of synthesized IONPs in the 2ϑ range of 10-90°.*

Furthermore, the analyzed XRD pattern of IONPs is shown in Table S1.1. The results indicate that the prepared IONPs have an approximately uniform size distribution and pure magnetite phase. The crystalline size (D) of IONPS is less than 20 nm, with a standard deviation of ± 2.69 nm. Nevertheless, it should be mentioned that such information does not play a critical role in the current study.

The crystalline size of IONPS is calculated based on Debye–Scherrer equation [39]:

$$D = \frac{K\lambda}{\beta Cos(\theta)} \quad (1)$$

Where *D* is the crystalline size of IONPS, *K* indicates the Scherrer constant (Scherrer constant denotes the shape of the particle and its value is most commonly taken as 0.9), λ represents the wavelength of the X-ray beam, *β* denotes the full width at half maximum (FWHM) of the related diffraction, and θ is the Bragg angle [40].

β is calculated based on Equation (2):

$$\beta = \beta_1 - \varepsilon \quad (2)$$

Where the microstrain effect (ε) and β_1 are calculated through Equation (3) and Equation (4):

$$\varepsilon = \frac{\beta_{measured}}{4\tan(\theta)} \quad (3)$$

$$\beta_1 = \sqrt{\beta^2{}_{measured} - \beta^2{}_{instrumental}} \quad (4)$$

In the above equation, the instrumental broadening effect ($\beta_{instrumental}$) is calculated based on Equation (5):

$$\beta_{instrumental} = 0.00558\, tan^2(\theta) - 0.00901\, tan(\theta) + 0.01039 \quad (5)$$

Table S1.1: *The analyzed XRD pattern of IONPs based on Figure S1.4, Bragg, and Debby-Scherrer equations. The standard deviation of IONPs crystalline size equals 2.69 nm.*

**2θ** (°)	**(h k l)**	**d** (Å)	**a** (Å)	**D (nm)**
**30.12**	(2 2 0)	2.96	8.38	9.53
**35.47**	(3 1 1)	2.53	8.38	14.11
**43.27**	(4 0 0)	2.10	8.35	10.38
**53.77**	(4 2 2)	1.70	8.34	17.27
**57.10**	(5 1 1)	1.61	8.37	12.09
**62.77**	(4 4 0)	1.48	8.36	15.15

d (Å) = Interplanar spacing   a (Å) = Lattice constant   D (nm) = Crystalline size

S1.2 Coating IONPs with Arg

After this step, to compare and explore the effect of the IONPs/Arg ratio, five kinds of samples with different combination ratios of IONPs to Arg (1:1, 1:3, 3:1, 1:20, and 1:50) were prepared (Figure S1.5a). It is worth mentioning that the brownish degree of each sample can be used to distinguish them from each other. These different colors can also be interpreted as evidence of attaching Arg molecules to the surface of IONPs and their ability to modify the characteristics of IONPs. It was reported that the existence of Arg molecules on nanoparticle surfaces might facilitate nucleation and hinder the growth of the magnetite nanoparticles [41]. Hence, the size of magnetite particles can reach a smaller size at higher Arg concentrations, which, in turn, will be a significant parameter in the formation of IONPs and cell separation investigations under a magnetic field [17]. It was also indicated that the presence and concentration of Arg would be a

decisive factor in the preparation and formation of IONPs [17]. Although it has been claimed that increasing the coating agent ratio reduces the magnetization of IONPs [42], it does not generally affect their magnetic properties and still allows them to be super-paramagnets [18]. In our case, it is especially expected that the increased Arg ratio provides more opportunities for IONPs to bond with an antibody. Examining the cellular viability with different concentration ratios of IONPs/Arg demonstrated the biocompatibility of Arg-capped IONPs for biomedical applications [17]. Therefore, these composites have the strong potential to be utilized in biomedical imaging, diagnostics, and therapeutics because of their suitable biocompatibility, ideal colloidal stability, high saturation magnetization, and surface-bound amino groups. The importance of these ratios in cell separation will be thoroughly investigated in Section 6: Results and Discussion.

For example, 60 mg of IONPs should be combined with 45.12 mg of Arg (as a coating agent) to reach a 1:1 ratio, since the molar mass of IONPs and Arg are regarded as 231.53 g/mol and 174.20 g/mol, respectively. After weighing the required amount of each reagent, Arg was dissolved in 50 mL of millipore water (which were de-aerated under a nitrogen atmosphere for 30 min to make sure that no oxygen molecules were dissolved in the water to do the oxidation reaction with IONPs) and stirred for 5 min. Later, the solution temperature was increased to around 80°C, IONPs were then added to the solution in the beaker, and the whole solution was continuously stirred for 3 h at the same temperature (millipore water could be added in case of necessity). It was found that the formation of magnetite nanoparticles is independent of the reaction time [17, 43]. Indeed, the size of IONPs does not change by increasing or decreasing the reaction time.

Later, the sample was washed 3 times with millipore water to remove residual Arg and solvent. After each washing step, IONPs were subjected to the magnet and separated from the suspension. Finally, it was left in the oven to be dried for 12 h at 60°C. Figure S1.5b depicts the weighing, mixing, heating, stirring, and drying procedure to prepare a 1:1 ratio of IONPs to Arg from beginning to end. The other four samples (i.e., for 1:3, 3:1, 1:20, and 1:50 ratios) were produced and treated in the same manner described above. After drying the prepared samples with different ratios of IONPs to Arg, they were crushed and sealed to avoid aerial oxidation (Figure S1.5c).

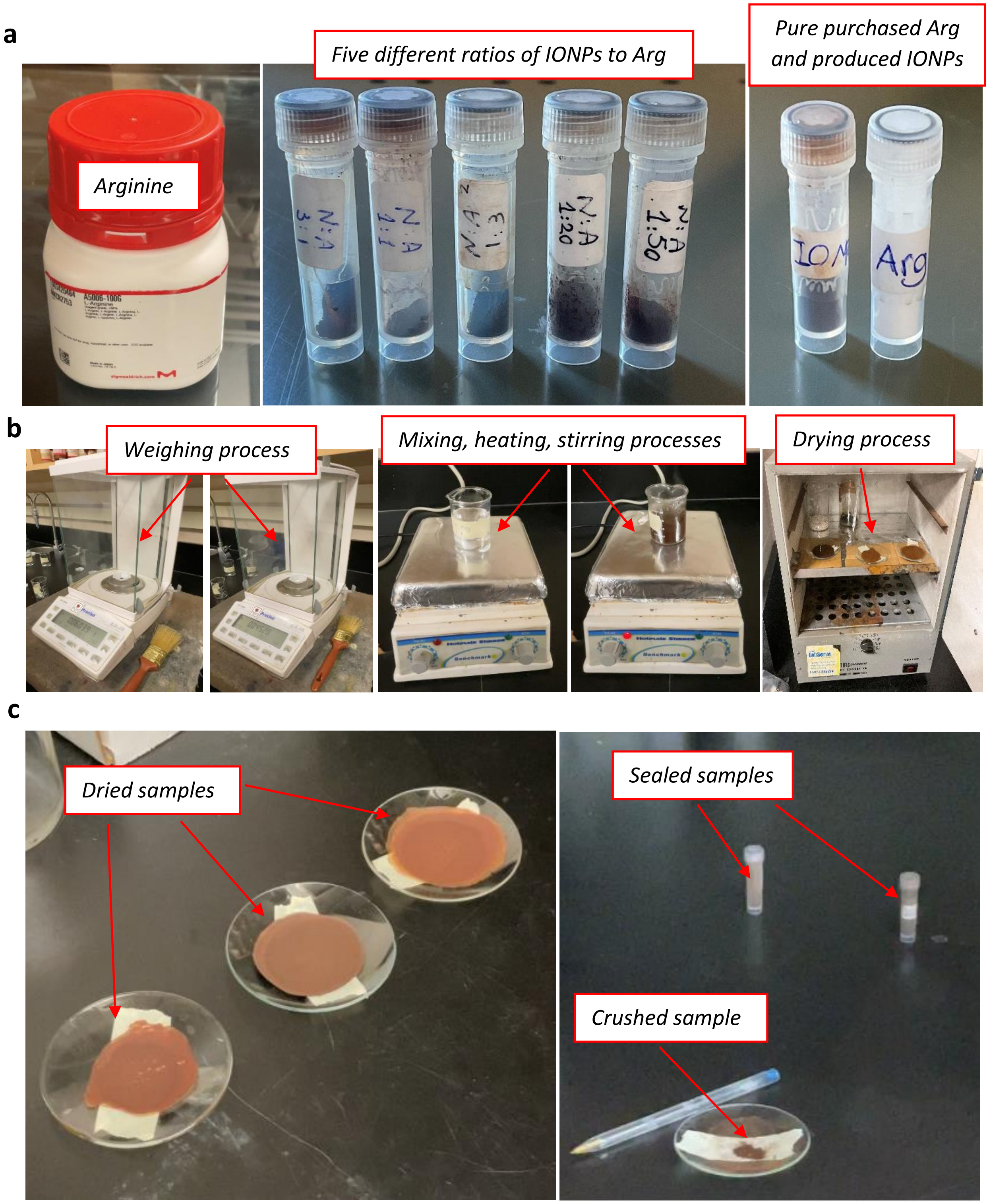

Figure S1.5: *The process of making different ratios of IONPs to Arg;* ***a:*** *Five different ratios of IONPs to Arg along with the pure purchased Arg and obtained IONPs;* ***b:*** *Weighing, mixing, heating, stirring, and drying processes for preparing a 1:1 ratio of IONPs to Arg;* ***c:*** *Crushing, and sealing the 1:1 ratio sample for investigation by Raman Spectroscopy.*

Later, a Raman spectrometer was used to investigate if IONPs are well coated with Arg. In this spectrometer, a high-power laser exposes to the sample, and according to the change in polarizability of molecular bonds in the sample, the chemical structure and composition of materials are identified[1]. Indeed, Raman spectroscopy measures the relative frequencies at which a sample scatters radiation and enables multiple times analysis without damage to the sample. In this study, Raman spectroscopy measurements were operated at 514 nm laser excitation (due to exhibiting a good band resolution under this excitation) carried out on a Renishaw Raman spectrometer (inVia™ confocal Raman microscope[2]) attached to a Leica optical microscope with a 20× objective lens and the backscattered Raman signals were collected with a Peltier cooled CCD detector (Figure S1.6a).

The spectra were recorded over a range of 50–2000 cm^{-1} range with 10 s exposure to 10% laser power (~1.5 mW for 514 nm laser power) in extended modes. In addition, before starting with samples, the instrument calibration was verified using an internal Si (110) sample, measured at 520 cm^{-1}. The results of Raman data for the sample with the 1:1 ratio of IONPs to Arg, pure Arg, and bare IONPs are shown in Figure S1.6b. This comparison illustrates the differences in spectral intensity between the samples. As shown in this figure, the comparison between Arg, IONPs, and the composition sample (with the 1:1 (IONPs to Arg) ratio) curves conveys the attachment between these two substances. In other words, the effect of the existence of Arg molecules on the surfaces of IONPs is revealed by investigating the fluctuation of the composition curve. For example, between the range of 1500-2000 cm^{-1}, the dimple in the composition curve came from the downward movement of IONPs. The other peaks and troughs could also be interpreted in the same way.

[1] The Raman spectroscopic imaging for this research was carried out at the Saskatchewan Structural Sciences Center (SSSC) at the University of Saskatchewan.

[2] https://www.renishaw.com/en/invia-confocal-raman-microscope--6260

a

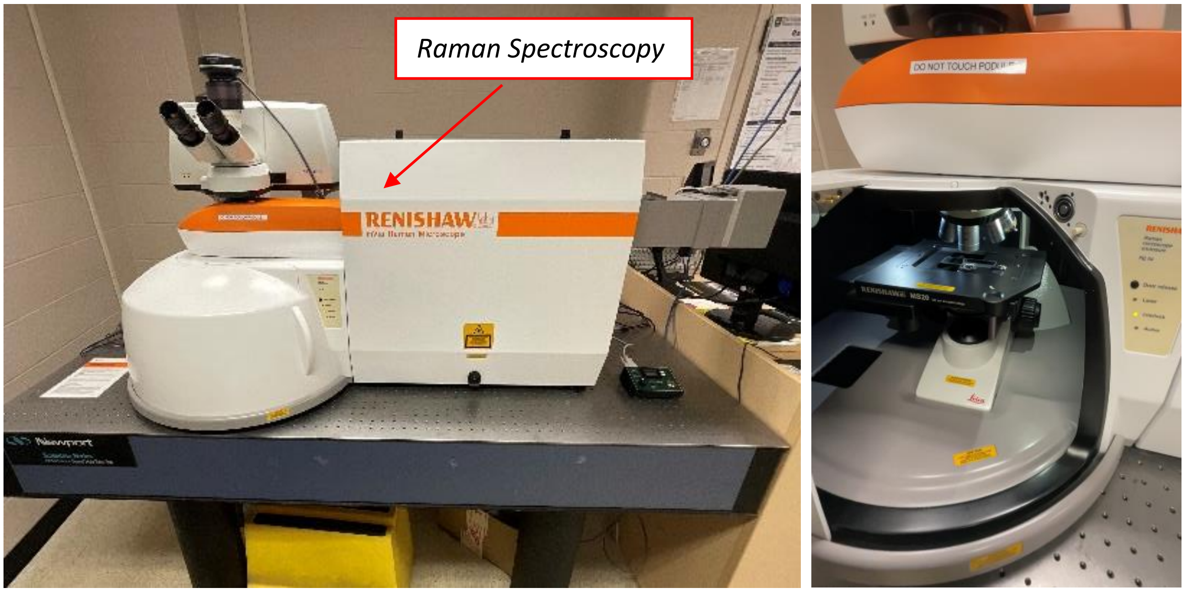

b

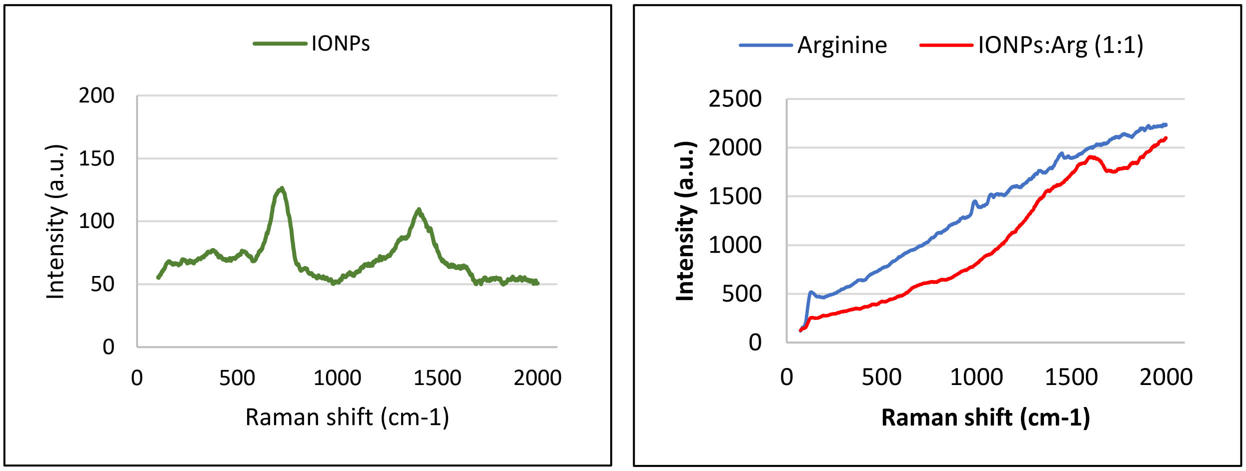

Figure S1.6: *A method to investigate the coating of IONPs with Arg;* ***a:*** *The Renishaw Raman spectrometer;* ***b:*** *Raman spectroscopy analysis of Arg, IONPs and their Arg-capped IONPs composite with the ratio of 1:1 under 514 nm laser excitation.*

To further analysis of the Arg attachment, thermograms of the compounds were also obtained by means of Thermogravimetric analysis (TGA) (Q500 TA Instruments[1])[2]. As a thermal analytical

[1] https://www.tainstruments.com/

[2] All experiments related to the thermogravimetric analysis were performed in the chemistry department at the University of Saskatchewan.

technique, TGA measures the weight of a sample when its temperature changes over time. Valuable information can be comprehended by such measurement and its further analysis. This information covers the topics of physical processes, including phase transitions accompanied by weight loss, absorption, desorption, adsorption, and chemical phenomena, including thermal decomposition, chemisorption, and solid-gas interactions (*e.g.,* oxidation or reduction, if possible) [44]. In this present analysis, TGA was used to investigate the stability of the composition of Arg-coated IONPs by studying the weight loss profiles versus temperature changes. The experiment started with heating the samples in open aluminum pans at 25°C and allowing them to equilibrate for 5 min before heating at a scan rate of 5°C.min^{-1} up to 500°C with a 10.0 mL.min^{-1} balanced purge flow.

Figure S1.7a-b shows the TGA and first derivative TGA (dTG) thermograms of Arg, IONPs, and their composite. In the current test, a desirable diagram is generated based on the information of temperature and the first derivative of weight loss (Figure S1.7b); however, the weight loss data was also used in the company with temperature variations to create the TGA curves to provide a clear view of the experiment purpose (Figure S1.7a). As can be realized from Figure S1.7a-b, Arg appeared to have a sharp thermal event between 230 and 270 ˚C, while only ~1% of IONPs decomposed before 500 ˚C. This valuable conclusion conveys that IONPs can successfully survive in temperatures below 500 ˚C without losing huge weight or causing significant decomposition. As a result, one can conclude that whatever happens to the composition of these two materials in a temperature range of 0-500 ˚C relates to the existence of Arg in that composition. In other words, after composting IONPs with Arg, the same thermal stage assigned to the degradation of Arg was seen in the TGA and dTG of Arg-capped IONPs (*e.g.,* 1:1 ratio) (red color). This trough (in Figure S1.8a) or peak (in Figure S1.7b) provides evidence for the successful grifting of IONPs with Arg. Further experiments show that the percentage of weight loss of the samples formed at high Arg concentrations (*e.g.,* 1:20 and 1:50) is more than that prepared at low concentrations (*e.g.,* 1:1 and 1:3), which is in accordance with literature results [17, 45]. This is true because the formed IONPs with smaller sizes have a larger surface area that can adsorb more Arg on their surface, resulting in a greater weight loss percentage [45].

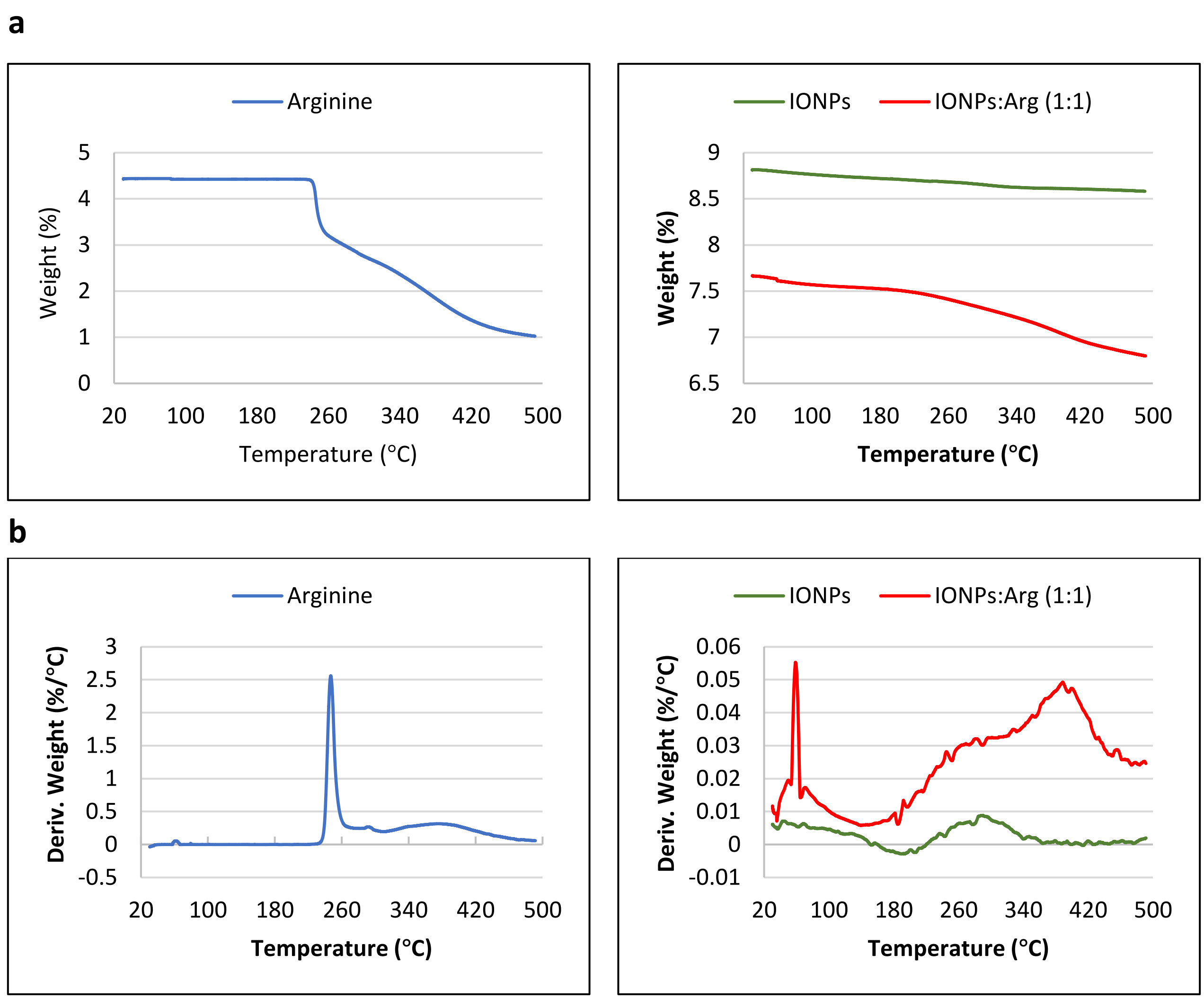

Figure S1.7: *A method to investigate the coating of IONPs with Arg;* ***a:*** *TGA of arginine, IONPs and their Arg-capped IONPs composite with the ratio of 1:1;* ***b:*** *First derivative TGA (dTG) of arginine, IONPs and their Arg-capped IONPs composite with the ratio of 1:1.*

S1.3 Synthesis of MAENAb biocomposite

To activate the carboxylic acid groups in Arg, 2 mg of Arg-coated IONPs (MA) was added to 10 ml phosphate-buffered saline (PBS) solution (pH~7.4) (Figure S1.8a) and placed in an ultrasonic bath while controlling the temperature in a range of 0-8°C. After 1 h, 0.442 g of EDC was poured into the buffer mixture (MAE) (Figure S1.8b). Then, 0.053 g of NHS was poured into the buffer solution (the product is called MAEN at this step) (Figure S1.8c) and allowed to homogenize well for 1 h ultrasonically (Figure S1.8d). Since EDC and NHS must be kept at a low temperature, sufficient crushed ice was added to the beaker to keep the temperature as low as possible. Furthermore, the water temperature was regularly monitored to replenish the ice as it was melting. The buffer

solution was diluted with 10 ml of PBS, and 50 µl of antibody was added to the mixture (Figure S1.8e). The mixture was stored for 24 h at a temperature below 4°C while stirring magnetically (Figure S1.8f). Finally, antibodies bonded to the nanoparticles were separated by subjecting them to a strong magnet while evacuating the phosphate buffer content. Then, the isolated biocomposite product (MAENAb) was washed thrice with PBS solution and stored in 1 ml of PBS solution below 4°C (Figure S1.8g).

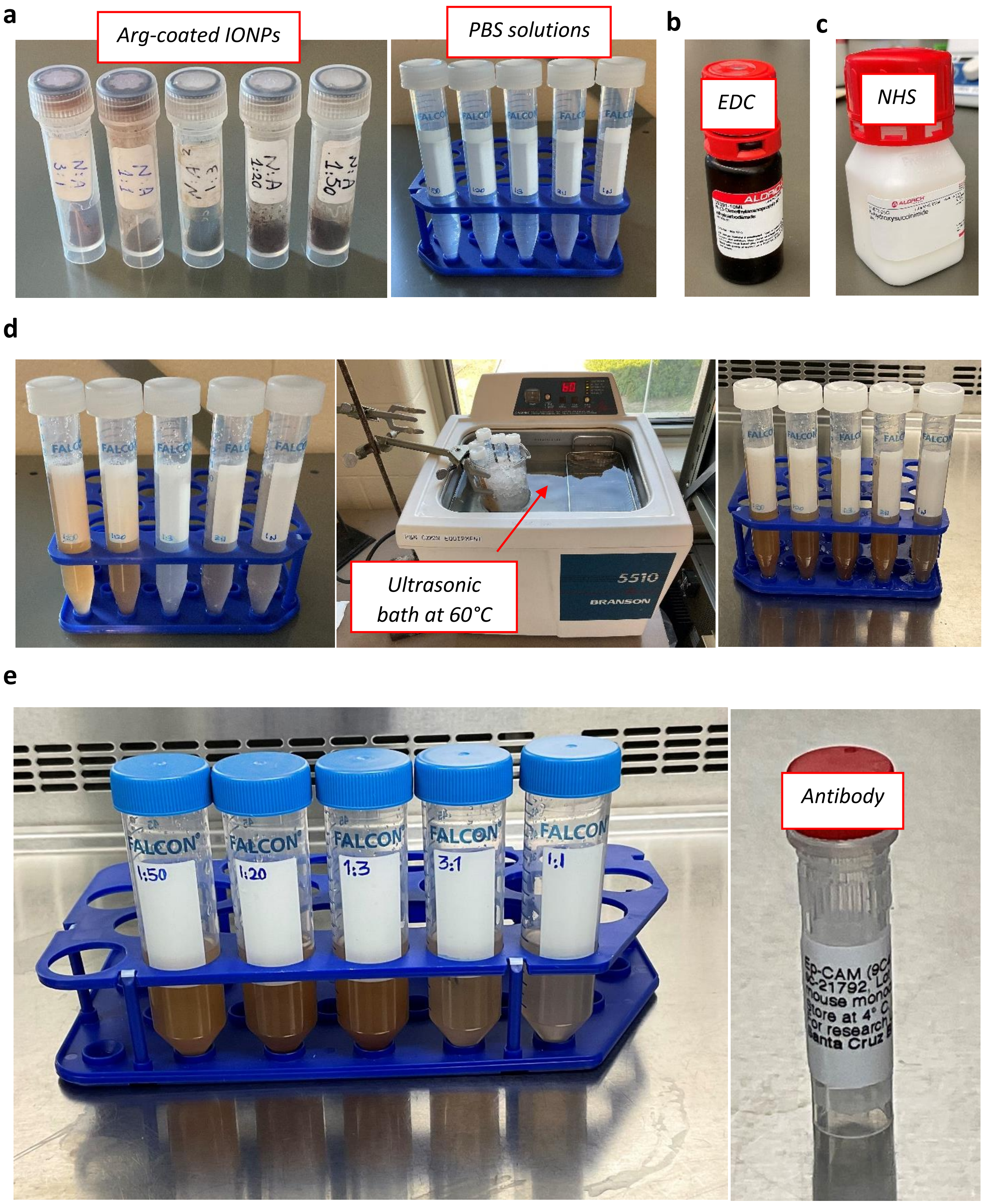
a
Arg-coated IONPs
PBS solutions
b
EDC
c
NHS
d
Ultrasonic
bath at 60°C
5510
BRANSON
e
FALCON
1:50
1:20
1:3
3:1
1:1
Antibody

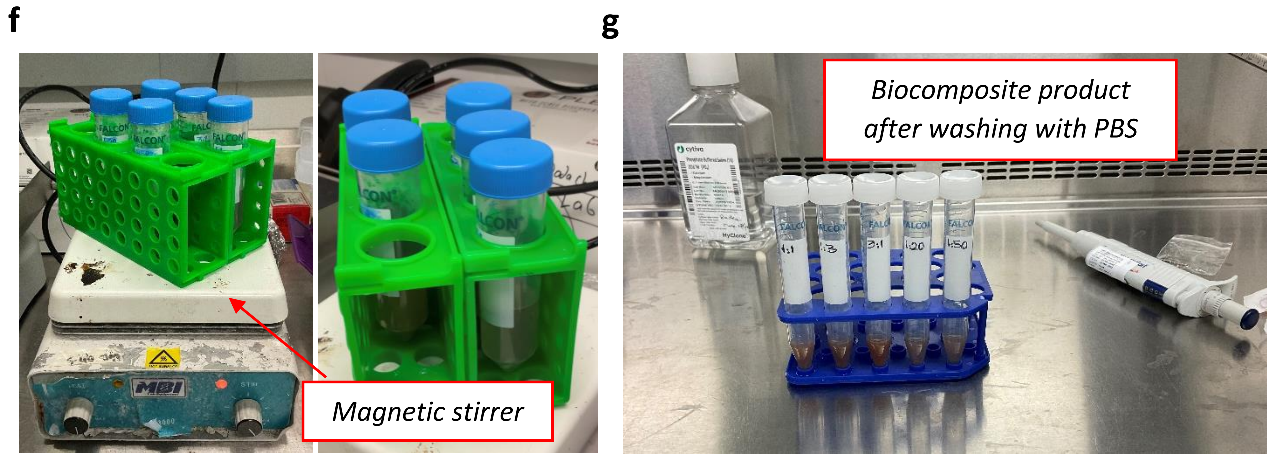

Figure S1.8: *The process of synthesizing MAENAb biocomposite;* ***a:*** *Adding Arg-coated IONPs (MA) to phosphate-buffered saline (PBS) solution;* ***b:*** *EDC purchased from Sigma-Aldrich was added to the buffer mixture (MAE);* ***c:*** *NHS purchased from Sigma-Aldrich was added to the buffer solution (MAEN);* ***d:*** *Homogenizing the solution in an ultrasonic bath at 60°C for 1 h;* ***e:*** *Diluting the buffer solution with 10 ml more of PBS and adding the antibody;* ***f:*** *Storing the solution at a temperature below 4°C on a magnetic stirrer for 24 h;* ***g:*** *Washing the biocomposite product (MAENAb) 3 times with PBS solution.*

S1.4 Exclusive antibody-antigen binding

The MAENAb biocomposite mixture produced in Section S.1.3 was then mixed with the CTC's antigen (which needed to be prepared, multiplied, and cultured) through so-called "exclusive antibody-antigen binding" [46]. This chemical reaction resulted in magnetized CTCs. This step started with mixing 1 million MCF-7 cells (refer to Section 5: Experiment) in 1 ml of PBS solution at the harvesting step (refer to cell culture) (Figure S1.9a). According to the experimental studies, MAENAb must be mixed with MCF-7 in a 1:5 V/V ratio [47] and placed in an incubator for 1 h while keeping the temperature constant at 37°C. This step resulted in exclusive antibody-antigen binding. Figure S1.9b shows a schematic procedure for supplying magnetic properties to MCF-7 breast cancer cells.

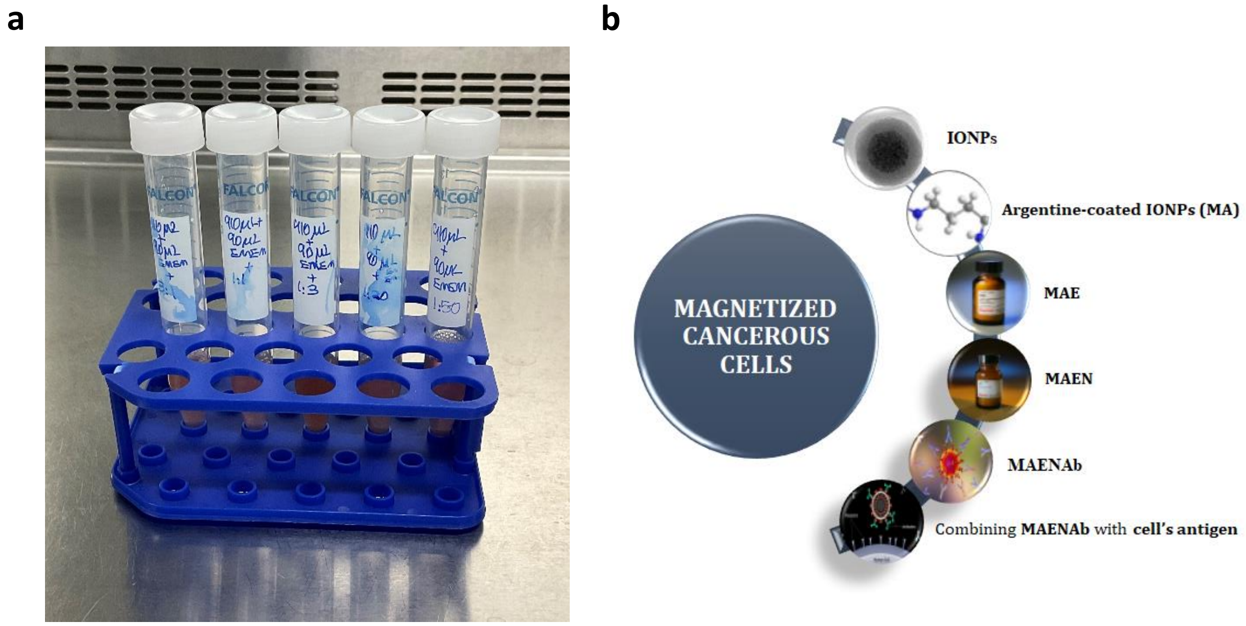

Figure S1.9: *Mixing MAENAb biocomposite with CTCs via an exclusive antibody-antigen binding;* ***a:*** *Mixing 1 million cultured MCF-7 cells in PBS solution with a specific ratio;* ***b:*** *The whole process of cell magnetization at a glance as proposed by the author.*

Supplementary Material S2: Rotating Device (S2)

S2 Rotating Device Design

The driving force for fluid flow inside microfluidic centrifugal systems is the volumetric force arising from the translational and rotational accelerations. Therefore, an external machine with the ability to spin the compact disk platform in two different rotational directions must be employed to control the angular speed. The pulsatile inertial microfluidic system is usually controlled by shutting on and off the fluid flow through an external pump, which requires the interruption of the process. In this study, a centrifugal switch motor with double-sided angular velocity control was designed, assembled, and tested for the first time at the mechanical laboratory of the University of Saskatchewan (Figure S2.1a).

A photo laser speedometer, known as a tachometer, was used to control and measure the engine rotational speed (Figure S2.1b). Tachometers work using a flash that can be stimulated at different frequencies to measure the rotation speed of a shaft or disk. When the flashing frequency of the tachometer is equal to the rotational frequency of the rotating object, the rotation frequency can be obtained and represented by Revolutions Per Minute (RPM). This rotational velocity can be converted to the input voltage of the motor, which is shown on a voltage indicator. As a result, the rotational speed of the motor can be set via the input voltage instead of a tachometer. To achieve this purpose, the input voltage to the motor is determined for different rotational velocities. The rotational speed of the motor is then adjusted through the obtained voltage to perform the tests and experiments.

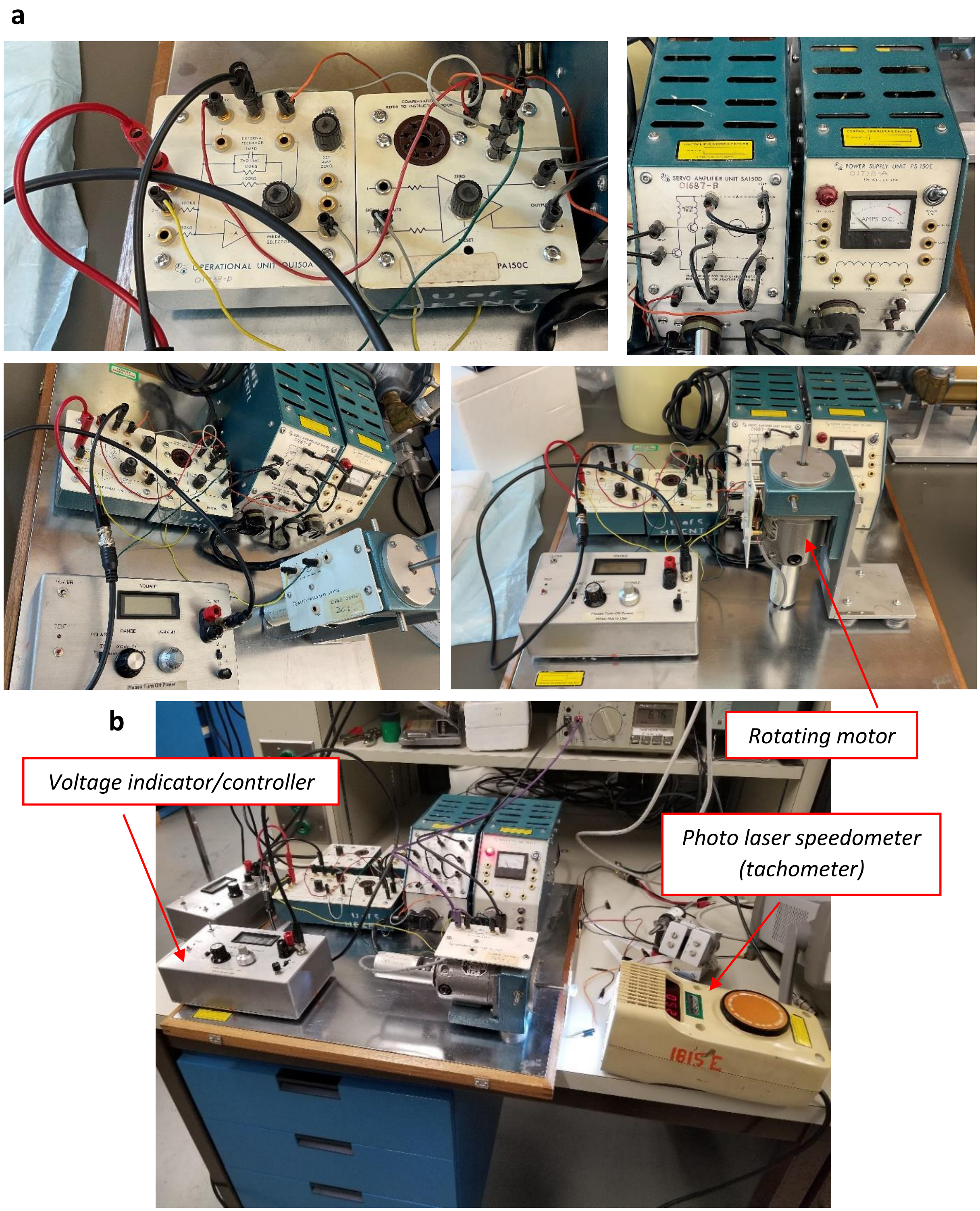

Figure S2.1: *An overview of the rotating motor along with the voltage controller;* ***a:*** *The centrifugal switch motor with double-sided angular velocity control;* ***b:*** *Measuring the engine angular velocity by the photo laser speedometer (tachometer).*

Supplementary Material S3: Detailed Design (S3)

S3 Detailed Design

The novel centrifugal microfluidic device designed, as shown in Figure S3.1, consists of two angled channels with multiple contraction-expansion chambers, three outlets connected to three reservoirs, and an inlet. The channel inlet connects to an inclined main channel on a centrifugal platform with multiple contraction-expansion chambers (see **a** in Figure S3.1), where the blood sample containing two types of cells will be injected. The main microchannel is connected to a bifurcation region at the end that is extended to two outlets:

One is connected to Reservoir #1 for non-target cells, which are separated due to the inertial hydrodynamic forces, bifurcation law, and available forces on a rotational platform such as centrifugal, Coriolis, Euler forces, and secondary vortices (i.e., Dean vortices) perpendicular to the main flow stream which cause Dean drag force on the curved channel. Centrifugal force drives the particles inside the inlet chamber into the microchannel with multiple contraction-expansion arrays (CEA) (see **b** in Figure S3.1). These particles will then be positioned on their equilibrium paths after force balancing. Lee *et al*. demonstrated that the particles within a CEA microchannel on a stationary platform could be effectively sorted based on their size through balancing lift and Dean drag forces [48]. Centrifugal force (as a size-dependent force) can also be used on a rotating platform to separate particles of different sizes. At the end of this part of the microchannel, the fluid flow is split into two branches with unequal flow rates to use the maximum potentiality of bifurcation phenomena for cell separation (see **c** in Figure S3.1). This bifurcation region was designed and optimized by numerical simulations in such a way as to maximize the number of target cells entering the second part of this device. Whereas it tries to conduct the majority of the non-target cells towards the high-flow-rate branch (i.e., the microchannel upper side (Reservoir #1)) under the effect of inertial forces and bifurcation law. Therefore, cells of different sizes can be partially separated in the first part of this device.

Second is connected to the device's hybrid section, which will take advantage of inertial and centrifugal forces as well as magnetophoretic separation techniques to increase the final cell separation efficiency. The main difference between the first and second parts of this device is that the bifurcation region is superseded by a magnetic region at the end of the microchannel (see **d** in Figure S3.1). This hybrid section consists of three parts:

1. Reservoir #2 for non-target cells separated due to the inertial forces and available forces on a rotational platform, such as centrifugal, Coriolis, Euler forces, and Dean drag force on the curved channel (see the explanation for the first part). Two cells with different sizes and densities within a curved microchannel experience different amount of centrifugal

force, which sort them into two different streamlines. Also, the channel curvature in both parts of the device plays a key role in cell separation because such curved channels boost the formed Dean vortices and enhance the final separation. Dean flow, a rotational flow that is normal to the main flow direction, can make the cells move transversely in these curved channels. The difference in the distance of these cells from the center of each formed Dean flow results in a change in the centrifugal, inertial and drag forces and increases the distance between the particles;

2. An inclined main microchannel with multiple contraction-expansion chambers (see **a** in Figure S3.1). It should be noted that the intended incline in the flow direction will change the magnitude of applied centrifugal force on each cell. Because the amount of force, which applies to cells on a rotary platform, is highly dependent on the location of cells with respect to the center of rotation (distance between the rotation center and the cell);
3. Reservoir #3 considered near the permanent magnets for separating target magnetized Circulating Tumor Cell (CTCs) due to the inertial forces, forces on the rotational platform, and magnetic force. Indeed, the magnetically labeled CTC will deviate under the permanent magnets towards Reservoir #3, which is considered for the target cells. It is noted that the interaction of magnetic force with CTCs is indirect, which, in turn, does not endanger the viability of CTCs. Indeed, CTCs are attached to magnetic nanoparticles through antigen-antibody binding complexes. Consequently, the physical and biological properties of the target CTCs are both involved in the separation process, which finally results in more separation efficiency.

The proposed device in Figure S3.1 is capable of performing the pumping, sorting, and cell-separating functions. The pumping function can be accomplished via injecting the blood sample into Reservoir #1 and aspirating it out of the outlet chambers while the microfluidic device recirculates at constant rotational speed in two opposite directions. Spinning the device, which is mounted on a motor, provides the driving forces to flow the sample within the main channel and separate differently sized particles due to their inherent physical and acquired magnetic properties. Changing the direction of rotation not only can be used for separation purposes (or mixing purposes for future functions) but also can reduce the length of the final design; in this case, the location of reservoirs, magnets, and the slope of the main channel should be carefully noted. The duration of rotation in each direction will be estimated in the section allocated to the simulation.

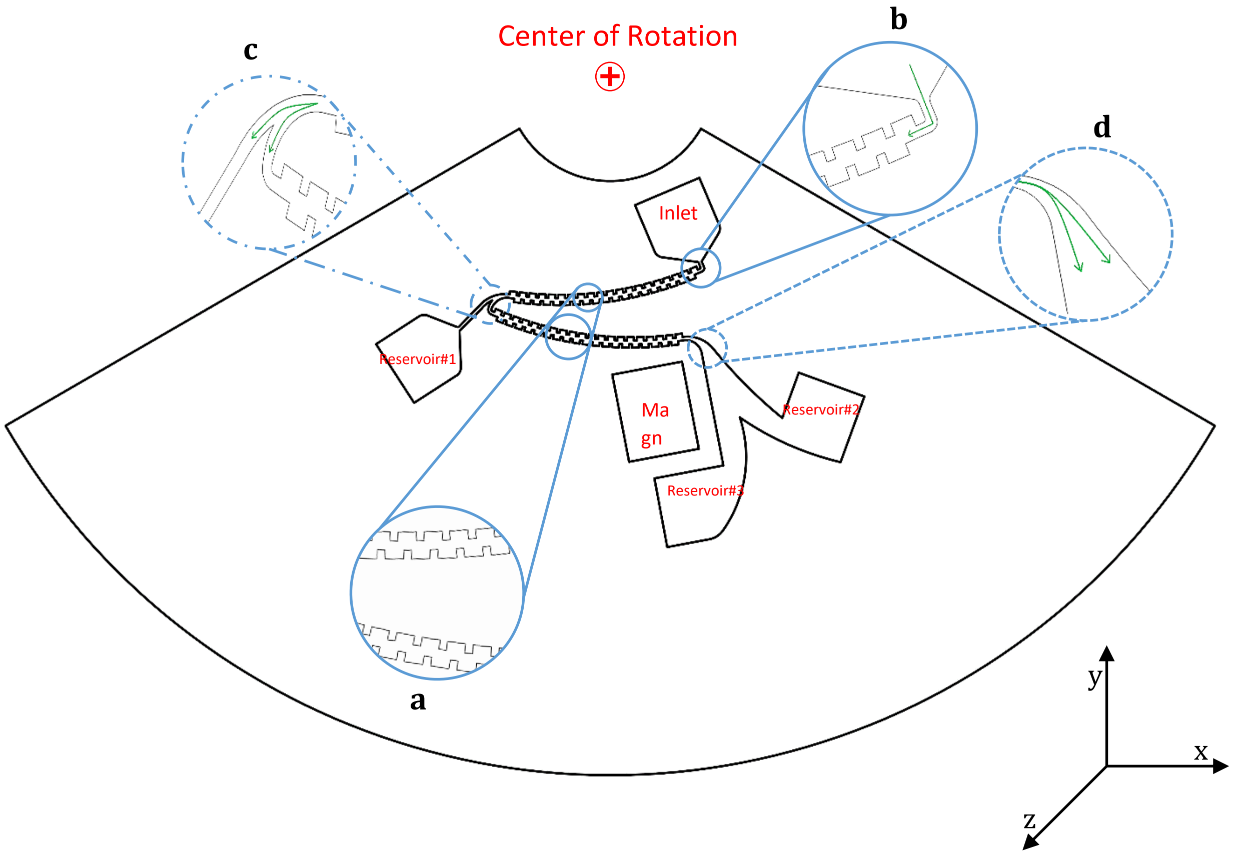

Figure S3.1: *The integration of all DPs on a piece of a compact disk (CD). Z-direction is the gravity direction.*

As for cell separation, cells can be sorted based on the size and density differences between the target CTCs and the other blood cells in the first section. However, they can be separated more by attaching modified Magnetic Nanoparticles (MNPs) to CTCs. In this case, magnetized CTCs are attracted by the permanent magnets and separate from the mainstream towards the target outlet.

The schematic of the final design is shown in Figure S3.2. Since all the components are placed by means of some screws and nuts, the proposed design is free of the challenge of the alignment of three layers in the micron accuracy. However, the multilayer structure of the device design could lead to leakages of the device. In other words, increasing the number of layers could lead to more leakage [49]. The leakage problem can be alleviated by reducing the number of layers, a typical issue with microfluidic systems [49, 50]. However, different mechanical and chemical solutions will be applied in this study and introduced in the subsequent sections to eliminate this problem eventually.

As can be seen from Figure S3.2, the current device was developed to have three layers. The lowest layer, called the substrate, is made of poly(methyl methacrylate) or PMMA. PMMA is also known as acrylic or acrylic glass. As the main body of the device, this layer consists of four same patterns to repeat the test four times with a proper weight balance. Such a measure can increase the accuracy of the results and assist in balance issues. The middle layer is the microfluidics tape for sealing the channels. Since the top and bottom layers are supposed to be stacked on top of each other, the design pattern, including the holes for screws and the place for magnets, will be carefully cut out by a knife plotter from this microfluidics tape. Then another device must be considered to apply uniform pressure to the tape, placed between layers. The highest layer, called the cap layer, is also made of PMMA.

As shown in Figure S3.2, all the components were intended to be arranged on the X-Y plane. Hence, the device was set out horizontally to reduce the possibility of leakage caused by the misalignment and no-bonding of layers. Moreover, considering six holes cut out from all three layers assisted in a better alignment. They were supported by screw/nut connections, which also helped seal the device and distributed a uniform pressure on the middle layer.

As shown in Figure S3.3, in agreement with the size of CTCs, the main channel was proposed to be 250 μm in width. The contraction-expansion chambers were designed in such a way as to maximize the effect of Dean drag force for lateral displacement while minimizing the maximum stress on cells, like the one of Ingber *et al.* [51]. Each contraction-expansion chamber, connected to the main channel, is 500 μm in length and 250 μm in width, while the depth of the device is 200 μm. The total height of the device (from the inlet to the target reservoir) in the x direction is 14.5 mm, which, in turn, optimizes the design in radial space and facilitates the insertion of the mixing device for cell lysis process or diagnosis purposes. The place of magnets is 6.35 mm in length and 5.08 mm in width.

It should be mentioned that the size of a standard compact disk measures 15 and 120 mm for inner and outer diameter, respectively, and it has a 1.2 mm thickness and weighs between 15-20 grams. However, since we will not add more functions to this research study, the outer diameter is considered 100 mm, which is still too much, so there will be vacant space.

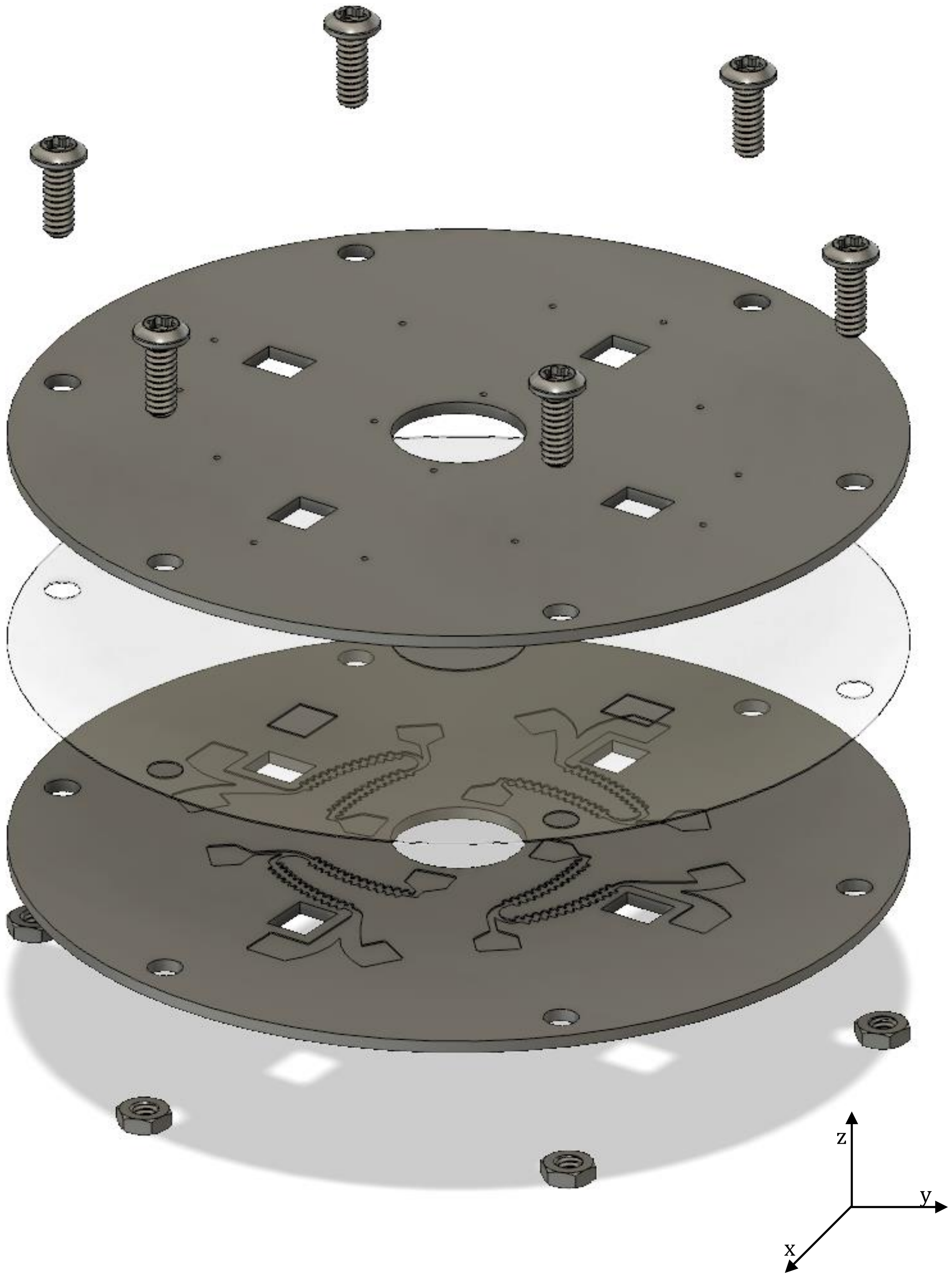
z
y
x

Figure S3.2: *The schematic of the device with two layers along with the middle microfluidic tape.*

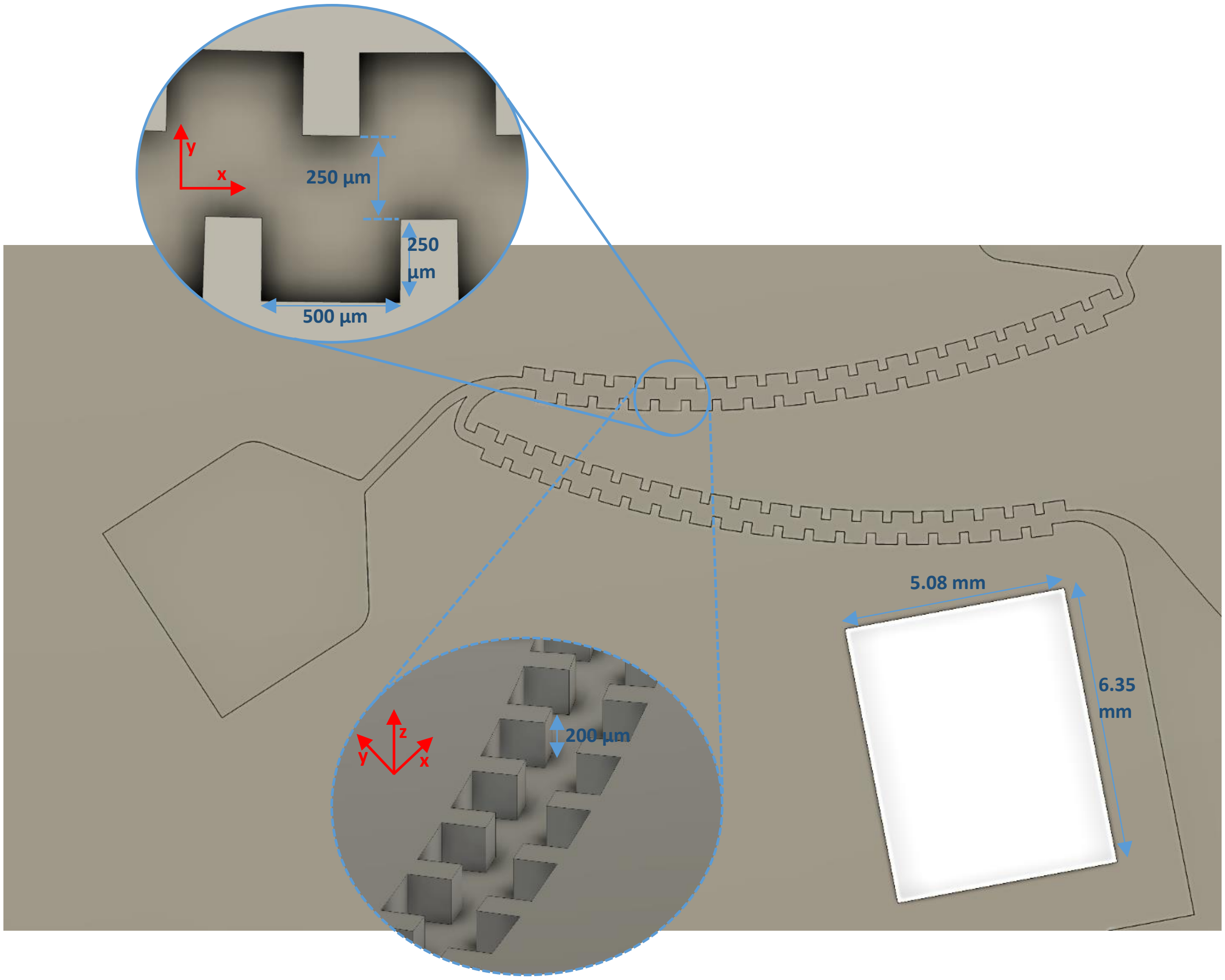

Figure S3.3: *The main microchannel of the device with stress reduction by inserting multiple contraction-expansion chambers.*

Not only the strengths and weaknesses of all microfluidic approaches should be comprehensively assessed, but also the manufacturing limits and accessible facilities should be considered before choosing a separation method. Among the passive separation methods, it is not possible to use the Pinched Flow Fractionation (PFF) and Deterministic Lateral Displacement (DLD) in this study [52]. One of the geometric features that play the most significant role in PFF separation is the very narrow width (less than 30 µm) of the channel in the throat area. Based on the available microfabrication techniques, it is not possible to create a channel with appropriate dimensions for this type of separation method. Conventionally, the lithography technique is the approach for creating these dimensions of the microchannels and the obstacles necessary in the DLD separation method. However, to the best of the author's knowledge, the fabrication of

microfluidic structures on a rotational platform with a 12cm-diameter disk using the lithography method encounters numerous limitations that have discouraged other researchers from pursuing this path. This method has been used less to create devices on the rotational platform.

In addition, creating the required obstacles in the DLD method is naturally challenging. Therefore, the passive separation methods used in this study follow bifurcation and inertial separation approaches. This section proposes creating secondary flows to sort cells on a streamline. The Dean drag force is proportional to the first power of the particle radius, while the inertial lift force is directly proportional to the fourth power of the particle radius [53]. Hence, particles with a larger radius (such as CTCs) will be more affected by the inertial force, while smaller particles (such as blood cells) will be more affected by Dean drag force. Since these two forces are applied in two opposite directions, the separation of cells with different sizes occurs in the first section. Therefore, a symmetric spiral microchannel structure with consecutive multi-orifice flow fractionations (MOFF) is used by considering contraction-expansion units on both sides of the main channel. Furthermore, the curvature of the main channel induces another centrifugal force, which can be integrated with other centrifugal forces generated by rotation to amplify the effective cell separation.

Among the active separation techniques, the acoustophoresis separation methods and the dielectrophoresis (DEP) phenomenon need a permanent electrical source to supply the necessary power to produce the acoustic waves or electric fields. This issue causes the producing actuators to be connected to the power source. Therefore, this connection makes it impossible to rotate the substrate on which the actuators are placed (the same substrate of the microfluidic system). In other words, employing these methods on the centrifugal substrate will not be possible. As such, the only active method that can be used in centrifugal microfluidic separators is magnetophoresis, which separates particles under magnetic fields generated by placing permanent magnets with defined strength in a specific location on the CD close to the bifurcation region.

Since the separation efficiency of passive techniques is lower than that of active strategies, this combination can increase the overall efficiency and cover the weaknesses of each method. Due to the channel geometry and flow profile in the first passive part of the current microfluidic separator, the target and non-target cells are placed on different streamlines. In the second active part, more deviation and greater distance can be made between these two cells using an external factor, which directs the cells to their destination reservoirs. Recent studies have shown that for particles whose diameter ratio to the microchannel hydraulic diameter is greater than 0.07, the forces originating from fluid inertia can be used for separation in a reasonable length of the microchannel under the inertial microfluidic separation method [54]. The remainder of this

section will explain the benefit from the inertial technique based on the required condition. In addition, the limitation related to the radial space occupied by the separator unit is another design factor that should be considered. Unlike the previous works, the current centrifugal microfluidic separation system will be designed in such a way as to use a combination of both active and passive approaches while occupying the least radial space and providing the highest separation efficiency.

In the first section of the device, where only passive methods have been used, separation is accomplished using the flow bifurcation method in the region where the particles decide whether to enter Reservoir #1 or the second section entry. Before reaching the bifurcation region, the more the target cells are placed in the further-away streamlines than the non-target cells, the better the sorting of the cells in the separation region and the higher efficiency of the initial separation will be. Therefore, before reaching this area, a number of sequential contraction-expansion arrays, known in the inertial techniques, have been considered on both sides of the main channel to improve the separation efficiency of the whole microfluidic device by achieving the desired sorting. Due to the constraint in the radial distance range for placing the isolation unit, the inertial section of the device has been extended as much as possible in an angular direction with a slight radial slope. The geometry of this section is illustrated in full detail in Figure S3.4. The minimum width of the channel in the current design is considered equal to 250 µm, which is 50 µm less than its uniform depth. This amount is chosen due to the approximate value of 150 µm, mentioned as the minimum width of such chambers in the relevant literature. Since the current device is supposed to be fabricated by two techniques (lithography method and computer numerical control (CNC) micromachines), and the thinnest available drill on the market has a diameter of 250 µm; thus, the smallest possible channel width will be equal to the purchased drill diameter. Comparing the results of two microfabrication methods gives researchers a better understanding of each method's capabilities.

Generally, the consecutive contraction-expansion chambers in the microchannel have been used to take advantage of the secondary flow effect on the separation phenomenon. In other words, the fluid flow is affected by the gradient of fluctuating and sudden lateral pressures while passing through this channel. Moreover, the suspended particles of different sizes in this flow experience different forces in the vertical direction. Abrupt and frequent changes in the channel cross-section change the flow direction and emerge the secondary flow. Therefore, these particles of varied size change their paths due to the effect of these lateral and vertical forces and are placed on different streamlines. Indeed, when differently sized cells are placed in a specific flow, they experience different levels of inertial force due to their different sizes. Consequently, under the effect of these different forces, particles of various sizes change their direction to be placed on different streamlines. Later, if these streamlines can be directed toward two or more different

outlets, the separation of differently sized particles is expected. The number of contraction-expansion chambers was determined by trial and error to maximize their effect on the fluid flow and separation efficiency.

In the second section of the system, the active separation method with the help of a magnetic field is combined with the passive techniques. In other words, the separation of cells can be observed using multiple contraction-expansion arrays and adding a magnetic field as an external factor in two forms of active and passive microfluidic systems. As shown in Figure S3.4, a relatively large region between the inertial and the bifurcation sections is considered to give the passing magnetized CTCs enough time and space to change their path under the magnetic field. The magnetized target cells are deflected under the strong magnetic field and pulled towards the desired reservoir, while the other non-target cells continue on their path. Adding magnetic properties to CTCs is done by attaching MNPs to the cells based on an exclusive antibody-antigen bonding. The main advantage of using a magnetic field is a significant increase in the system efficiency; however, the design occupies more radial space on the disk. As a result, the final device scheme has been done with the consideration of the location and design of other operational units, which are supposed to be installed in the following of this separator.

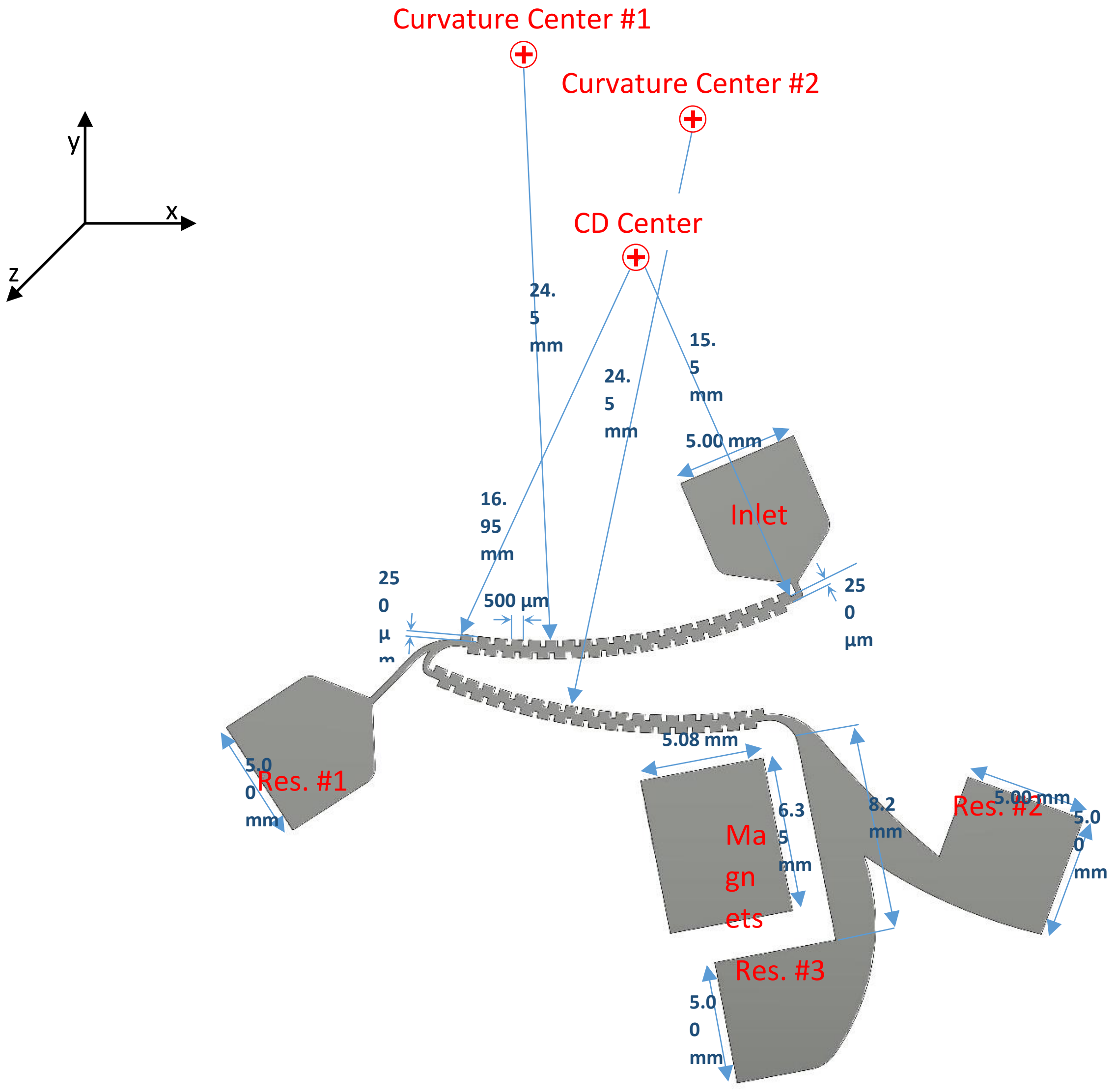

Figure S3.4: *An overview of the designed geometry for the hybrid centrifugal separation system.*

Supplementary Material S4: Fabrication (S4)

S4 Fabrication of the Microfluidic Separator

The techniques used for fabricating the devices presented in this study are indicated in this supplementary document.

S4.1 Lithography

Despite the advantages of soft lithography, it is an extremely challenging technique to fabricate the centrifugal microfluidic device, at least for five main reasons, which mainly originated from the Polydimethylsiloxane (PDMS). First, the attachment and alignment of the PDMS on the Si-wafer would be so challenging because it probably falls apart at high Revolutions Per Minute (RPM). The alignment of the PDMS on the Si-wafer with a simple pattern might not be a problem; however, the suitable handling for the alignment of complex patterns will be problematic according to the size of the features to be aligned. Second, PDMS is soft, so it is very likely that the channels will deform at high RPM as well (of course, some specific PDMS types can resist deformation at certain velocities). Third, PDMS/Silicon adhesions are fairly low, which makes it prone to delaminating when, e.g., fluidic connections are added. Fourth, the magnet will need to be integrated with the centrifugal microfluidic device; however, the procedure for this integration is still unclear. Fifth, the hyper-elasticity of PDMS is also one of the challenging issues in modeling PDMS buckling. In addition, soft lithography itself needs access to other microfabrication methods to create the starting master mold. These limitations have undoubtedly slowed down the industry's adoption process of soft lithography [55].

In this study, a novel photolithography technique was considered and completed in SyLMAND[1] to fabricate the centrifugal microfluidic device for the first time. There are several steps to achieve the final device through this fabrication approach: The photolithography process started with a wafer made of silicon material; however, the need for a photomask made us fabricate it beforehand. Therefore, the first step was preparing the photomask. For this purpose, the design with the desired dimensions modeled in Fusion 360® should be transferred to AutoCAD. Due to the fact that the UV light is supposed to pass through the mask and reach the photoresist layer, the parts of the design that are desirable for passing the light through should be transparent, while the undesirable portions should be opaque. To minimize stress due to material shrinkage, instead of preparing four devices in one continuous piece of 4” diameter SUEX[2] (DJ Microlaminates), each device was embedded in a rounded shape to minimize surface area and

[1] https://www.lightsource.ca/facilities/beamlines/cls/beamlines/sylmand.php#SpectralRange
[2] https://djmicrolaminates.com/suex/

prevent failure by shrinkage-induced delamination. Figure S4.1a shows a Cr-coated mask blank (Nanofilm 5” Cr mask blank–5X5X0.090 SL LRC 10M 1518 5K[1]) used in photomask preparation. This is a soda lime glass blank, 0.090” thick, coated with ~100 nm Cr and ~500 nm of AZ1518 positive photoresist.

The Cr-coated mask was then exposed to UV light in a laser machine (Heidelberg Instruments DWL-66+ laser writer with 355 nm laser, write mode IV–10 mm working distance) (Figure S4.1b) renowned for its accuracy and resolution. The accuracy of this machine is as high as 0.3 µm, which satisfies the need of this study. This step took almost 3 h. Later, the exposed photomask was developed in AZ400K developer for 1 min, rinsed with lots of millipore water, and dried by natural air. Then, the Cr was etched for 3 min in Transene Cr etchant, rinsed with lots of millipore water, Isopropyl alcohol (IPA), and millipore, and dried under an N_2 stream and natural air. The photoresist residue was then stripped with reactive ion etching (RIE) treatment (Plasmionique[2] FLRIE300-C, 30 sccm Ar + 20 sccm O2, 50 W, 5 min) (Figure S4.1c). The result of the photomask is shown in Figure S4.1d. The alignment markings (arrows at four corners of the device in Figure S4.1d) were added to align the mask to the wafer visually. The photomask could then be inspected by microscopes in terms of resolution and accuracy.

[1] https://www.nanofilm.com/
[2] https://www.plasmionique.com/home

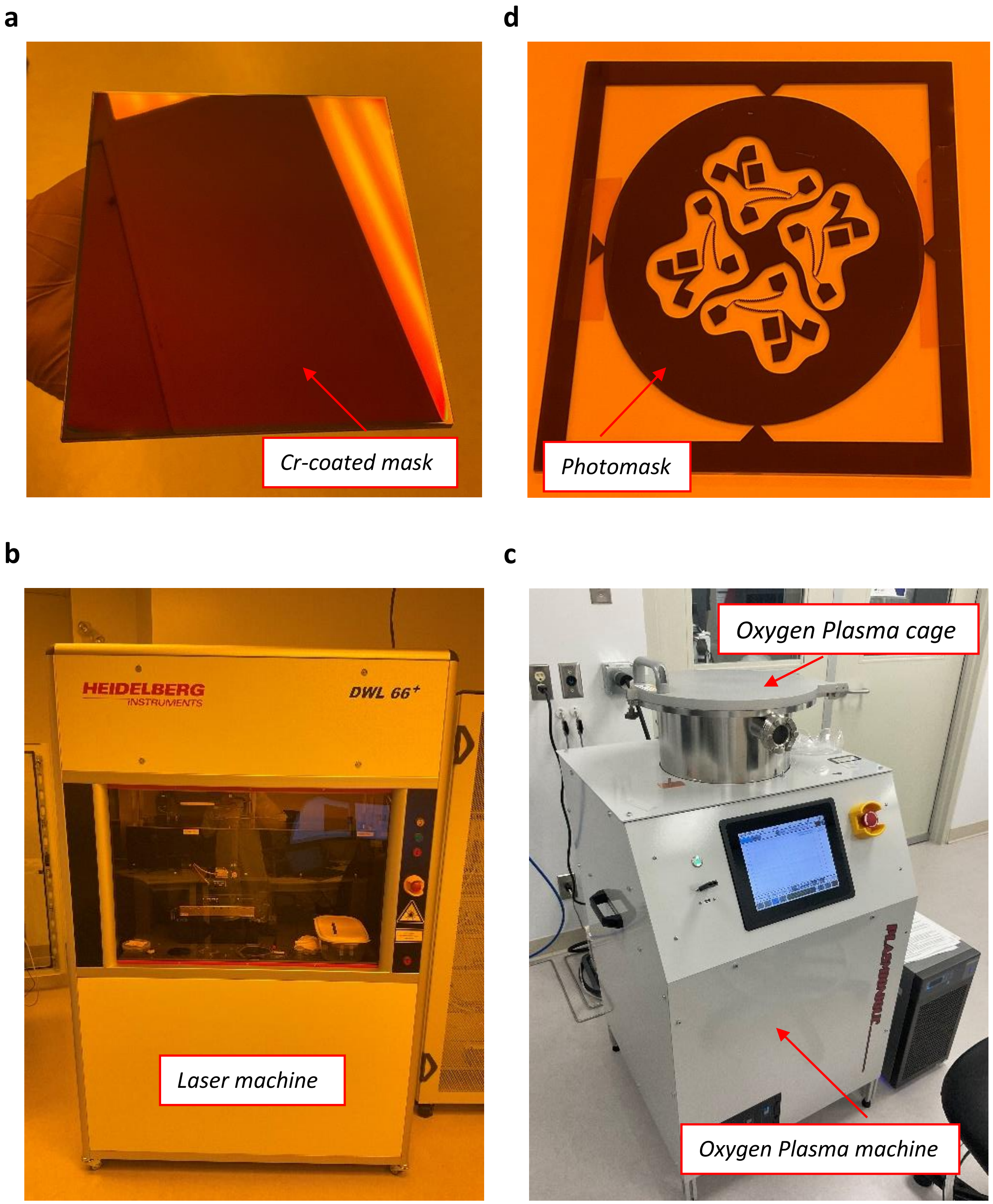

Figure S4.1: *The photolithography process in SyLMAND to fabricate the centrifugal microfluidic device;* ***a:*** *The pre-coated Cr-coated mask blank;* ***b:*** *The laser machine used for making the photomask;* ***c:*** *Reactive ion etching treatment in the oxygen plasma machine;* ***d:*** *The photomask created with the laser machine.*

In the second step, the top of the Si-wafer was coated with a negative photo resistance. The photoresist is usually spin-coated at a particular angular velocity to achieve a uniform thickness on the Si-wafer. However, in this study, the material SUEX was considered due to its unique properties, such as dry resistance, making it much more uniform than SU-8 (in terms of the surface), which, in turn, results in better and uniform channels and structures after UV exposure (Figure S4.2a). Also, the other advantage of SUEX is that it is rigid, but that too will have issues; for example, material shrinkage will need to be addressed, along with its effects on adhesion, which makes it a complicated problem in microfluidics. During this investigation, numerous attempts were made to resolve these issues and prevent airlock/bubble collection properly. To apply the SUEX to the wafer, the 4” Si wafer was first cleaned by N_2 stream and RIE treatment (Plasmionique RIE, 30 sccm Ar + 20 sccm O2, 50 W, 5 min). After that, a hot-roll lamination machine (Royal Sovereign Hot Roll Laminator Model-RSL 2702) was used with the experience-based chosen top and bottom roller temperatures of 70°C and 75°C, respectively, to laminate 200 µm thick SUEX (Figure S4.2b). The SUEX sheet plate was carefully placed in the middle of the Si-wafer and underneath the hot roller. The post-lamination bake would be required at 65°C for 10 min to remove bubbles in the film (Figure S4.2c). Then, the wafer temperature must be cooled to room temperature before moving to the subsequent deposition.

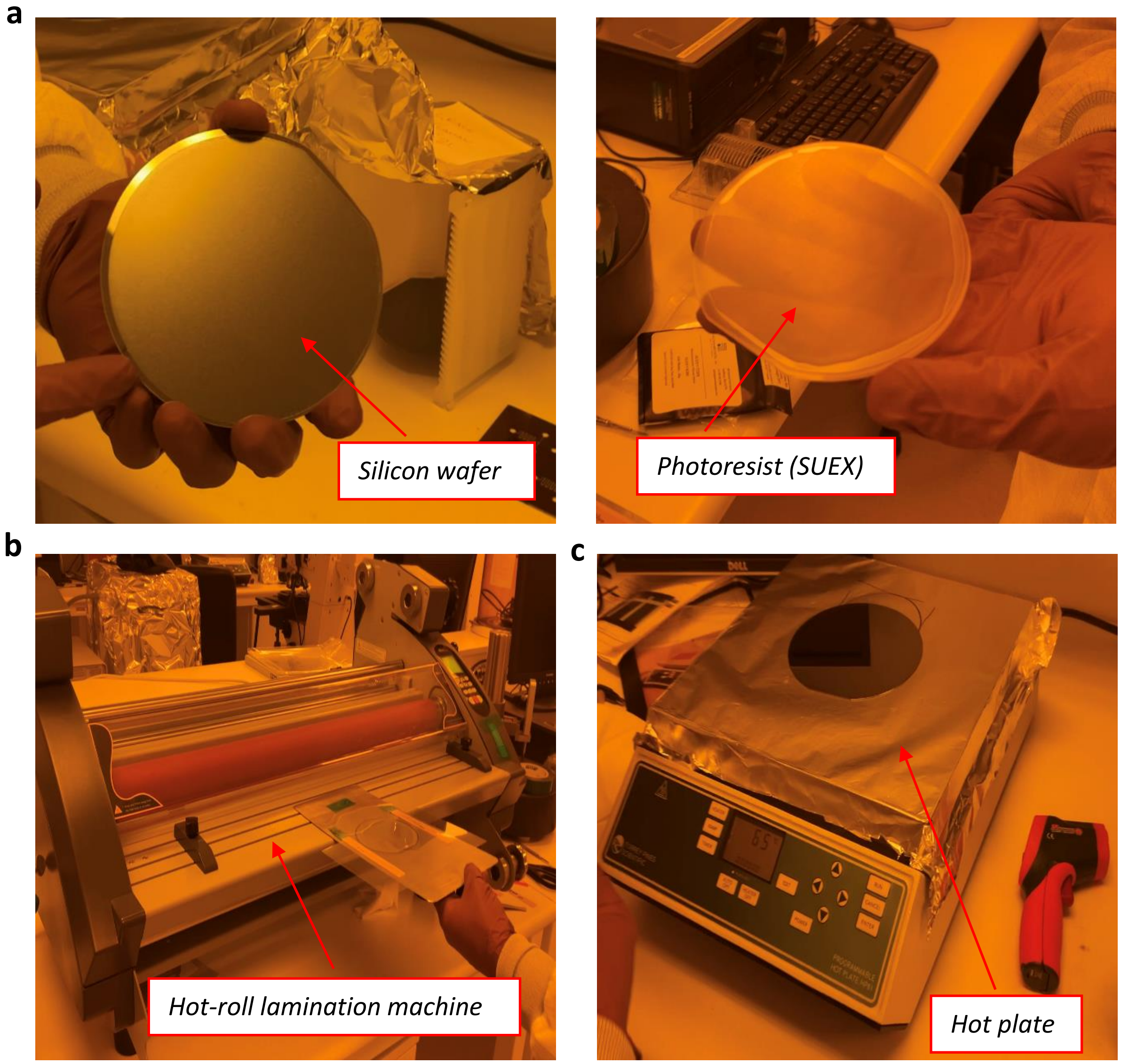

Figure S4.2: *The photolithography process in SyLMAND to fabricate the centrifugal microfluidic device;* ***a:*** *Silicon wafer (left side) and SUEX (right side);* ***b:*** *The hot-roll lamination machine;* ***c:*** *Post-lamination on hot plate (65°C) to heal bubbles.*

The next step is exposing the UV light to the photo-resistive material. The SUEX-coated Si-wafer and the photomask were aligned using alignment markings (Figure S4.3a) and placed inside the chamber of UV light exposure (UV light machine was UV light source Series30, Atand) (Figure S4.3b). The exposure dose, duration and power were 1350 mJ/cm^2 with low pass filter to improve sidewall quality, ~300 s, and 4.4 mW/cm^2, respectively. After that, the whole system was baked in an oven at a temperature of 85°C for 35 min (Figure S4.3c). Finally, it was allowed to cool down to room temperature (25°C) for 8 h to minimize stress.

After exposure, the Si-wafer is ready for development. At this stage, the UV-exposed photo-resistive system was face-down submerged for 13 min with no agitation in SU-8 developer (propylene glycol methyl ether acetate, PGMEA) (Figure S4.3d). Finally, it was rinsed with lots of fresh developers, followed by lots of IPA solution, then placed face-down in an IPA bath for 5 min with no stirring (Figure S4.3e). The post-development bake was carried out by vertically drying in the oven at 100°C for 5 min to remove IPA and solvent residue (Figure S4.3f). The hard-bake step was also advised to strengthen resistance for future applications. The whole device was hard-baked at 150°C for 30 min and allowed to cool slowly overnight to room temperature to minimize stress (Figure S4.3g). At this point, the fabricated device (Figure S4.3h) could be inspected by microscope for delamination and residual resist and would be used for the subsequent sealing procedure. As can be seen, the four devices were separated into discrete shapes by drawing a curving boundary around them, which was done to minimize stress in the SUEX film and avoid shrinking during the process. Indeed, decreasing the surface area of each device helped prevent delaminating the areas covered in SUEX due to shrinkage.

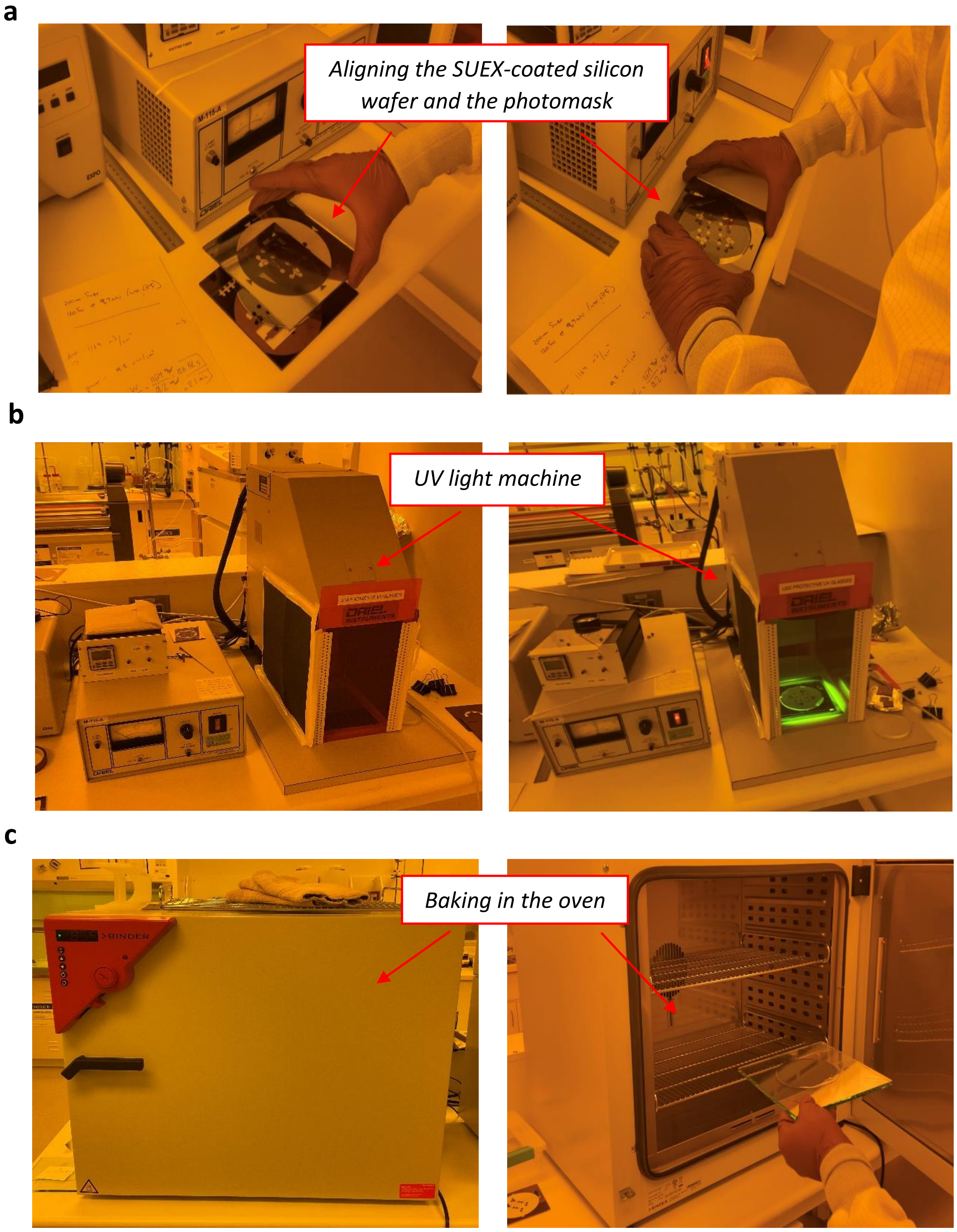
a
Aligning the SUEX-coated silicon wafer and the photomask
b
UV light machine
c
Baking in the oven

d **e**

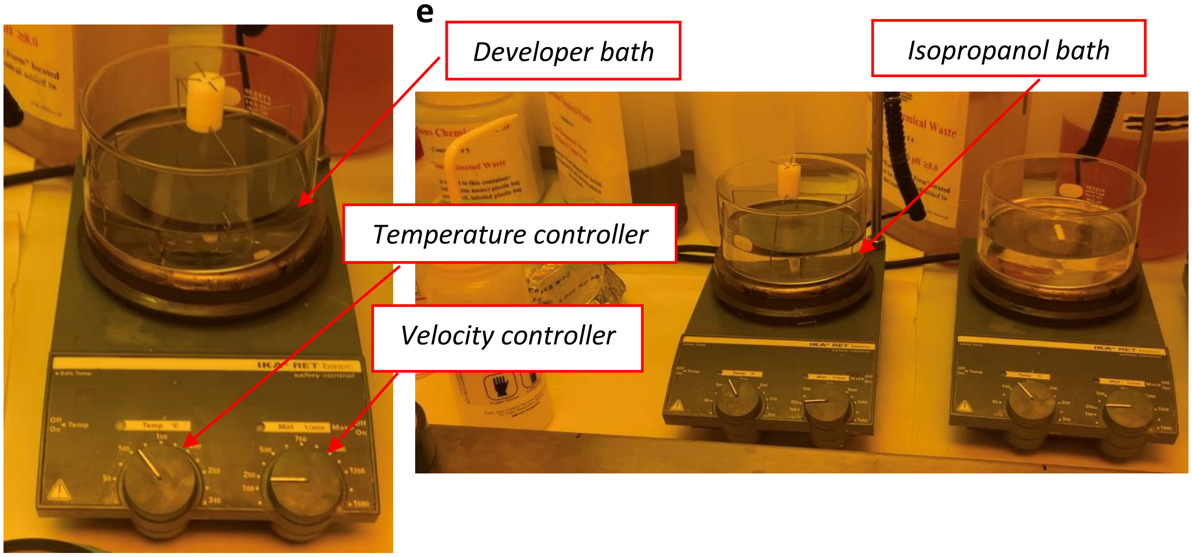
Developer bath
Isopropanol bath
Temperature controller
Velocity controller

f

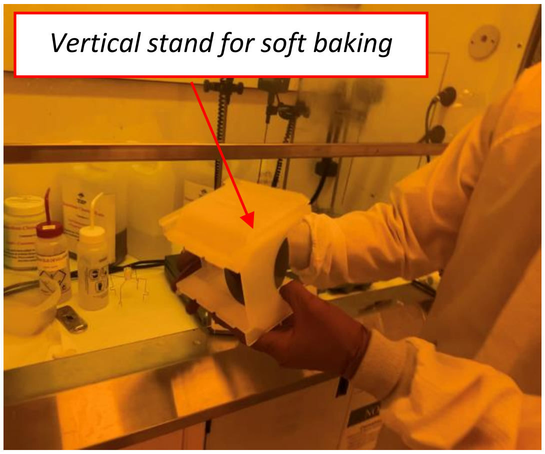
Vertical stand for soft baking

g

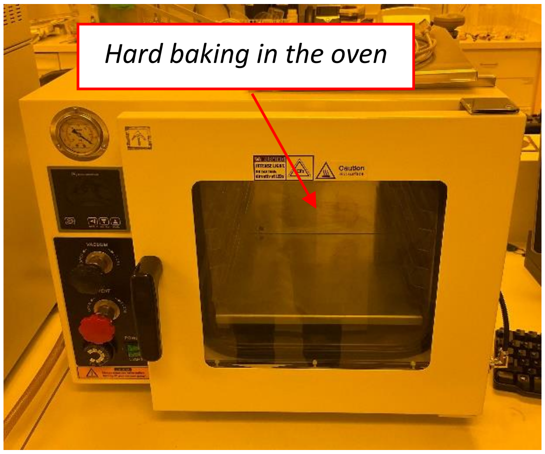
Hard baking in the oven

h

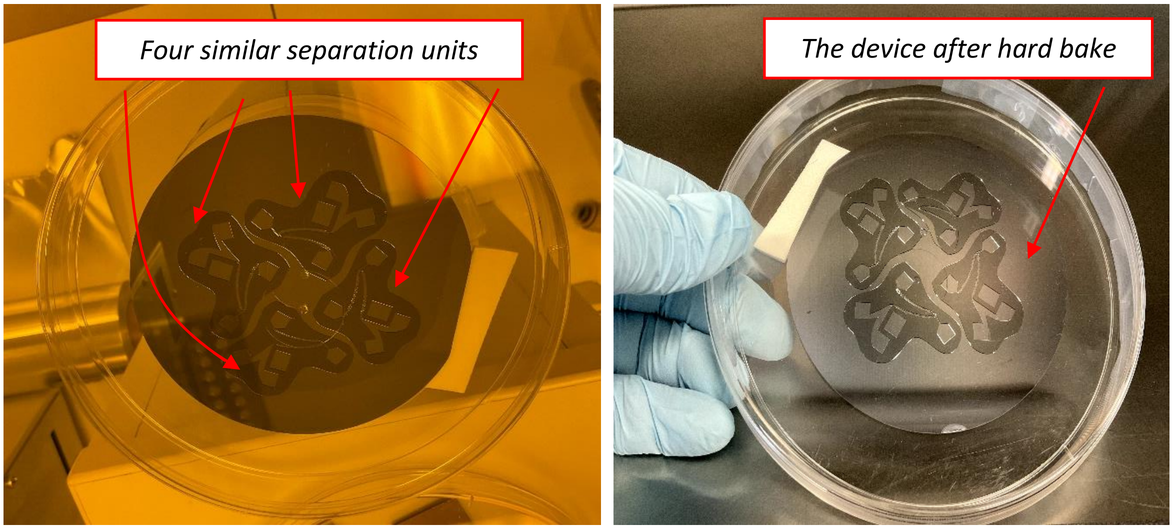
Four similar separation units
The device after hard bake

Figure S4.3: *The photolithography process in SyLMAND to fabricate the centrifugal microfluidic device;* ***a:*** *Trying to align the SUEX-coated silicon wafer and the photomask;* ***b:*** *UV light source;* ***c:*** *Post-exposure baking (30 mins at 85°C) in an oven;* ***d:*** *The development of the silicon wafer;* ***e:*** *Immersing the substrate in an Isopropanol bath;* ***f:*** *Post-development baking by vertically drying in an oven (5 mins at 100°C);* ***g:*** *Hard baking (30 mins at 150°C) in an oven;* ***h:*** *The completed device hosts for four similar geometries on the silicon wafer after hard bake and ready for the bonding to the microfluidic tape.*

Now, the appeared channels on the Si-wafer need to be covered, and this can be achieved by using a state-of-art hydrophilic tape (ARflow 93049[1]) designed for sealing biomedical devices and compatible with SUEX (Figure S4.4a). Fabrication considerations, tape thickness, and cost/availability were the main remarks taken into account when choosing this type of tape. For example, some tapes are generally better suited for thin applications (<50 µm), including cellular and biochemical assays in which reagents are costly, whilst others are more suitable for thick applications (50–200 µm), including cell culture in which media must be slowly perfused over cells with minimal shear [56]. The shape of the geometries and the openings for the magnets were traced out and cut from the microfluidic tape with a combination of a scalpel and scissors. Then, the sacrificial layer (bottom sheet) of the tape was removed and carefully fixed to each device by lining it up by eye (Figure S4.4b). A straight edge (a razor blade in this case) was used to burnish the tape by carefully pressing the razor blade along it to smooth it down and improve adhesion (Figure S4.4c). In case of replacement need, the damaged/polluted tape could be replaced by a new one by easily peeling away a section of tape on the sealed device. Figure S4.4d shows the result of four devices after bonding.

[1] https://www.adhesivesresearch.com/healthcare/microfluidics/

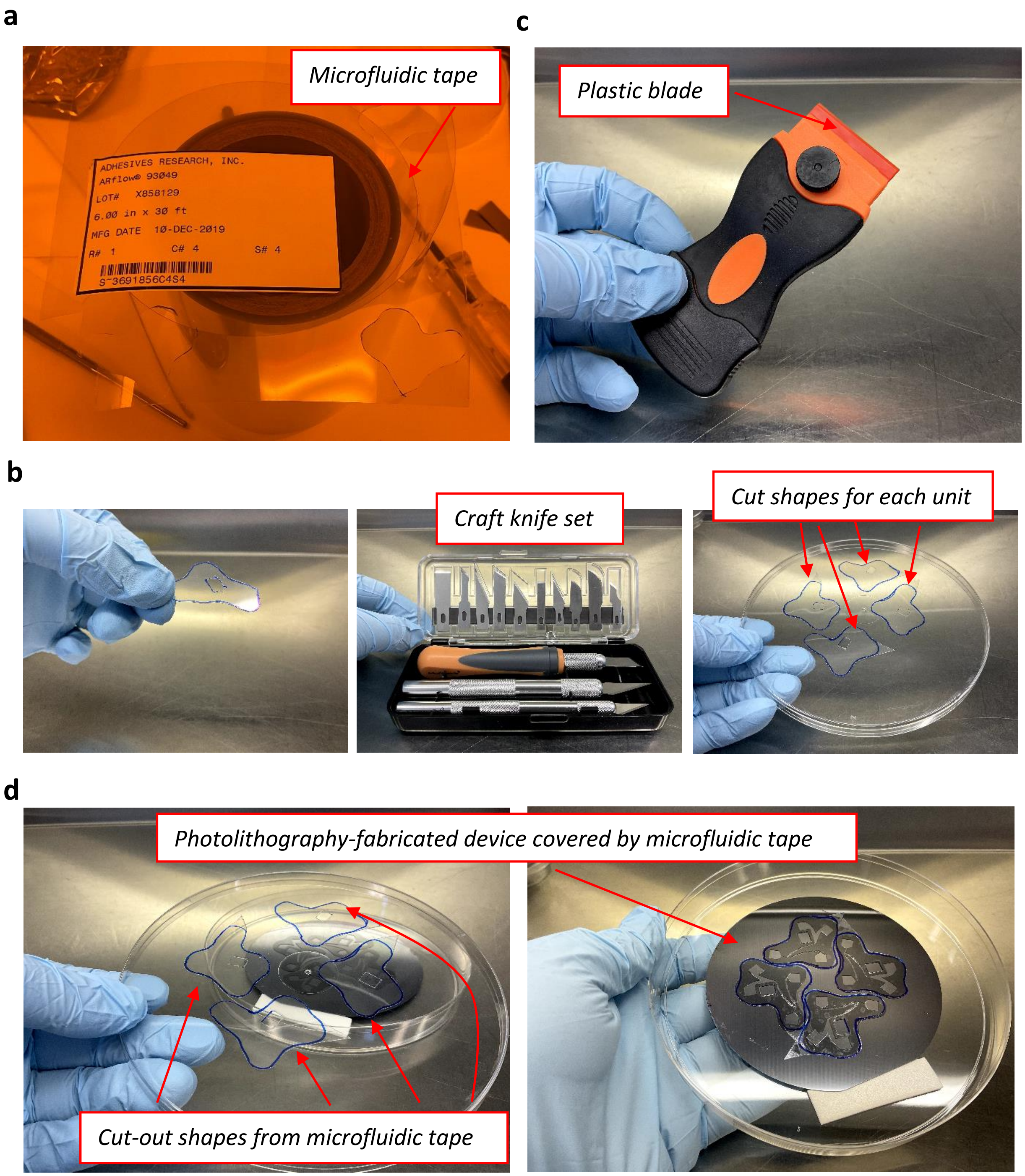

Figure S4.4: *The tools used in fabricating the centrifugal microfluidic device;* ***a:*** *Microfluidic tape;* ***b:*** *The cut shape of geometries and the precision craft knife set for modeling;* ***c:*** *Plastic blade used to smooth the tape down and improve adhesion;* ***d:*** *Centrifugal microfluidic silicon wafer with four separation devices.*

Although the most common concern in conventional photolithography and soft lithography is the bonding process and alignment to make a microchannel which must be completed within a couple of seconds, it was not even an issue in the current work because the channel could be sealed and closed by the specific type of microfluidic tape. This tape also resolves the leakage problem in the multilayer-assembly fabrication as another common challenge. Furthermore, since the depth of the current channel was considered 0.2 mm, the design had no thin-narrow features to trap bubbles or cause problems.

S4.2 Micromachining

In this study, the method of micromachining with Computer Numerical Control (CNC) machines is also chosen to construct designed microchannels and the centrifugal microfluidic separation device and make a comparison by the photolithography-fabricated device. Two fabricated devices by photolithography and micromachining approaches are supposed to be compared and evaluated in the next section in terms of cell separation efficiency and fabrication challenges. According to the simulation and modelling section, the prepared cutting tools are flat-tip end mills with a cutting diameter of 250 µm. Therefore, the design of the microchannel width, contraction-expansion chambers, and simulations were also done based on the same diameter.

In the machining technique, the raw material used for this separation system is plexiglass (PMMA) disks with a diameter of 100 mm and a thickness of 1.2 mm, which are subsequently joined together to make the final microfluidic device. As a simple and useful plastic, PMMA takes advantage of low cost, high optical clarity, availability, and compatibility with numerous manufacturing instruments [56]. Numerous advancements in the microfluidics field, from fabrication, and functions, to operations, have caused a potentiality to facilitate the development of PMMA microfluidic applications [57, 58]. Furthermore, although the two materials, PMMA and Polycarbonate (PC), were among the initial options for the substrate of the device, PMMA was finally picked due to the possibility of better chipping compared to PC and its other superior properties.

As mentioned before, the diameter of the current device is smaller than that of actual commercial disks (120 mm), as the current study is only intended to focus on the separation unit. However, the diameter of future devices comprising other functional units (such as lysis and mixing units) can be increased. Another reason for choosing this diameter originates from our purpose to reduce the material and cost of fabricating the holders and disks. The other reason is that the Si-wafer, which is used in the photolithography techniques, is only available at 100 mm; as a result, we concluded to reduce the diameter size of the plexiglass disk to compare these two techniques and corresponding devices fairly.

CNC machines can be integrated with microscopic monitoring to fabricate microchips with a high precision factor on machined structures of about 2-10 µm. The depth of the channel in the z-axis direction should be pre-calibrated for structures with an accurate aspect ratio. According to the dimension and shape of the microchannels, the desired size is obtained by cutting tools with different drill bits. The possibility of breaking the drill bits with a very small diameter is very high during the turning process; thus, choosing the proper size of the drill and the angular velocity for the CNC machine is the most crucial factor in avoiding breaking the drill head. Indeed, the breakage probability during the lathing process at a low angular speed of the machine is increased due to the drill head imbalance. However, this does not mean setting a higher speed for the CNC machine is always better. In fact, an optimal RPM range should be determined according to each drill diameter. In this study, the microfluidic device was made by turning with a Yama Seiki BM-1020 CNC machine (Figure S4.5).

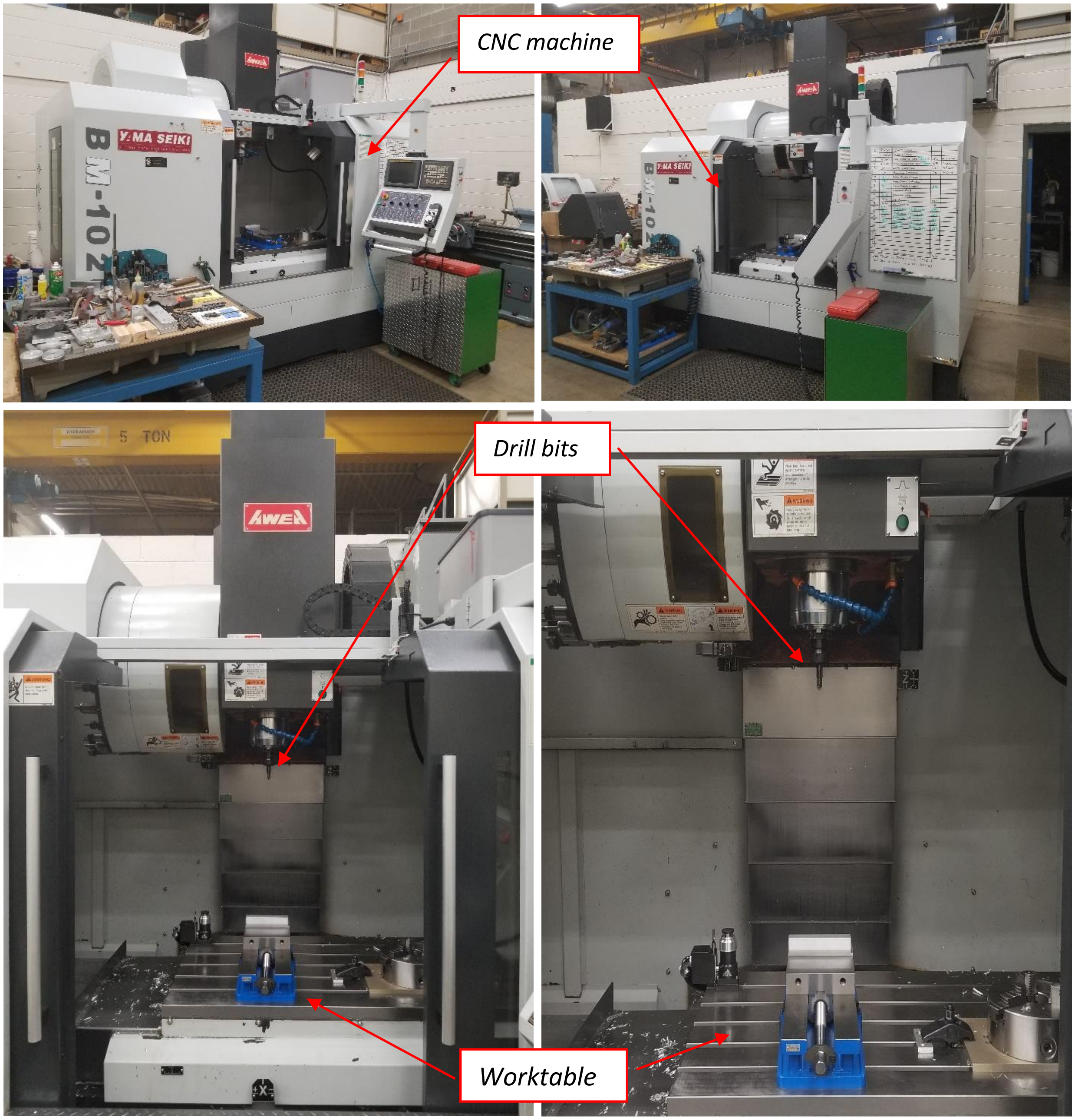

Figure S4.5: *A view of the CNC machine for creating the centrifugal microfluidic device.*

Figure S4.6 shows the lathed PMMA disks with the considered holes for inserting the permanent magnets.

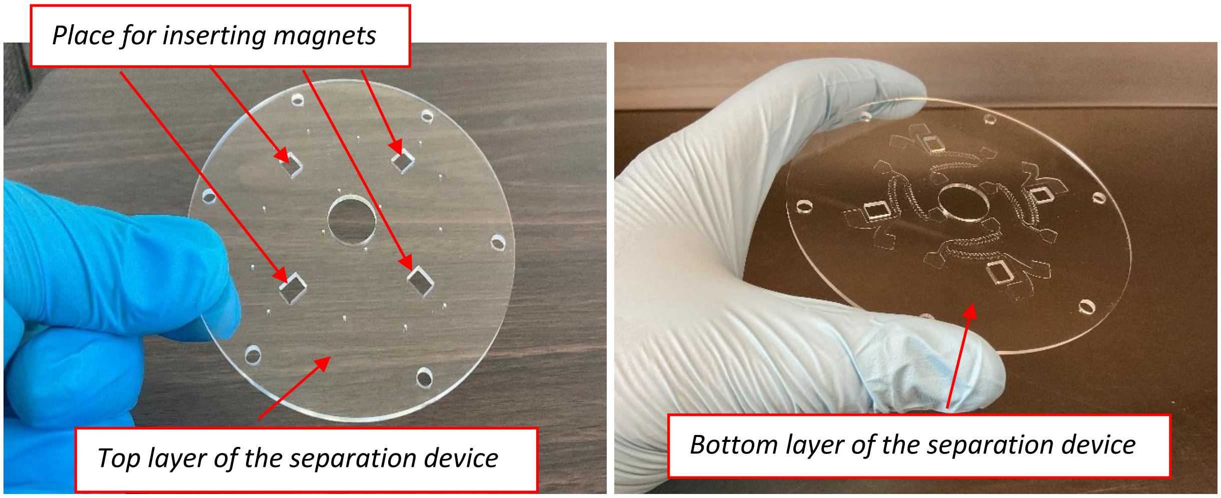

Figure S4.6: *The two top and bottom layers of the microfluidic separation device created by CNC micromachining on PMMA sheets.*

S4.2.1 Surface finishing

The fabrication of the microfluidic system using the CNC machining method creates surface roughness on the channel surfaces due to the drill movement, negatively affecting the fluid flow and cells with a size of less than 20 µm. These negative effects have been mostly neglected by researchers [59-61]. The tool marks left on PMMA substrates could also be increased in case of using smaller drill bits. The surface smoothness of the CNC-micromachined substrate depends on numerous parameters, from the spindle speed, the feed rate, and the cut of depth to the operating environment. In recent years, some efforts have been made to eliminate this surface roughness by using the vapor of PMMA solvents, such as acetone, ethanol, and chloroform [62]. In this study, all three solvents were employed and investigated for this application, finally leading us to use acetone. Indeed, during this examination, it was noticed that a better surface quality was achieved using chloroform and ethanol than acetone due to their high solubility, which, in turn, increased the sensitivity of this work because they could cause the channels to undergo corrosion and weaken/destroy the channel depth. In the end, it was found that the acetone steam could successfully polish the surface with acceptable quality and less damage to the microchannels. Figure S4.7 shows an image of the steam spray system, which was designed and implemented at the chemistry department of the University of Saskatchewan.

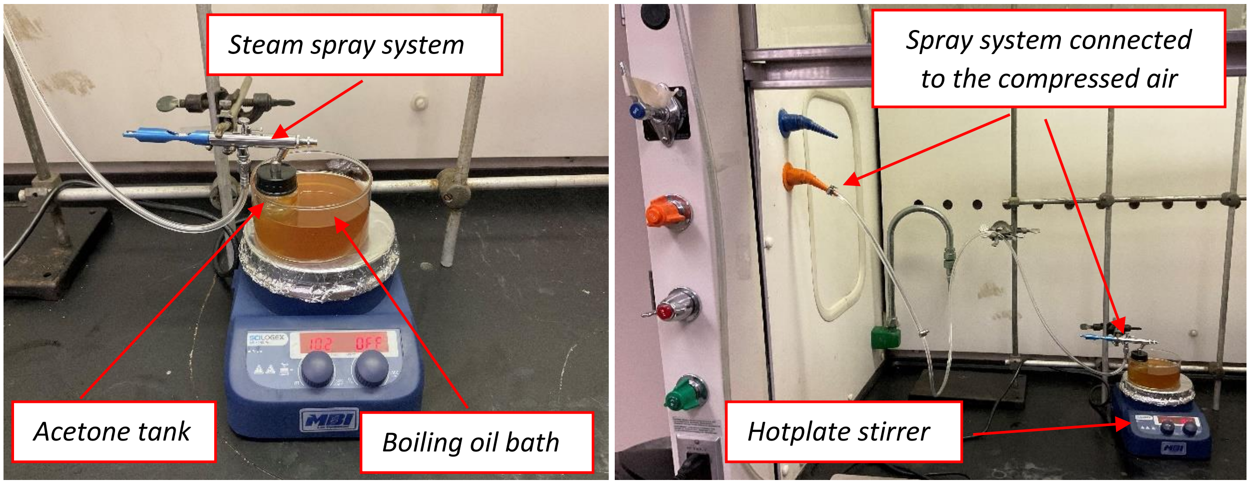

Figure S4.7: *The vaporized acetone spraying system.*

After filling the spray tank with acetone and putting it into the boiling mineral oil dish, the acetone turns into a steam phase and is sprayed out from the spray nozzle (the boiling point of acetone is 56°C [63]). According to the valuableness of the final centrifugal microfluidic device and its damage possibility at this stage, another microfluidic device with the same channel width and depth but with more channels was designed and used to check and test this issue (Figure S4.8a). The purpose of using this second device is to ensure the correct selection of solvent, distance and duration of spraying, number of surface finishing steps and other relevant parameters. Microscopic images of the microchannel surface before and after surface finishing are shown in Figure S4.8b and Figure S4.8c. After this step, similar surface finishing experiments will be repeated on the primary centrifugal microfluidic device.

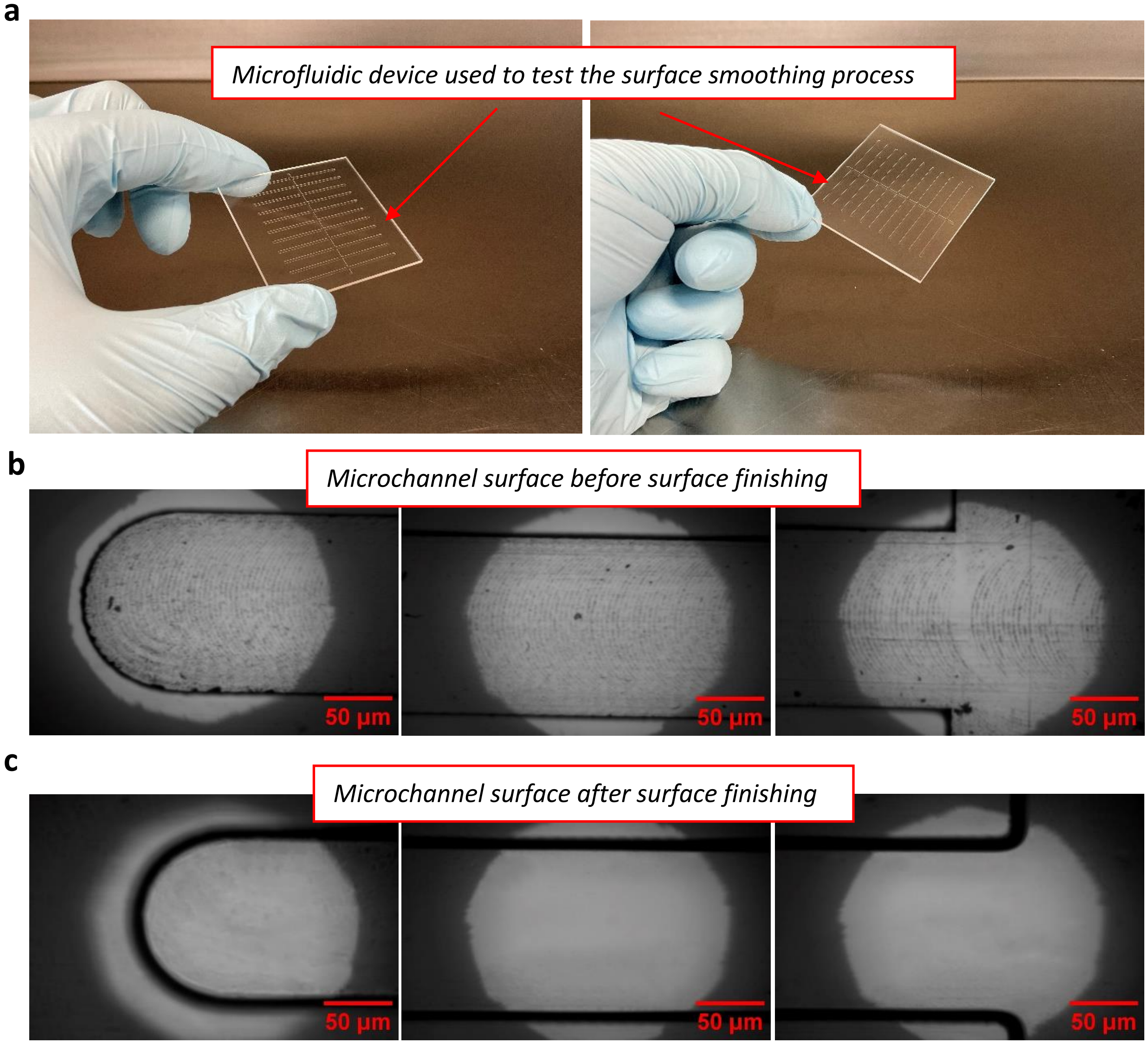

Figure S4.8: *Images of the microchannel surface made by the machining technique, **a:** A designed microfluidic device with several channels to show the result of the vaporized acetone spraying system; **b:** Before surface finishing; **c:** After the last step of surface finishing (Photos were taken by inVia™ InSpect confocal Raman microscope[1]).*

S4.2.2 Layer bonding and sealing

After building the system through photolithography and machining approaches and polishing the channel surfaces with vaporized acetone spraying, attaching another layer to make up the final separation device would be the most important stages of fabrication. This stage is crucial in

[1] https://www.renishaw.com/en/invia-inspect-confocal-raman-microscope--45378

employing microfabrication techniques in broader industries and applications by saving associated process time and costs. Layer bonding, leakage, and alignment are some challenges that must be mindful of. Unlike the previously manufactured centrifugal microfluidic devices [59-61, 64-67], the current proposed centrifugal separator is within the safe margin in terms of leakage. Such a certainty originates from designing and fabricating the device with fewer layers and using a tried-and-true approach for layer bonding after testing several ways. Thermal bonding and adhesion with the help of UV light and microwave are conventional techniques to connect PMMA to PMMA, requiring special and expensive equipment [68, 69]. The usage of thermal bonding methods can lead to the deformation of the microchannel, known as one of its inevitable consequences [68, 70]. In this study, some applicable methods were evaluated and tested. A brief introduction of each method is mentioned below.

Binding with UV Activated Glue:

An important point in using these glues is that the adhesive layer poured between the surfaces should have a thickness of about 10 µm to make a sufficient and acceptable attachment. If the thickness of the glue is less than that, the connection will not be formed well. While if its thickness is more than the mentioned value, a little flowing glue will remain between the two surfaces even after UV light exposure. Despite the advantages of this method, it was not a suitable option for attaching two machined layers because the microchannel was blocked with glue during the bonding test. One of the other reasons for abandoning this method goes back to the lack of suitable facilities. As a matter of fact, due to the available equipment, there was insufficient precision in making a uniform and optimal distribution of glue on the surfaces to reach the appropriate thickness.

Binding with chloroform:

The plexiglass material used to create the centrifugal microfluidic system (under the machining fabrication method) is dissolved in chloroform. If the second layer is immediately placed on top of the first layer when the first surface contacts the chloroform, the two layers are unified with suitable sealing while performing the surface dissolution process. The main limitation of using this method is channel corrosion caused by PMMA dissolution upon contact with chloroform. However, the results show that if the gluing process is quickly done, while the internal space of the microchannel is drained of chloroform immediately after the operation, the layers are well attached and sealed without damaging the microchannel. It is important to note that there are only about one to two seconds for layer alignments after pouring chloroform on the plexiglass surface. The main destructive factor affecting the microchannel in this study was the inability to empty the system from chloroform in time. Accordingly, it was the main reason this chemical substance was not used to attach the machined layers of the centrifugal microfluidic system.

Binding with isopropyl alcohol:

Like chloroform, isopropyl alcohol can also dissolve PMMA; however, the difference between these two solvents is that alcohol is much weaker than chloroform. In addition, the rate of PMMA dissolution in isopropyl alcohol is a function of temperature. This issue served as a springboard for the researchers to employ this alcohol to create a microfluidic system with a high layer bonding strength and suitable optical clarity [68, 71, 72]. This method starts with thoroughly washing the bonding surfaces and removing possible contamination. They are then dipped in a 70% solution of isopropyl alcohol and placed under the pressure of two paper clamps for 15 min in an oven set at a temperature of 68°C to reach the desired layer bonding. Under such a procedure, due to the lower thickness and larger bonding surface area of the current microfluidic device, the idea of using paper clamps did not lead to favorable results because the pressure on the contact surface could not be uniformly distributed. Thus, this method cannot be a suitable choice for our ongoing research.

Binding with pressure-sensitive microfluidic adhesive tape:

One of the state-of-the-art methods of bonding the PMMA layers of microfluidic systems is to use pressure-sensitive tape. This method works by placing a layer of adhesive tape between the two surfaces that make up the microfluidic system. Then, the whole stacked layers will be pressed under a mechanical pressure application and remain under pressure for about 12 h. Under this mechanical pressure, the adhesive tape properties are activated, and the layers bond well to each other. Meanwhile, the possible air bubbles trapped between the layers also have enough time to escape.

The most up-to-date available versions of these tapes are one- and double-sided adhesive tapes specially designed for bonding microfluidic architecture to substrates or together [56]. The advantages of these adhesive tapes are that their one- and double-sided types can be used to close the microchannel top surface of the photolithography-made centrifugal microfluidic device and bond two CNC machined layers to each other, respectively. In a one-sided tape scenario used for a photolithography-made microfluidic device, due to the fragility of the silicon substrate, a spatula (or a razor scraper with a plastic safety blade or carbon steel blade, see Figure S4.4) can be used to exert a low controlled pressure, stick the tape layer on the surface, and remove the glue residue (instead of applying the pressure by a mechanical device). The noteworthy point is that before placing the adhesive tape between the layers of the microfluidic system, it is necessary to cut the channel designs out from the adhesive layer (either by a cutter plotter (Figure S4.9a) or by a razor (Figure S4.4)) before placing it on the silicon wafer. Figure S4.9a and Figure S4.9b show the knife plotter (a Silhouette CAMEO® 4 Cutting Machine[1]) and the cut-out

[1] https://silhouettecanada.ca/pages/cameo-4

patterns, respectively. To the best of the author's knowledge, the main reason for not widely using this tape for connecting microfluidic surfaces in the past was due to the lack of a cost-efficient cutter-plotter. Due to the nature and characteristics of the microfluidic tapes, the fashion-old cutter plotter machines were incapable of cutting channel patterns with complex geometries and narrow widths (below 200 µm). Hence, in the past, these adhesives were mainly used for simple microfluidic designs (e.g., a reservoir and a simple channel), providing a satisfactory connection between the surfaces (in terms of cost and quality). In recent years, in addition to reducing the costs of building cutter-plotter machines, their resolution and accuracy have significantly improved with the advancement of technology.

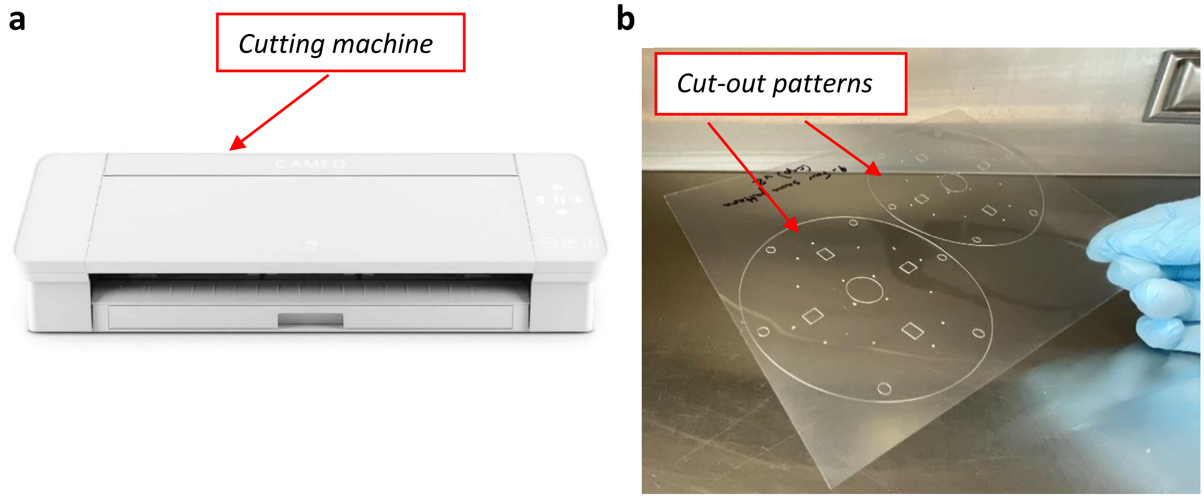

***Figure S4.9:** Cutter plotter used to cut out the geometry shape from a microfluidic tape; **a:** The cutting machine used in SyLMAND; **b:** The cut-out patterns from the microfluidic tape.*

Furthermore, a novel device (Figure S4.10) was designed and developed to apply a uniform distribution of mechanical pressure on the surfaces that are going to be bonded (whether using pressure-sensitive double-sided adhesive or even solvent or heat-assisted solvent methods). This set consists of two compressed Gray Polyvinyl Chloride (PVC) plastic components with guiding pins to help with the adjustment and alignment of the layers. Indeed, in addition to applying uniform pressure, aligning the two disks so that the designed elements fully face in front of each other is essential. Therefore, five holes considered for installing the screws in the center and corners of this device, along with the pins installed on the surface beneath the layers, will assist in aligning the layers of the system, distributing uniform pressure on the surfaces, and fixing their positions on the press surface.

It should be noted that although the bonding and sealing of the layers are done using this tape/device, the pressure exerted on the layers must be controlled; otherwise, there is a possibility of breakage at different points of the disk. Therefore, after designing the proposed

press device in Fusion 360®, the force analysis module in COMSOL® was used to examine the uniformity of the desired pressure on the microfluidic disks. In this software, the stress distribution on the disks was simulated and computed for the maximum feasible applied force (i.e., 1000 N). In other words, the analysis was conducted under a constant force of 1000 N, applied to the disk as a distributed load, after contacting the surfaces of the press device with the upper and lower surfaces of the microfluidic disk. Moreover, density, Young's modulus and Poisson's ratio for PMMA were taken as 1.19 g/cm^3, 3 GPa and 0.4, respectively. As seen by the contour plot of von Mises stress in Figure S4.11, the stress on the disk has been distributed uniformly between 0.26 MPa and 0.35 MPa. As a result, it can be concluded that the requisite uniform pressure can be applied by using this press machine.

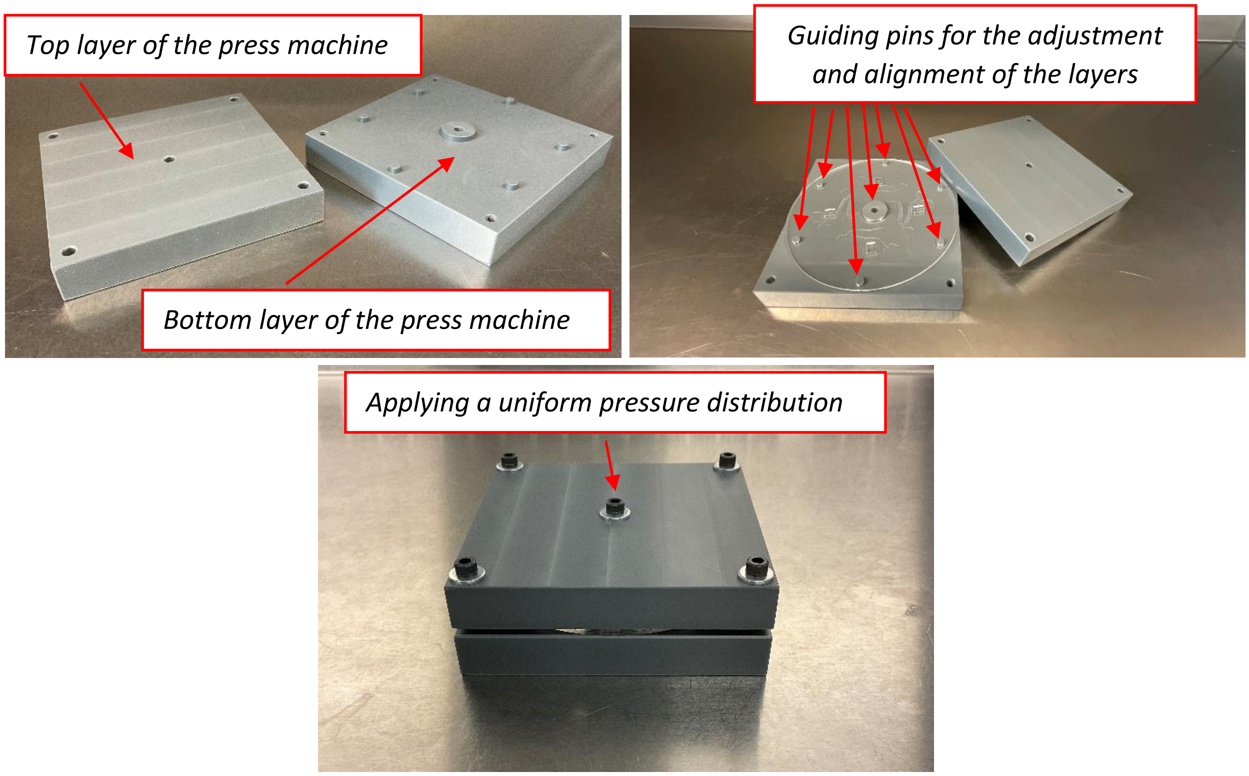

Figure S4.10: *The compressed Gray PVC components apply a uniform distribution of pressure on the surfaces.*

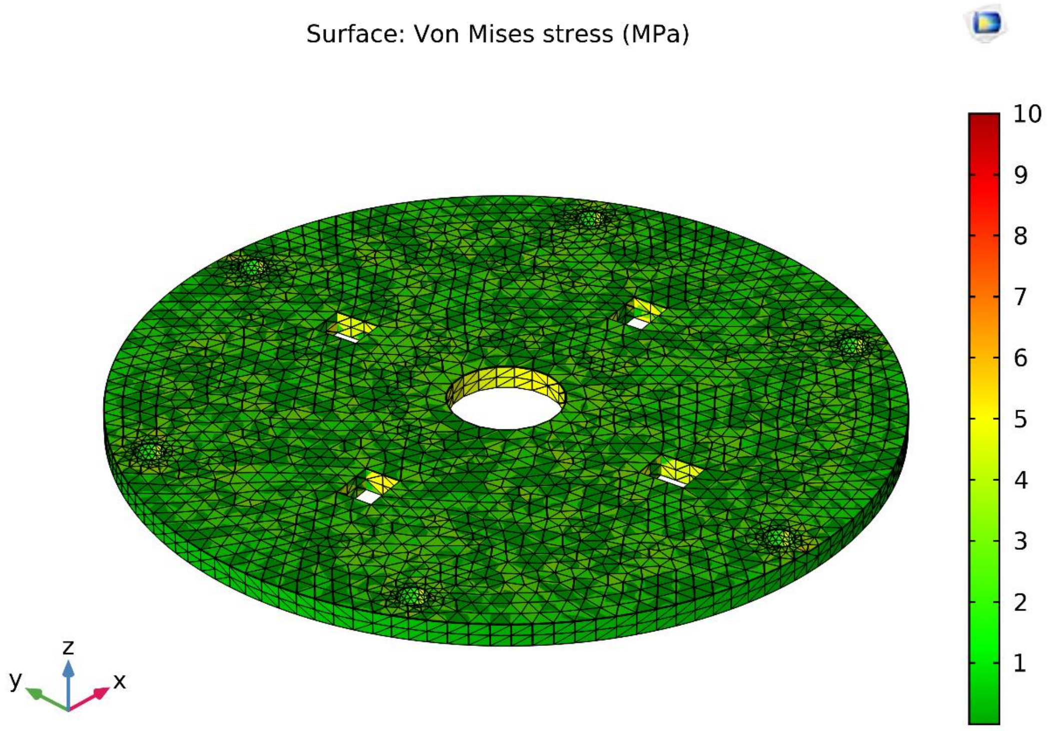

Figure S4.11: *The von Mises stress contour on the microfluidic disk by applying 1000 N.*

Supplementary Material S5: Experiment/Test (S5)

S5 The Cell Culture

Breast cancer cells (MCF-7) were chosen as the target Circulating Tumor Cells (CTCs) for the separation tests, because this cell line, along with two other breast cancer cell lines (T-47D and MDA-MB-231), accounts for more than two-thirds of the reported breast cancer models in literature [73, 74]. MCF-7 specifically binds to the antibody (Ep-CAM antibody (9C4): sc-21792[1]). As mentioned in Section 3.5, the density and diameter of CTCs were reported to be ~ 1700 Kg/m^3 [75] and $\sim 20\ \mu m$ [76], respectively. White Blood Cells (WBCs) have a density and diameter of $\sim 1080\ Kg/m^3$ and $\sim 12\ \mu m$, respectively [77]. Therefore, the mouse fibroblast cell line (L929) with a similar density and size to WBCs was chosen as non-target cells, which is also in line with the literature [78-81]. Figure S5.1a-b shows the cultured cells of MCF-7 and L929.

[1] https://www.scbt.com/p/ep-cam-antibody-9c4

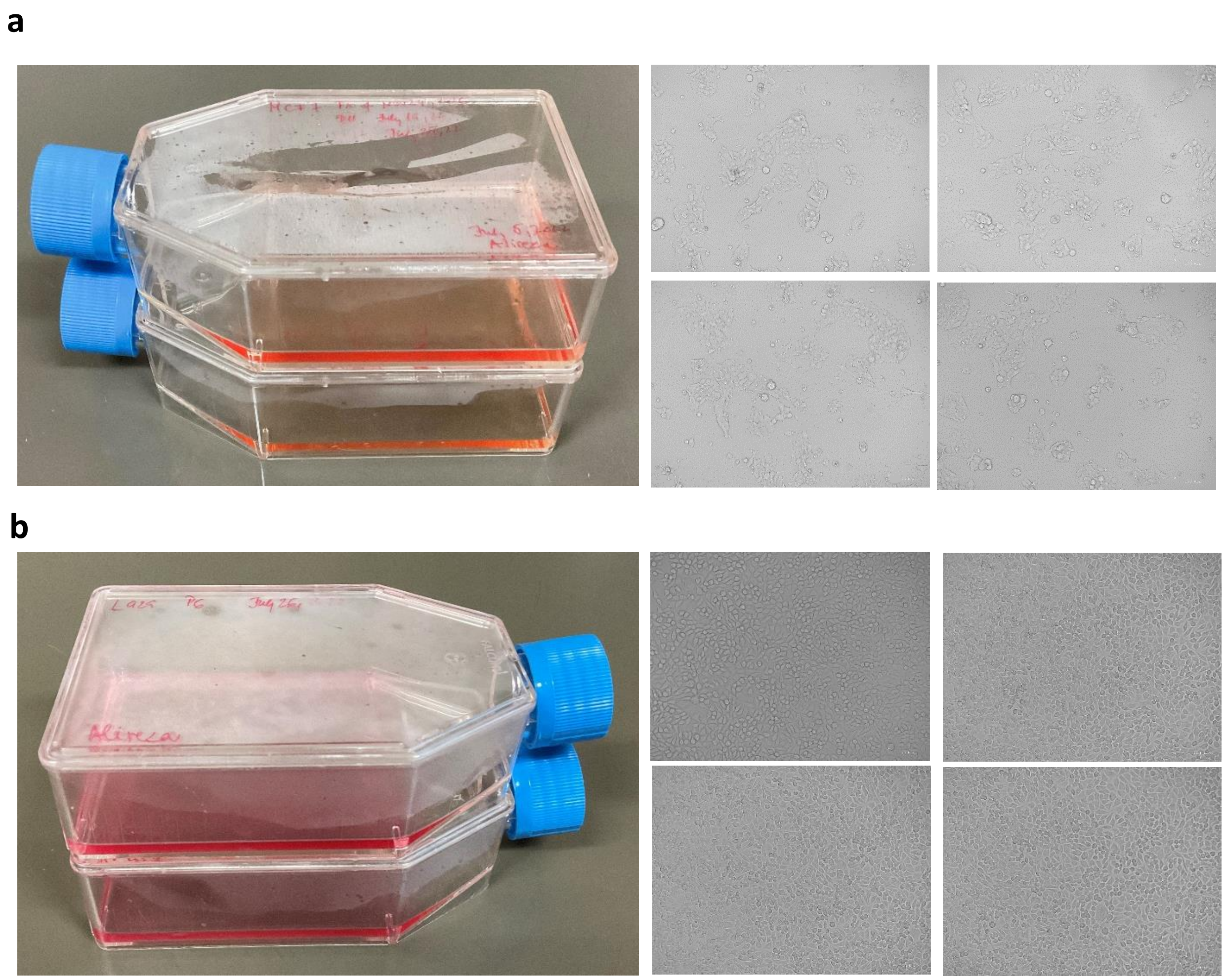

Figure S5.1: *The cultured cells in different flasks along with microscopic images of their population density;* ***a:*** *MCF-7; and* ***b:*** *L929 cells attached to the bottom of the flask.*

Before starting the active separation of CTCs under a magnetic field, it is important to ensure that (1) the target cells are magnetized (so that they can be differentiated in the magnetic field effects) and (2) the device functions properly. The test for (1) is presented in Section S.5.1 and the test for (2) is presented in Section S.5.2.

S5.1 Positive and negative tests

Two tests were performed related to (1): positive and negative tests. The positive test inspects whether the antibodies attach to MCF-7, and the negative test inspects whether the antibodies do not connect to non-target cells (L929 in this study). It is noted that antibody-antigen binding is exclusive, which means that the antibody will only bind to its corresponding antigen. In this study, these antibodies were expected to attach to MCF-7 only not to L929. Specifically, two samples (CTCs and L929) were prepared. Each of them was passaged 2 times, and then was mixed

with Iron Oxide Nanoparticles (IONPs)-capped antibodies. After that, they were mixed, and examined under a cell imager microscope (Figure S5.2). It is noted that although the size of antibodies is in the nanoscale range and cannot be seen with an optical microscope, their attached IONPs can be visible under a microscope. It can be seen from Figure S5.2a that IONPs were gathered around the MCF-7 cells. The image in Figure S5.2a confirms the binding of the antibodies to the CTC cells. To confirm the result observed under the microscope is reliable, a strong magnet was placed adjacent to the sample, and it was observed that the IONPs-attached cells moved toward the magnet. The antibodies were combined in a similar manner with L929 for the negative control test, as mentioned before. The image shown in Figure S5.2b indicates that the antibodies have not bonded to these cells. Overall, the two control tests confirm that the target cells are magnetized, related to (1).

a

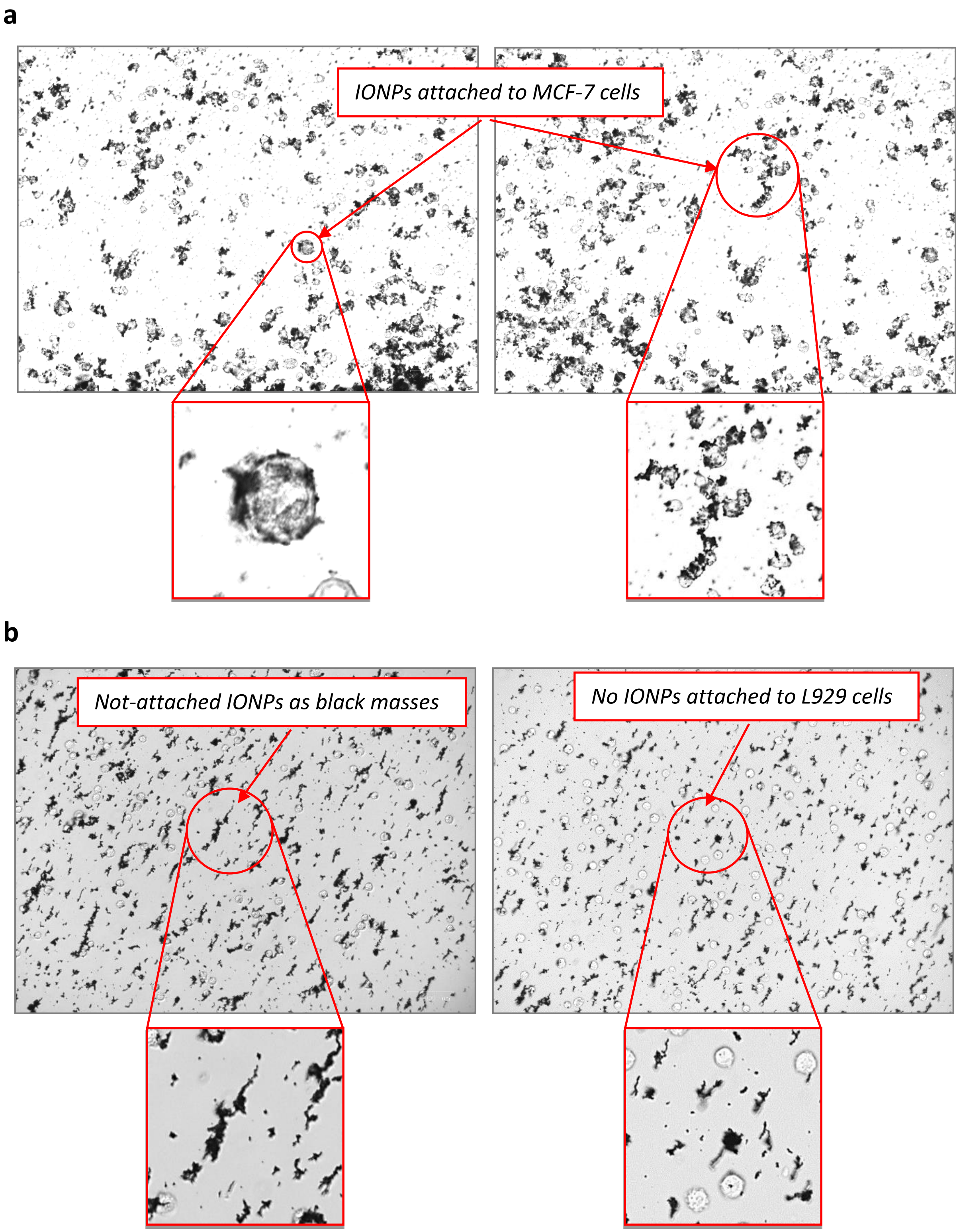

Figure S5.2: *The images taken by the microscope of MCF-7 and L929 cells after mixing with IONPs-connected antibodies;* ***a:*** *Positive control test (the attachment of antibodies to CTCs can be seen through the adhesion of IONPs in the form of small black beads to the cells);* ***b:*** *Negative*

control test (the non-binding of IONPs-attached antibodies to non-target cells can be represented as the black spot due to the accumulation of IONPs).

S5.2 Device functionality test

The test for (2) is also called device functionality. This test was divided into two parts: (a) an inspection of the movement of a single type of cell under the effect of a magnetic field, and (b) a similar inspection as (a) but without the effect of the magnetic field. Before both tests, a Phosphate-Buffered Saline (PBS) solution was filled into the microchannel with three purposes: (1) to ensure the surface tension effect to be very small to be negligible, (2) to prevent the formation of air bubbles inside the microchannel, and (3) to slow down the movement of cells such that the assumption of laminar flow is valid. After that, the two parts of the test were conducted.

With respect to (a), the device performance was inspected in terms of cell movement (under the condition that there is no interference with the other type of cells) by injecting only one type of cell and monitoring whether this group of cells went to the desired target reservoir, Reservoir #3 in this case. Therefore, either CTCs or colored non-target cells could be filled into the inlet reservoir; however, at this point, CTCs were preferable because the final objective of this study was to capture the maximum CTCs from the target reservoir. One issue is to determine the number of CTCs to be filled, and the issue was resolved with a cell counting chamber. Further, the test was performed by mounting the holder-placed disk onto the motor head and spinning it clockwise at 2500 RPM for 2 s, followed by a 5-second counter-clockwise rotation at 1750 RPM (Figure S5.3). These rotational velocities were chosen based on the observation of the simulation result for the maximum cell separation efficiency (see Section 3.7). After that, the contents of the target reservoir (i.e., Reservoir #3, see Figure 3) and non-target reservoir (i.e., Reservoir #2, see Figure 3) were inspected under a fluorescence microscope (Figure S5.3).

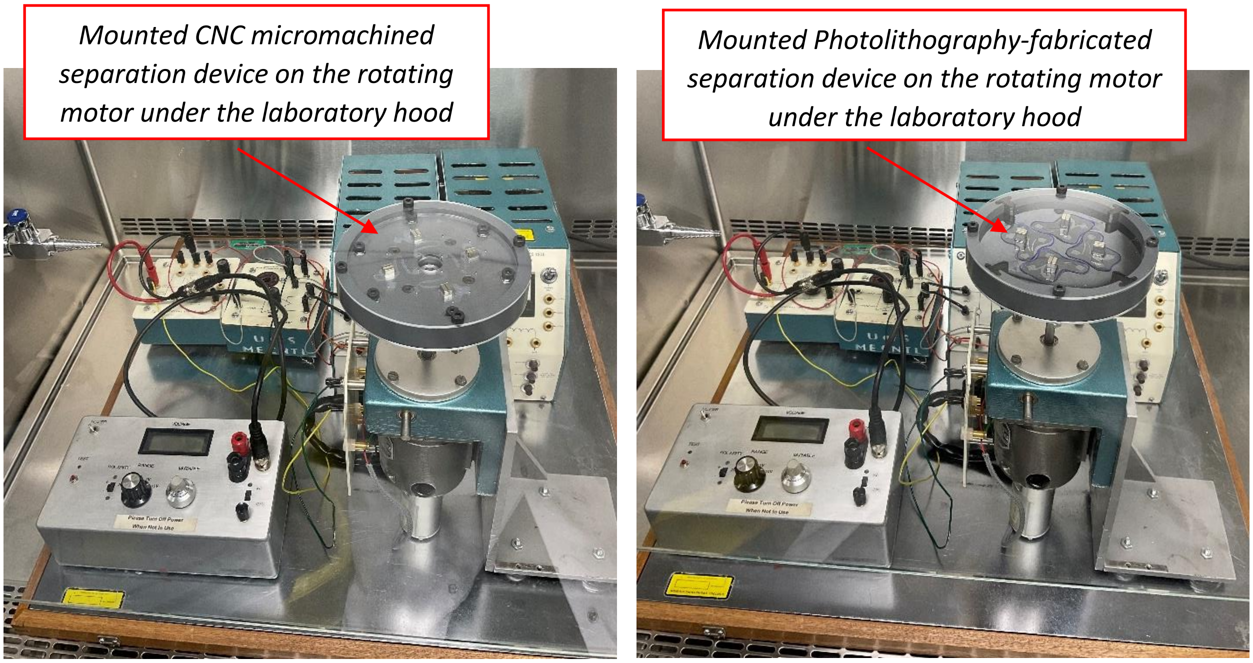

Figure S5.3: *Image of the on-disk microfluidic device mounted onto the rotating motor.*

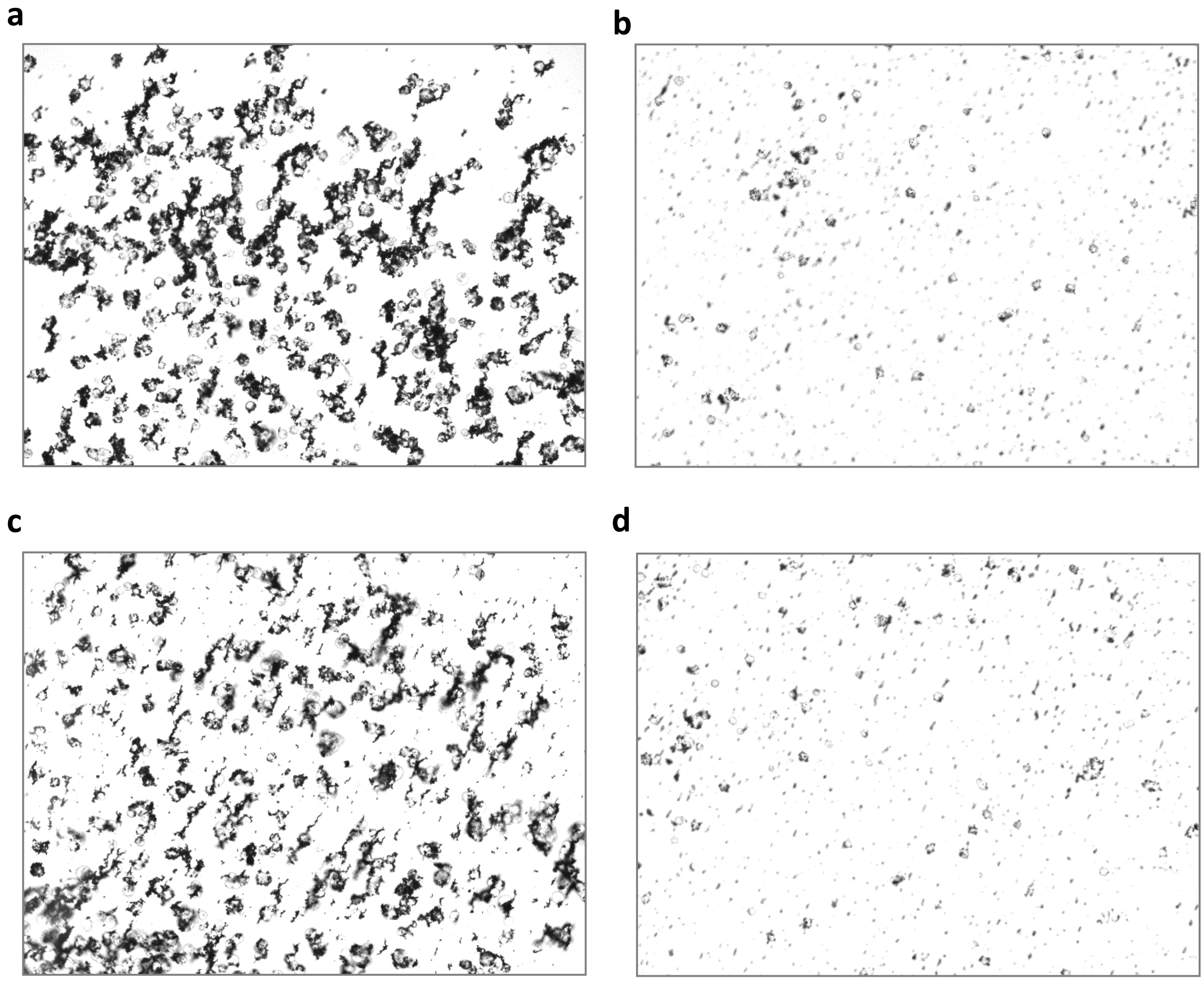

Figure S5.4: *Photomicrographs of target and non-target reservoirs after the experiment with MCF-7;* ***a:*** *Target reservoir of photolithography-based centrifugal device,* ***b:*** *Non-target reservoir of the photolithography-based centrifugal device;* ***c:*** *Target reservoir of the CNC-micromachined centrifugal device,* ***d:*** *Non-target reservoir of the CNC-micromachined centrifugal device.*

It can be seen from Figure S5.4a and Figure S5.4c that a good percentage of CTCs entered the target reservoir (i.e., Reservoir #3, see Figure 3). This thus ensures the accuracy and performance of the separation system. Figure S5.4b and Figure S5.4d also confirm that the target CTCs have followed the desired path well, so that only a few of them entered the non-target reservoir (i.e., Reservoir #2, see Figure 3). It should be noted that these conclusions do not mean that the same results should be expected in separating two types of cells (when there is a mixture of different cells).

Part (b) of the test was carried out using the CTC-containing sample. This time, the system started to rotate under the same condition but without magnets. The only difference between Part (a) and Part (b) of the test was the elimination of the magnets from the platform. The good sign observed under the microscope was that none of the target cells had entered the target reservoir (i.e., Reservoir #3, see Figure 3), and they had all been pulled by centrifugal and Coriolis forces into the non-target reservoir (Reservoir #2, see Figure 3), which is consistent with the theoretical account (i.e., due to the lack of effective magnetic force).

Nomenclature

Parameter	Name
**Arg**	Arginine
**CBO**	Cross-Beam Optics
**CD**	Compact Disk
**CEA**	Contraction–Expansion Array
**CLS**	Canadian Light Source
**CNC**	Computer Numerical Control
**CTC**	Circulating Tumor Cell
**DC**	Direct Current
**DEP**	Dielectrophoresis
**DLD**	Deterministic Lateral Displacement
**dTG**	Derivative Thermogravimetric Analysis
**EDC**	1-Ethyl-3-(3-Dimethylaminopropyl) Carbodiimide
**FCC**	Face-Centered Cubic
**IDA**	Iron Deficiency Anemia
**IONP**	Iron Oxide Nanoparticle
**MA**	Arg-Coated IONPs
**MAE**	MA mixed with EDC
**MAEN**	MAE mixed with NHS
**MAENAb**	MAEN mixed with Antibodies
**MNPs**	Magnetic Nanoparticles
**MOFF**	Multi-Orifice Flow Fractionation
**MW**	Molecular Weight
**NHS**	N-Hydroxysuccinimide
**PBS**	Phosphate-Buffered Saline
**PC**	Polycarbonate
**PDMS**	Polydimethylsiloxane
**PFF**	Pinched Flow Fractionation
**PMMA**	Poly(Methyl Methacrylate)
**PVC**	Polyvinyl Chloride
**RIE**	Reactive Ion Etching
**RPM**	Revolutions Per Minute
**SSSC**	Saskatchewan Structural Sciences Center
**TGA**	Thermogravimetric Analysis
**WBC**	White Blood Cell
**XRD**	X-Ray Diffraction

References

1. Soetaert, F.; Korangath, P.; Serantes, D.; Fiering, S.; Ivkov, R., Cancer therapy with iron oxide nanoparticles: Agents of thermal and immune therapies. *Adv Drug Deliv Rev* **2020,** 163-164, 65-83.
2. Xu, H.; Aguilar, Z. P.; Yang, L.; Kuang, M.; Duan, H.; Xiong, Y.; Wei, H.; Wang, A., Antibody conjugated magnetic iron oxide nanoparticles for cancer cell separation in fresh whole blood. *Biomaterials* **2011,** 32, (36), 9758-9765.
3. Ling, D.; Hyeon, T., Chemical Design of Biocompatible Iron Oxide Nanoparticles for Medical Applications. *Small* **2013,** 9, (9-10), 1450-1466.
4. López Pérez, J. A.; López Quintela, M. A.; Mira, J.; Rivas, J.; Charles, S. W., Advances in the Preparation of Magnetic Nanoparticles by the Microemulsion Method. *The Journal of Physical Chemistry B* **1997,** 101, (41), 8045-8047.
5. Wang, L.; Bao, J.; Wang, L.; Zhang, F.; Li, Y., One-Pot Synthesis and Bioapplication of Amine-Functionalized Magnetite Nanoparticles and Hollow Nanospheres. *Chemistry – A European Journal* **2006,** 12, (24), 6341-6347.
6. Ge, J.; Hu, Y.; Biasini, M.; Beyermann, W. P.; Yin, Y., Superparamagnetic Magnetite Colloidal Nanocrystal Clusters. *Angewandte Chemie International Edition* **2007,** 46, (23), 4342-4345.
7. Tao, K.; Dou, H.; Sun, K., Facile Interfacial Coprecipitation To Fabricate Hydrophilic Amine-Capped Magnetite Nanoparticles. *Chemistry of Materials* **2006,** 18, (22), 5273-5278.
8. Sun, S.; Zeng, H., Size-Controlled Synthesis of Magnetite Nanoparticles. *Journal of the American Chemical Society* **2002,** 124, (28), 8204-8205.
9. Li, Y.; Afzaal, M.; O'Brien, P., The synthesis of amine-capped magnetic (Fe, Mn, Co, Ni) oxide nanocrystals and their surface modification for aqueous dispersibility. *Journal of Materials Chemistry* **2006,** 16, (22), 2175-2180.
10. Massart, R., Preparation of aqueous magnetic liquids in alkaline and acidic media. *IEEE transactions on magnetics* **1981,** 17, (2), 1247-1248.
11. Hyeon, T., Chemical synthesis of magnetic nanoparticles. *Chemical Communications* **2003**, (8), 927-934.
12. Willson, C., Arginine. In *Reference Module in Biomedical Sciences*, Elsevier: 2015.
13. B. Bolger, M., Chapter 9 - Computational Techniques in Macromolecular Structural Analysis. In *Introduction to Biophysical Methods for Protein and Nucleic Acid Research*, Glasel, J. A.; Deutscher, M. P.; Deutscher, M. P., Eds. Academic Press: San Diego, 1995; pp 433-490.
14. Azizi, K.; Karimi, M.; Shaterian, H. R.; Heydari, A., Ultrasound irradiation for the green synthesis of chromenes using L-arginine-functionalized magnetic nanoparticles as a recyclable organocatalyst. *RSC Advances* **2014,** 4, (79), 42220-42225.
15. Din, M. I.; Zahoor, A.; Hussain, Z.; Khalid, R., A review on green synthesis of iron (Fe) nanomaterials, its alloys and oxides. *Inorganic and Nano-Metal Chemistry* **2022,** 52, (1), 20-36.
16. Ortega, G.; Reguera, E., Chapter 13 - Biomedical applications of magnetite nanoparticles. In *Materials for Biomedical Engineering*, Holban, A.-M.; Grumezescu, A. M., Eds. Elsevier: 2019; pp 397-434.
17. Wang, Z.; Zhu, H.; Wang, X.; Yang, F.; Yang, X., One-pot green synthesis of biocompatible arginine-stabilized magnetic nanoparticles. *Nanotechnology* **2009,** 20, (46), 465606.

18. Bagherpour, A.; Kashanian, F.; Ebrahimi, S. S.; Habibi-Rezaei, M., L-arginine modified magnetic nanoparticles: green synthesis and characterization. *Nanotechnology* **2018,** 29, (7), 075706.
19. Rehana, D.; Haleel, A. K.; Rahiman, A. K., Hydroxy, carboxylic and amino acid functionalized superparamagnetic iron oxide nanoparticles: Synthesis, characterization and in vitro anti-cancer studies. *Journal of Chemical Sciences* **2015,** 127, (7), 1155-1166.
20. Lai, Y.; Yin, W.; Liu, J.; Xi, R.; Zhan, J., One-pot green synthesis and bioapplication ofl-arginine-capped superparamagnetic Fe3O4 nanoparticles. *Nanoscale research letters* **2010,** 5, (2), 302-307.
21. Ünal, B.; Durmus, Z.; Baykal, A.; Sözeri, H.; Toprak, M.; Alpsoy, L., L-Histidine coated iron oxide nanoparticles: synthesis, structural and conductivity characterization. *Journal of Alloys and Compounds* **2010,** 505, (1), 172-178.
22. Bagherpour, A. R.; Kashanian, F.; Seyyed Ebrahimi, S. A.; Habibi-Rezaei, M., L-arginine modified magnetic nanoparticles: green synthesis and characterization. *Nanotechnology* **2018,** 29, (7), 075706.
23. Tapiero, H.; Mathe, G.; Couvreur, P.; Tew, K., I. Arginine. *Biomedicine & pharmacotherapy* **2002,** 56, (9), 439-445.
24. Amaria, A.; Nuryono, N.; Suyanta, S., Preparation of L-arginine-modified silica-coated magnetite nanoparticles for Au (III) adsorption. *Orient. J. Chem* **2017,** 33, 384-395.
25. Hong, R.; Fischer, N. O.; Emrick, T.; Rotello, V. M., Surface PEGylation and ligand exchange chemistry of FePt nanoparticles for biological applications. *Chemistry of materials* **2005,** 17, (18), 4617-4621.
26. Liu, X.; Guan, Y.; Ma, Z.; Liu, H., Surface modification and characterization of magnetic polymer nanospheres prepared by miniemulsion polymerization. *Langmuir* **2004,** 20, (23), 10278-10282.
27. Euliss, L. E.; Grancharov, S. G.; O'Brien, S.; Deming, T. J.; Stucky, G. D.; Murray, C.; Held, G., Cooperative assembly of magnetic nanoparticles and block copolypeptides in aqueous media. *Nano Letters* **2003,** 3, (11), 1489-1493.
28. Yuan, X., *Non-Viral Gene Therapy*. BoD–Books on Demand: 2011.
29. Nataliia V. Shchotkina, A. A. S., Liudmyla V. Dolinchuk, Oleksandr Yu. Galkin, Glib I. Glib I., Dmytro A. Yemets, Anatoliy A. Sokol, Arkadii A. Dovghaliuk, Iryna M. Skorokhod, Olena V. Olena V., Nadiia M. Rudenko, Iliia M. Yemets, The Effect of Sterilization on the Bovine Pericardium Scaffold Decellularized By the Glutaraldehyde-Free Technology *Journal of Biomedical Engineering and Biosciences* **2021,** 8, 28-35.
30. Vashist, S. K., Comparison of 1-Ethyl-3-(3-Dimethylaminopropyl) Carbodiimide Based Strategies to Crosslink Antibodies on Amine-Functionalized Platforms for Immunodiagnostic Applications. *Diagnostics (Basel)* **2012,** 2, (3), 23-33.
31. Torres, M.; Casadevall, A., The immunoglobulin constant region contributes to affinity and specificity. *Trends in Immunology* **2008,** 29, (2), 91-97.
32. Heyward, C. Y.; Dong, L.; Shakhzadyan, H.; Wan, C.; Stokol, T., Detection of Epithelial Cell Adhesion Molecule in Feline Normal and Tumor Cell Lines and Tissues With Selected Commercial Anti-human EpCAM Antibodies. *Frontiers in Veterinary Science* **2021,** 8.

33. Kang, Y. S.; Risbud, S.; Rabolt, J. F.; Stroeve, P., Synthesis and Characterization of Nanometer-Size Fe_3O_4 and γ-Fe_2O_3 Particles. *Chemistry of Materials* **1996,** 8, (9), 2209-2211.
34. Kindvall, A. Improvements in thin film CdTe back contact and interface layers through sputter deposition of metals and semiconductor materials. Colorado State University, 2019.
35. Ling, D.; Hyeon, T., Iron Oxide Nanoparticles: chemical design of biocompatible iron oxide nanoparticles for medical applications (Small 9–10/2013). *Small* **2013,** 9, (9-10), 1449-1449.
36. Sun, J.; Zhou, S.; Hou, P.; Yang, Y.; Weng, J.; Li, X.; Li, M., Synthesis and characterization of biocompatible Fe_3O_4 nanoparticles. *Journal of biomedical materials research Part A* **2007,** 80, (2), 333-341.
37. McIntyre, N. S.; Zetaruk, D. G., X-ray photoelectron spectroscopic studies of iron oxides. *Analytical Chemistry* **1977,** 49, (11), 1521-1529.
38. Ozkaya, T.; Toprak, M. S.; Baykal, A.; Kavas, H.; Köseoğlu, Y.; Aktaş, B., Synthesis of Fe_3O_4 nanoparticles at 100 C and its magnetic characterization. *Journal of Alloys and Compounds* **2009,** 472, (1-2), 18-23.
39. Scherrer, P., Bestimmung der Grösse und der inneren Struktur von Kolloidteilchen mittels Röntgenstrahlen. *Nachrichten von der Gesellschaft der Wissenschaften zu Göttingen, mathematisch-physikalische Klasse* **1918,** 1918, 98-100.
40. Vinila, V. S.; Isac, J., Chapter 14 - Synthesis and structural studies of superconducting perovskite $GdBa_2Ca_3Cu_4O_{10.5+\delta}$ nanosystems. In *Design, Fabrication, and Characterization of Multifunctional Nanomaterials*, Thomas, S.; Kalarikkal, N.; Abraham, A. R., Eds. Elsevier: 2022; pp 319-341.
41. Aslam, M.; Schultz, E. A.; Sun, T.; Meade, T.; Dravid, V. P., Synthesis of Amine-Stabilized Aqueous Colloidal Iron Oxide Nanoparticles. *Crystal Growth & Design* **2007,** 7, (3), 471-475.
42. Ghasemi, E.; Ghahari, M., Synthesis of silica coated magnetic nanoparticles. *International Journal of Nanoscience and Nanotechnology* **2015,** 11, (2), 133-137.
43. Gupta, A. K.; Gupta, M., Synthesis and surface engineering of iron oxide nanoparticles for biomedical applications. *Biomaterials* **2005,** 26, (18), 3995-4021.
44. Coats, A.; Redfern, J., Thermogravimetric analysis. A review. *Analyst* **1963,** 88, (1053), 906-924.
45. Si, S.; Kotal, A.; Mandal, T. K.; Giri, S.; Nakamura, H.; Kohara, T., Size-Controlled Synthesis of Magnetite Nanoparticles in the Presence of Polyelectrolytes. *Chemistry of Materials* **2004,** 16, (18), 3489-3496.
46. Huebner, J., Antibody-Antigen Interactions and Measurements of Immunologic Reactions. In *Immunology, Infection, and Immunity*, 2004; pp 207-232.
47. Mason, D. W.; Williams, A. F., The kinetics of antibody binding to membrane antigens in solution and at the cell surface. *Biochemical Journal* **1980,** 187, (1), 1-20.
48. Lee, M. G.; Choi, S.; Park, J.-K., Inertial separation in a contraction–expansion array microchannel. *Journal of Chromatography A* **2011,** 1218, (27), 4138-4143.
49. Chueh, B.-h.; Huh, D.; Kyrtsos, C. R.; Houssin, T.; Futai, N.; Takayama, S., Leakage-free bonding of porous membranes into layered microfluidic array systems. *Analytical chemistry* **2007,** 79, (9), 3504-3508.
50. Rasouli, A. A step towards a new micro-fluidic switch valve with embedded instructions. University of Saskatchewan, Canada, 2018.

51. Kang, J. H.; Krause, S.; Tobin, H.; Mammoto, A.; Kanapathipillai, M.; Ingber, D. E., A combined micromagnetic-microfluidic device for rapid capture and culture of rare circulating tumor cells. *Lab on a Chip* **2012,** 12, (12), 2175-2181.
52. Farahinia, A.; Zhang, W.; Badea, I., Novel microfluidic approaches to circulating tumor cell separation and sorting of blood cells: A review. *Journal of Science: Advanced Materials and Devices* **2021,** 6, (3), 303-320.
53. Farahinia, A.; Zhang, W.; Badea, I., Recent Developments in Inertial and Centrifugal Microfluidic Systems along with the Involved Forces for Cancer Cell Separation: A Review. *Sensors* **2023,** 23, (11), 5300.
54. Di Carlo, D.; Edd, J. F.; Humphry, K. J.; Stone, H. A.; Toner, M., Particle segregation and dynamics in confined flows. *Physical review letters* **2009,** 102, (9), 094503.
55. Chin, C. D.; Linder, V.; Sia, S. K., Commercialization of microfluidic point-of-care diagnostic devices. *Lab on a Chip* **2012,** 12, (12), 2118-2134.
56. Walsh III, D. I.; Kong, D. S.; Murthy, S. K.; Carr, P. A., Enabling microfluidics: from clean rooms to makerspaces. *Trends in biotechnology* **2017,** 35, (5), 383-392.
57. Ren, K.; Chen, Y.; Wu, H., New materials for microfluidics in biology. *Current Opinion in Biotechnology* **2014,** 25, 78-85.
58. Nge, P. N.; Rogers, C. I.; Woolley, A. T., Advances in Microfluidic Materials, Functions, Integration, and Applications. *Chemical Reviews* **2013,** 113, (4), 2550-2583.
59. Aeinehvand, M. M.; Ibrahim, F.; harun, S. W.; Al-Faqheri, W.; Thio, T. H. G.; Kazemzadeh, A.; Madou, M., Latex micro-balloon pumping in centrifugal microfluidic platforms. *Lab on a Chip* **2014,** 14, (5), 988-997.
60. Siegrist, J.; Amasia, M.; Singh, N.; Banerjee, D.; Madou, M., Numerical modeling and experimental validation of uniform microchamber filling in centrifugal microfluidics. *Lab on a Chip* **2010,** 10, (7), 876-886.
61. Lafleur, J. P.; Salin, E. D., Pre-concentration of trace metals on centrifugal microfluidic discs with direct determination by laser ablation inductively coupled plasma mass spectrometry. *Journal of Analytical Atomic Spectrometry* **2009,** 24, (11), 1511-1516.
62. Ogilvie, I.; Sieben, V.; Floquet, C.; Zmijan, R.; Mowlem, M.; Morgan, H., Reduction of surface roughness for optical quality microfluidic devices in PMMA and COC. *Journal of Micromechanics and Microengineering* **2010,** 20, (6), 065016.
63. Lide, D. R., *CRC handbook of chemistry and physics*. CRC press: 2004; Vol. 85.
64. Godino, N.; Gorkin Iii, R.; Linares, A. V.; Burger, R.; Ducrée, J., Comprehensive integration of homogeneous bioassays via centrifugo-pneumatic cascading. *Lab on a Chip* **2013,** 13, (4), 685-694.
65. Duford, D. A.; Xi, Y.; Salin, E. D., Enzyme Inhibition-Based Determination of Pesticide Residues in Vegetable and Soil in Centrifugal Microfluidic Devices. *Analytical Chemistry* **2013,** 85, (16), 7834-7841.
66. Kar, S.; Ghosh, U.; Maiti, T. K.; Chakraborty, S., Haemoglobin content modulated deformation dynamics of red blood cells on a compact disc. *Lab on a Chip* **2015,** 15, (24), 4571-4577.
67. Jahromi, A. K.; Saadatmand, M.; Eghbal, M.; Yeganeh, L. P., Development of simple and efficient Lab-on-a-Disc platforms for automated chemical cell lysis. *Scientific reports* **2020,** 10, (1), 1-13.

68. Bamshad, A.; Nikfarjam, A.; Khaleghi, H., A new simple and fast thermally-solvent assisted method to bond PMMA–PMMA in micro-fluidics devices. *Journal of Micromechanics and Microengineering* **2016,** 26, (6), 065017.
69. Zhu, X.; Liu, G.; Guo, Y.; Tian, Y., Study of PMMA thermal bonding. *Microsystem Technologies* **2007,** 13, (3), 403-407.
70. Tennico, Y. H.; Koesdjojo, M. T.; Kondo, S.; Mandrell, D. T.; Remcho, V. T., Surface modification-assisted bonding of polymer-based microfluidic devices. *Sensors and Actuators B: Chemical* **2010,** 143, (2), 799-804.
71. Faghih, M. M.; Sharp, M. K., Solvent-based bonding of PMMA–PMMA for microfluidic applications. *Microsystem Technologies* **2019,** 25, (9), 3547-3558.
72. Sivakumar, R.; Lee, N. Y., Microfluidic device fabrication mediated by surface chemical bonding. *Analyst* **2020,** 145, (12), 4096-4110.
73. Dai, X.; Cheng, H.; Bai, Z.; Li, J., Breast Cancer Cell Line Classification and Its Relevance with Breast Tumor Subtyping. *J Cancer* **2017,** 8, (16), 3131-3141.
74. Lacroix, M.; Haibe-Kains, B.; Hennuy, B.; Laes, J. F.; Lallemand, F.; Gonze, I.; Cardoso, F.; Piccart, M.; Leclercq, G.; Sotiriou, C., Gene regulation by phorbol 12-myristate 13-acetate in MCF-7 and MDA-MB-231, two breast cancer cell lines exhibiting highly different phenotypes. *Oncol Rep* **2004,** 12, (4), 701-7.
75. Kirby, D.; Siegrist, J.; Kijanka, G.; Zavattoni, L.; Sheils, O.; O'Leary, J.; Burger, R.; Ducrée, J., Centrifugo-magnetophoretic particle separation. *Microfluidics and nanofluidics* **2012,** 13, (6), 899-908.
76. Arya, S. K.; Lee, K. C.; Rahman, A. R. A., Breast tumor cell detection at single cell resolution using an electrochemical impedance technique. *Lab on a Chip* **2012,** 12, (13), 2362-2368.
77. Grover, W. H.; Bryan, A. K.; Diez-Silva, M.; Suresh, S.; Higgins, J. M.; Manalis, S. R., Measuring single-cell density. *Proceedings of the National Academy of Sciences* **2011,** 108, (27), 10992-10996.
78. Norouzi, N.; Bhakta, H. C.; Grover, W. H., Sorting cells by their density. *PLoS One* **2017,** 12, (7), e0180520.
79. Size and Appearance of Cellular Elements. https://www.labce.com/spg28714_size_and_appearance_of_cellular_elements.aspx
80. Schmid-Schönbein, G. W.; Shih, Y. Y.; Chien, S., Morphometry of Human Leukocytes. *Blood* **1980,** 56, (5), 866-875.
81. Tigner, A.; Ibrahim, S. A.; Murray, I., Histology, white blood cell. **2020**.
